# Synthesis of New 5-Aryl-benzo[*f*][1,7]naphthyridines via a Cascade Process (Ugi-3CR/Intramolecular Aza-Diels-Alder Cycloaddition)/Aromatization

**DOI:** 10.3390/molecules23082029

**Published:** 2018-08-14

**Authors:** Óscar Vázquez-Vera, Daniel Segura-Olvera, Mónica A. Rincón-Guevara, Atilano Gutiérrez-Carrillo, Miguel A. García-Sánchez, Ilich A. Ibarra, Leticia Lomas-Romero, Alejandro Islas-Jácome, Eduardo González-Zamora

**Affiliations:** 1 Unidad Profesional Interdisciplinaria de Biotecnología, Instituto Politécnico Nacional, Av. Acueducto de Guadalupe S/N, Barr. La Laguna Ticomán, Gustavo A Madero C.P. 07340, Ciudad de México, Mexico; vaveos@yahoo.com.mx; 2 Departamento de Química, Universidad Autónoma Metropolitana-Iztapalapa, San Rafael Atlixco 186, Col. Vicentina, Iztapalapa C.P. 09340, Ciudad de México, Mexico; mudo-2@hotmail.com (D.S.-O.); agrmn@xanum.uam.mx (A.G.-C.); mags@xanum.uam.mx (M.A.G.-S.); 3 Departamento de Biotecnología, Universidad Autónoma Metropolitana-Iztapalapa, San Rafael Atlixco 186, Col. Vicentina, Iztapalapa C.P. 09340, Ciudad de México, Mexico; monicarinconguevara@gmail.com; 4 Laboratorio de Fisicoquímica y Reactividad de Superficies, Instituto de Investigaciones en Materiales, Universidad Nacional Autónoma de México, Circuito Exterior S/N, Ciudad Universitaria, Coyoacán C.P. 04510, Ciudad de México, Mexico; argel@unam.mx

**Keywords:** one-pot procedures, cascade processes, multicomponent reactions, aza-Diels-Alder cycloadditions, aromatization, microwave assisted synthesis, benzo[*f*][1,7]naphthyridines

## Abstract

A series of eight new 5-aryl-benzo[*f*][1,7]naphthyridines were synthesized in 17 to 64% overall yields via an improved MW-assisted cascade-like one pot process (Ugi–three component reaction/intramolecular aza-Diels-Alder cycloaddition) coupled to an aromatization process from tri-functional dienophile-containing ester-anilines, substituted benzaldehydes and the chain-ring tautomerizable 2-isocyano-1-morpholino-3-phenylpropan-1-one as starting reagents, under mild conditions. The doubly activated dienophile and the aza-diene functionalities of the eight new Ugi-adducts were exploited to perform an in situ aza-Diels-Alder cycloaddition/aromatization (dehydration/oxidation) process, toward the complex polysubstituted 5-aryl-polyheterocycles, which could be taken as starting point for further SAR studies because the benzo[*f*][1,7]naphthyridine is the core of various bioactive products. It is relevant to emphasize that the synthesis or isolation of benzo[*f*][1,7]naphthyridines containing a substituted aromatic ring in the C-5 position, has not been published before.

## 1. Introduction

**Figure 1:**
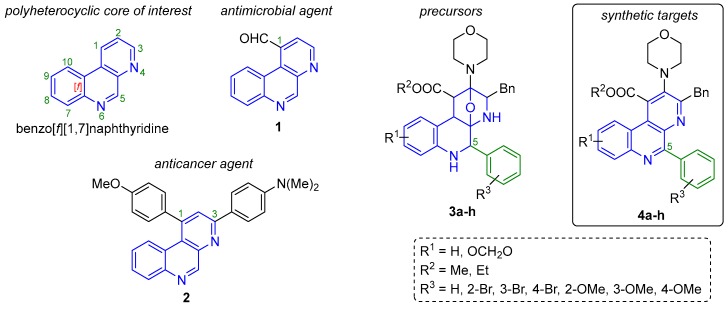
Benzo[*f*][1,7]naphthyridine and related compounds.

Benzo[*f*][1,7]naphthyridines [1] (Figure 1) are naturally occurring [2,3] and synthetic benzo-fused *bis*-heterocyclic systems [4], which have been of high interest for synthetic community from many years ago, mainly due to their interesting properties in various fields of science, such as optics [5]. However, their main application lies in the medicinal chemistry because some bioactive compounds are based on the benzo[*f*][1,7]naphthyridine core. As representative examples, benzo[*f*][1,7]naphthyridine-1-carbaldehyde (**1**, Figure 1) exhibited good MIC values against some pathogenic microbes like Gram negative bacteria (*E. coli* = 0.2 μg/mL; *P. aeruginosa* = 0.2 μg/mL; *K. pneumoniae* = 0.3 μg/mL), Gram positive bacteria (*L. monocytogenes* = 0.05 μg/mL; *S. aureus* = 0.1 μg/mL; *S. faecium* = 0.2 μg/mL) and pathogenic fungus (*C. albicans* = 0.05 μg/mL) [6]. In the same way, the 1,3-diaryl-benzo[*f*][1,7]naphthyridine **2** exhibited very interesting cytotoxicity GI_50_ values against human cell lines (lung: NCIH23 = 1.72 μM, colon: HCT15 = 1.86 μM, gastric: NUGC-3 = 1.95 μM, renal: ACHN = 1.98 μM, prostate: PC-3 = 2.98 μM, and breast: MDA-MB-231 = 2.30 μM), as well as outstanding human topoisomerase II_α_ inhibitory properties (91.3% at 100 μM) [7]. It is relevant to highlight the influence of the aromatic rings (and their substituents) at C-1 and C-3 positions from the compound **2** to improve the cytotoxicity. The present article describes an improved cascade multicomponent reaction/cycloaddition process to synthesize the oxa-bridged polyheterocycles **3a**–**h** (Figure 1), coupled to a full aromatization process towards the polyheterocyclic compounds **4a**–**h** (Figure 1). Both series are based on the benzo[*f*][1,7]naphthyridine core, containing an aromatic ring in the C-5 position, keeping in mind their potential application in optics and medicinal chemistry.

The typical methodology to synthesize benzo[*f*][1,7]naphthyridines involves a Skraup-type intramolecular cyclization from their corresponding 2-aminoazines and glycerol under sulfuric media [8,9]. Moreover, most of the reports describing the synthesis of this class of polyheterocyclic systems involve multistep (stepwise) strategies [10,11,12]. However, there are some undesirable features associated with most of this kind of methodologies, among which are worth highlighting: the typical low yields (proportional to the number of steps involved in the synthesis) and waste of resources such as man-hours, energy, solvents and materials for purification. Fortunately, there are various one-pot based synthetic strategies toward complex polyheterocycles, resulting in time-saving, good selectivity, optimization of resources and mainly, in high atom economy [13]. Among the most important one-pot processes, “*multicomponent reactions (MCRs) occupy a special place*” as stated masterfully by Yus in 2005 [14]. MCRs are defined as high convergent sequentially combinations of at least three reagents in a single experimental operation and, in the same reactor, to construct diverse molecular architectures, for example, complex polyheterocycles [15]. It is noteworthy that, MCRs are robust and efficient tools useful in Diversity Oriented Synthesis (DOS) [16], Combinatorial Chemistry (CC) [17] and Target Oriented Synthesis [18]. In this context, MCRs have been used successfully to synthesize series of benzo[*f*][1,7]naphthyridine-based compounds. Indeed, there are only two reports in the literature, both from our early research group in collaboration with Zhu [19,20]. The first one (2001) describes the synthesis of eight new oxa-bridged 5-alkyl-benzo[*f*][1,7]naphthyridines (structurally similar to our precursors **2**, Figure 1) via an Ugi-3CR/aza-Diels-Alder cycloaddition process under conventional heating conditions, using toluene as solvent and lithium bromide to activate the Schiff-bases involved in the Ugi-3CR mechanism [21], and hence, to promote the α-nucleophilic attack of the isocyanides to the involved iminium-like ions [22]. The second report (2007) was a continuation of our work, in which the best conditions to aromatize the oxa-bridged polyheterocyclic system were sought. Thus, trifluoroacetic acid in acetonitrile at 0 °C were the optimal conditions found toward eight new benzo[*f*][1,7]naphthyridines (structurally similar to our synthetic targets **3**, Figure 1). However, it is important to highlight that, both of our previously synthesized benzo[*f*][1,7]naphthyridine-based products contain alkylic substituents in the C-5 position because by using the optimal conditions then reported, it was not possible to synthesize any C-5-aryl-containing analogue. The Schiff-bases simply did not react with isocyanides. In this context, we became interested again in continuing those works, but now behind novel C-5-aryl-containing analogues mainly because it has been reported that aromatic rings of bioactive compounds often trigger, promote and/or improve electrostatic interactions, for instance, Stacking-type ones with biological substrates [7,23].

## 2. Results and Discussion

**Scheme 1:**
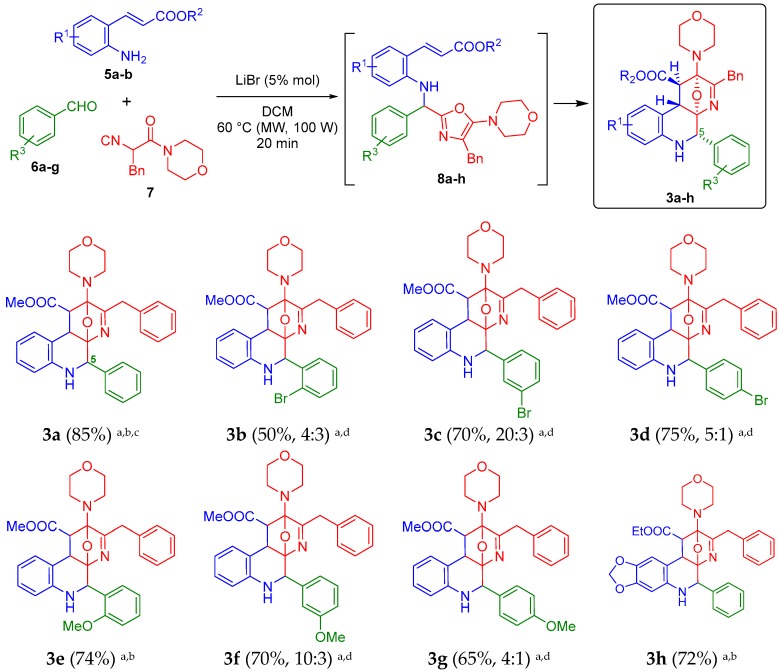
Synthesis of the new oxa-bridged 5-aryl-containing benzo[*f*][1,7]naphthyridines **3a**–**h**. ^a^ Yields measured after purification by silica-gel column chromatography; ^b^ Yields for the major diasteroisomer isolated; ^c^ 82% yield starting from 0.5 mmol of **5a**; ^d^ Yields for the inseparable diasteroisomeric mixture.

Considering the optimal reaction parameters found previously by us [19], we started the synthesis of the oxa-bridged 5-aryl-benzo[*f*][1,7]naphthyridines **3a**–**h**, taking the compound **3a** as the model to find the new optimal conditions (Scheme 1). Thus, the trifunctional methyl (*E*)-3-(2-aminophenyl)acrylate (**5a**), benzaldehyde (**6a**) and the chain-ring tautomerizable 2-isocyano-1-morpholino-3-phenylpropan-1-one (**7**) were combined sequentially in PhMe at 60 °C (conventional heating conditions), using 1.0 equiv. of LiBr. However, as expected, the first desired 5-aryl-containing oxa-bridged product **3a** could not be synthesized (nor its precursor, the Ugi-adduct **8a**). Conversely, various uncharacterized byproducts were detected in low amounts by TLC, including starting reagents and the corresponding Schiff-base. Thus, using again PhMe as solvent at 60 °C, we tested the next BrØnsted and Lewis acids: NH_4_Cl [24], PTSA [25], AlCl_3_ [26] or Sc(OTf)_3_ [27] because it has been reported their use to activate the Schiff-bases involved in Ugi-three component reactions, but all attempts toward **3a** were unsuccessful. In the same way, some experiments increasing the temperature over 60 °C were conducted, but decomposition was observed by TLC. Then, MeCN or DCM were used as solvents in combination with all the previously tested additives, but under MW-heating conditions, finding that, at 60 °C (100 W) and using DCM as the solvent and LiBr as the catalyst (0.05 instead 1.0 equiv.), the product **3a** was obtained in 85% yield as a mixture of separable diasteroisomers in 6 to 1 ratio, which is in concordance with those previously observed by us when aliphatic aldehydes were used as starting reagents (dr = 3 to 1) [19]. Thus, a 85% yield is considered as excellent based on the molecular complexity of the compound **3a**, and that until now, it had not been possible the synthesis or isolation anywhere of any oxa-bridged 5-aryl-containing benzo[*f*][1,7]naphthyridine. The role of MW-irradiation as heat source to trigger/facilitate [4+2] cycloaddition reactions (thermal pericyclic processes) toward oxa-bridged polyheterocycles is well known [28,29], and hence, it was crucial to decrease considerably the reaction times. Then, by taking the new optimized reaction parameters, we synthesized the compounds **3b**–**h**, but in all cases in lower yields (50–75%) in comparison to the product **3a** (85%). The substrate scope was evaluated by synthesizing bromine- and methoxy-containing analogues **3b**–**g** from *ortho*-, *meta*- and *para*-substituted benzaldehydes **6b**–**g**. Additionally, the di-oxa-methylene containing analogue **3h** was synthesized in a true good yield (74%). This product contains an ethyl ester moiety instead of methyl ester. Conversely to the model product **3a** (85%), the lowest yield was observed for the product **3b** (*o*-Br analogue), probably due to a bulky effect induced by the halogen atom that may hinder the cycloaddition process. In the same way, conversely to the product **3a** (a mixture of separable diasteroisomers), the product **3b** was obtained as a mixture of inseparable diasteroisomers (same *R*_f_ measured by TLC) in a 4 to 3 ratio (see the ^1^H-NMR spectrum in the Electronic Supplementary Material for further details), may be due also to the bulky effect coming from the *o*-Br aryl moiety during the cycloaddition process [20] which, in turn, could explain the lower yields with respect to **3a**. The same behavior was observed for the oxa-bridged products **3c** (dr = 20 to 3), **3d** (dr = 5 to 1), **3f** (dr = 10 to 3) and **3g** (dr = 4 to 1). It is important to mention that, various experiments trying to isolate the Ugi-adducts **8a**–**h** were performed, but, the intramolecular aza Diels-Alder cycloadditions took place immediately at 60 °C giving the oxa-bridged 5-aryl-containing benzo[*f*][1,7]naphthyridines **3a**–**h**. 

The products **3a**–**h** were fully characterized using IR, HRMS, ^1^H- and ^13^C-NMR techniques (see the Electronic Supplementary Material for further details), with the exception of the product **3b**, which could not been characterized by ^13^C-NMR due to an unexpected decomposition during the data acquisition time. However, the product **3b** was characterized by IR, ^1^H-NMR and HRMS. A distinguishing peak was observed at *m/z* = 588.1483 [calcd for (**3b**) C_31_H_31_BrN_3_O_4_^+^ [M + H]^+^ 588.1492].

**Scheme 2:**
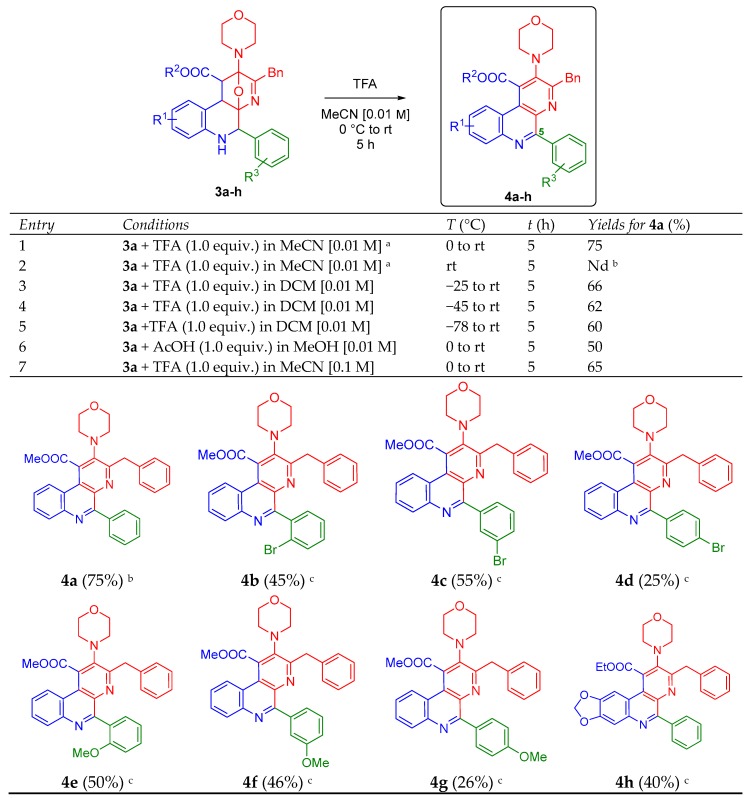
Synthesis of the new 5-aryl-containing benzo[*f*][1,7]naphthyridines **4a**–**h**. ^a^ Optimal reaction conditions; ^b^ Decomposition was observed, Nd = Not determined; ^c^ Yields measured after purification by silica-gel column chromatography, starting reagents **3** were recovered.

Having in hand the series of oxa-bridged 5-aryl-containing benzo[*f*][1,7]naphthyridines **3a**–**h**, we went behind the optimal conditions to aromatize the polyheterocyclic system via an acid-catalyzed dehydration/oxidation process toward the 5-aryl-containing benzo[*f*][1,7]naphthyridines **4a**–**h**, taking the compound **4a** as the model for the screening (Scheme 2). Thus, the study initiated considering the optimal reaction parameters reported previously by us [20], and again, as it was expected, the product **4a** was synthesized in a good yield (75%), entry 1. Then, we performed the reaction at room temperature trying to increase the yields, but several non-characterized by-products were observed by TLC, entry 2. Contrarily, experiments at colder temperatures (−25, −45 and −78 °C) in DCM were carried out, but no significant changes were observed, entries 3–5. In the same way, a system AcOH/MeOH was used (entry 6), but the TFA (1.0 equiv.) in MeCN [0.01 M] gave us the best results, entry 1. The effect of increasing the concentration was also evaluated [0.1 M], but the yield decreased little, entry 7. Thus, the series of 5-aryl-containing benzo[*f*][1,7]naphthyridines **4b**–**h** were synthesized using the optimal parameters, but in lower yields (26–55%) with respect to the model product **4a** (Scheme 2). As seen, the stereoelectronic nature of the substituents did not affect substantially the efficiency of the aromatization process. There was no a clear trend. In the same way, the yields did not change after 5 h in all cases. All products **4a**–**h** were fully characterized using IR, HRMS, ^1^H- and ^13^C-NMR techniques (see the Electronic Supplementary Material for further details). It is important to mention that despite several attempts were conducted to obtain adequate crystals for X-ray analysis of the products **4a**–**h** (or at least for one of their precursors **3a**–**h**), all were unsuccessful.

Regarding the reaction mechanisms, we assume that a similar sequence of events to our previous works [19,20] occurs to construct the 5-aryl-containing benzo[*f*][1,7]naphthyridines because the aromatic nature of the substituent in the C-5 position, in principle, is not enough to direct the reaction course for other pathways. In the same context, the relative stereochemistry of the oxa-bridged precursors **3a–h** matches with the products from our previous works [19,20] because a coupling constant nearly to 4.5 Hz are observed. Indeed, that value indicates a gauche relationship between the protons coming from the dienophile-containing ester-anilines. 

**Scheme 3:**
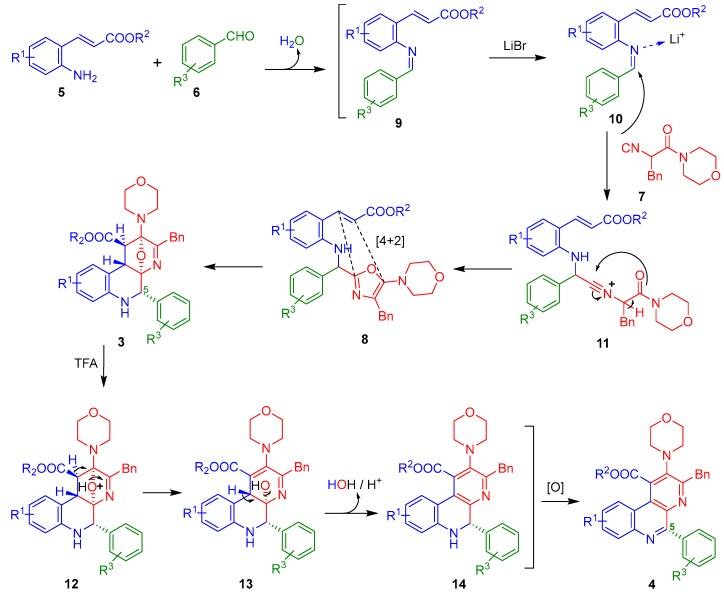
Reaction mechanism.

Thus, the amines **5** react with aldehydes **6** to give the Schiff bases **9**, which are activated by the Li^+^ to give the iminium-like ions **10**. These latter ones are α-nucleophilically attacked by the isocyanide **7** to afford the nitrilium ions **11**, which after a chain-ring tautomerization give the 5-aminooxazoles (Ugi-adducts) **8**. Then, the doubly activated dienophile and the aza-diene from **8** undergo an in situ aza-Diels-Alder cycloaddition to afford the isolated oxa-bridged 5-aryl-containing benzo[*f*][1,7]naphthyridines **3**, which after an addition of TFA, give the full aromatic 5-aryl-containing benzo[*f*][1,7]naphthyridines **4** via a dehydration of their precursors **13** followed by a spontaneous oxidation of **14**, both processes triggered by the acidic media (Scheme 3).

## 3. Experimental Section

### 3.1. General Information, Instrumentation, Software, Solvents and Chemicals

^1^H- and ^13^C-Nuclear Magnetic Resonance (NMR) spectra were acquired at room temperature (25 °C) on a 500 MHz AMX Advance III spectrometer (Bruker, Fällande, Uster, Switzerland). The solvent used for NMR experiments was deuterated chloroform (CDCl_3_). Chemical shifts are reported in parts per million (δ/ppm). Coupling constants are reported in Hertz (*J*/Hz). Internal reference for NMR spectra was tetramethylsilane (TMS) at 0.00 ppm. Multiplicities of the signals are reported using the standard abbreviations: singlet (s), doublet (d), triplet (t), quartet (q) and multiplet (m). NMR spectra were analyzed using the MestReNova software (Ver. 12.0.0-20080). Reported NMR spectra are for the major/isolated diasteroisomers. Infrared (IR) spectra were acquired on a Perkin Elmer 1600 spectrometer (Norwalk, CT, USA) using the Attenuated Total Reflectance (ATR) method. The absorbance peaks are reported in reciprocal centimeters (υ_max_/cm^−1^). IR spectra were analyzed using the Report Builder software (Ver. 2.01). High Resolution Mass Spectroscopy (HRMS) spectra were acquired by Electrospray ionization (ESI) on a Micro-TOF II spectrometer Bruker Daltonics GmbH (Bremen, Germany). HRMS samples were injected directly (Apollo source) and analyzed by Time of Flight (TOF). HRMS spectra were analyzed using the Compass 1.5 analysis software. Melting points were determined on a Fisher-Johns apparatus (Suwanee, GA, USA) and are uncorrected. Microwave assisted reactions were performed in closed vessel mode on a CEM Discover MW-reactor (Matthews, NC, USA). Reaction progress was monitored by Thin Layer Chromatography (TLC) and the spots were visualized under Ultraviolet (UV) light (254 or 365 nm). Flash columns packed with silica-gel 60 and glass preparative plates (20 × 20 cm) coated with silica-gel 60 doped with UV indicator (F254) were used to purify the products. Mixtures in 3:1 (*v*/*v*) proportion of hexanes (Hex) and ethyl acetate (EtOAc) were used to run TLC, silica-gel columns, preparative plates and to measure the Retention Factor (*R*_f_) values (using the same mobile phase for all the experiments). All starting reagents and solvents were used as received (without further purification, distillation nor dehydration). Chemical structures were drawn using the ChemDraw Professional software (Ver. 15.0.0.106).

### 3.2. Synthesis and Characterization of the Oxa-Bridged 5-arylbenzo[f][1,7]naphthyridines ***3a**–**h***

General procedure 1 (GP 1)

The corresponding amine (0.1 mmol, 1.0 equiv.) was diluted in anhydrous dichloromethane [0.1 M] in a 10 mL sealed CEM Discover microwave reaction tube. Then, the corresponding aldehyde (1.2 equiv.) was added. The mixture was stirred and MW-irradiated (65 °C, 100 W) for 10 min and then, lithium bromide (0.05 equiv.) and the corresponding isocyanide (1.1 equiv.) were sequentially added. The new reaction mixture was stirred and MW-irradiated (65 °C, 100 W) for additional 10 min. Then, water (5 mL) was added and the combined layers were placed into a Corning separation flask (125 mL), where vigorous extractions using dichloromethane (3 × 5 mL) were performed. The organic phase was dried using anhydrous sodium sulphate and filtered over a celite pad (1 cm). The solvent was removed to dryness under vacuum. The crude residue was purified immediately using a silica-gel column chromatography using a mixture of Hex–EtOAc in 3:1 (*v*/*v*) proportion as mobile phase to afford the corresponding products **3a**–**h**.

#### 3.2.1. *Methyl 3-Benzyl-2-morpholino-5-phenyl-1,5,6,10b-tetrahydro-2H-2,4a-epoxybenzo[f][1,7]-naphthyridine-1-carboxylate* (**3a**)

According to GP 1, methyl (*E*)-3-(2-aminophenyl)acrylate (17.7 mg), benzaldehyde (12.2 μL), LiBr (4.3 mg) and 2-isocyano-1-morpholino-3-phenylpropan-1-one (26.9 mg) were reacted together in DCM (1.0 mL) to afford the product **3a** (43.3 mg, 85%) as a yellow solid; m.p. = 138–140 °C; *R*_f_ = 0.42 (Hex–EtOAc = 3/1, *v*/*v*); FT–IR (ATR)υ_max_/cm^−1^ 1494, 1736, 2848, 3320; ^1^H-NMR: δ 2.39–2.43 (m, 2H), 2.79–2.83 (m, 2H), 3.20 (d, *J* = 4.5 Hz, 1H), 3.46 (d, *J* = 4.4 Hz, 1H), 3.52–3.62 (m, 5H), 3.74 (d, *J* = 16.4 Hz, 1H), 3.80 (s, 3H), 5.09 (s, 1H), 6.64–6.66 (m, 1H), 6.75–6.79 (m, 1H), 6.99–7.01 (m, 2H), 7.05–7.06 (m, 1H), 7.12–7.14 (m, 3H), 7.32–7.37 (m, 4H), 7.61–7.63 (m, 2H); ^13^C-NMR: δ 35.2, 47.8, 48.2, 52.6 (2), 58.7, 67.0, 97.2, 106.1, 115.7, 119.4, 123.0, 126.6, 127.6, 127.9, 128.2, 128.4, 128.9, 129.3, 130.0, 135.6, 137.8, 143.0, 171.0, 177.8; HRMS (EI): calcd. for C_31_H_32_N_3_O_4_^+^ 510.2393, found 510.2397.

#### 3.2.2. *Methyl 3-Benzyl-5-(2-bromophenyl)-2-morpholino-1,5,6,10b-tetrahydro-2H-2,4a-epoxybenzo[f][1,7]-naphthyridine-1-carboxylate* (**3b**)

According to GP 1, methyl (*E*)-3-(2-aminophenyl)acrylate (17.7 mg), 2-bromobenzaldehyde (14.0 μL), LiBr (4.3 mg) and 2-isocyano-1-morpholino-3-phenylpropan-1-one (26.9 mg) were reacted together in DCM (1.0 mL) to afford the product **3b** (29.4 mg, 50%) as a yellow solid; m.p. = 92–94 °C; *R*_f_ = 0.42 (Hex–EtOAc = 1/1, *v*/*v*); FT–IR (ATR)υ_max_/cm^−1^ 1494, 1736, 2848, 3320; ^1^H-NMR: δ 2.43–2.47 (m, 2H), 2.83–2.87 (m, 2H), 3.20 (d, *J* = 4.5 Hz, 1H), 3.57–3.63 (m, 6H), 3.74–3.76 (m, 1H), 3.80 (s, 3H), 5.64 (s, 1H), 6.67 (dd, *J* = 1.0, 7.9 Hz, 1H), 6.81 (td, *J* = 1.2, 7.5 Hz, 1H), 7.12–7.14 (m, 4H), 7.26–7.26 (m, 3H), 7.39–7.41 (m, 1H), 7.61–7.64 (m, 2H), 7.80 (dd, *J* = 1.8, 7.8 Hz, 1H); ^13^C-NMR: decomposition during data acquisition was observed; HRMS (EI): calcd. for C_31_H_31_BrN_3_O_4_^+^ 588.1492, found 582.1483.

#### 3.2.3. *Methyl 3-Benzyl-5-(3-bromophenyl)-2-morpholino-1,5,6,10b-tetrahydro-2H-2,4a-epoxybenzo[f][1,7]-naphthyridine-1-carboxylate* (**3c**)

According to GP 1, methyl (*E*)-3-(2-aminophenyl)acrylate (17.7 mg), 3-bromobenzaldehyde (14.0 μL), LiBr (4.3 mg) and 2-isocyano-1-morpholino-3-phenylpropan-1-one (26.9 mg) were reacted together in DCM (1.0 mL) to afford the product **3c** (41.2 mg, 70%) as a yellow solid; m.p. = 92–94 °C; *R*_f_ = 0.40 (Hex–EtOAc = 3/1, *v*/*v*); FT–IR (ATR)υ_max_/cm^−1^ 1494, 1736, 2848, 3320; ^1^H-NMR: δ 2.40–2.45 (m, 2H), 2.70–2.83 (m, 2H), 3.19 (d, *J* = 4.5 Hz, 1H), 3.45 (d, *J* = 4.4 Hz, 1H), 3.53–3.62 (m, 5H), 3.71 (d, *J* = 10.6 Hz, 1H), 3.81 (s, 3H), 5.06 (s, 1H), 6.67 (dd, *J* = 1.1, 8.1 Hz, 1H), 6.79 (td, *J* = 1.2, 7.5 Hz, 1H), 7.03–7.06 (m, 3H), 7.16–7.23 (m, 5H), 7.44–7.47 (m, 1H), 7.54–7.56 (m, 1H), 7.82 (s, 1H); ^13^C-NMR: δ 35.3, 47.6, 48.2, 52.5, 52.7, 58.1, 67.0, 96.9, 106.3, 115.7, 119.6, 122.0, 122.9, 126.8, 127.7(2), 128.5, 128.9, 129.5, 130.0, 131.3, 132.3, 140.1, 142.7, 170.9, 178.1; HRMS (EI): calcd. for C_31_H_31_BrN_3_O_4_^+^ 588.1498, found 588.1490.

#### 3.2.4. *Methyl 3-Benzyl-5-(4-bromophenyl)-2-morpholino-1,5,6,10b-tetrahydro-2H-2,4a-epoxybenzo[f][1,7]-naphthyridine-1-carboxylate* (**3d**)

According to GP 1, methyl (*E*)-3-(2-aminophenyl)acrylate (17.7 mg), 4-bromobenzaldehyde (14.0 μL), LiBr (4.3 mg) and 2-isocyano-1-morpholino-3-phenylpropan-1-one (26.9 mg) were reacted together in DCM (1.0 mL) to afford the product **3d** (44.1 mg, 75%) as a yellow solid; m.p. = 92–94 °C; *R*_f_ = 0.41 (Hex–EtOAc = 3/1, *v*/*v*); FT–IR (ATR)υ_max_/cm^−1^ 1494, 1736, 2848, 3320; ^1^H-NMR: δ 2.43–2.47 (m, 2H), 2.81–2.85 (m, 2H), 3.00–3.03 (m, 2H), 3.23 (d, *J* = 4.4 Hz, 1H), 3.48 (d, *J* = 4.4 Hz, 1H), 3.58–3.65 (m, 5H), 3.75–3.77 (m, 1H), 3.84 (s, 3H), 5.08 (s, 1H), 6.69 (dd, *J* = 1.0, 8.0 Hz, 1H), 6.82 (td, *J* = 1.1, 7.6 Hz, 1H), 7.02–7.04 (m, 1H), 7.06–7.11 (m, 2H), 7.20–7.21 (m, 2H), 7.27–7.34 (m, 3H), 7.51–7.53 (m, 3H); ^13^C-NMR: δ 35.3, 47.6, 48.3, 52.6, 52.7, 58.2, 67.0, 96.9, 106.2, 115.7, 119.6, 122.2, 123.0, 126.8, 127.7, 128.4, 128.9, 130.0, 131.0 (2), 135.5, 136.9, 142.8, 146.4, 170.9, 178.1; HRMS (EI): calcd. for C_31_H_31_BrN_3_O_4_^+^ 588.1498, found 588.1489.

#### 3.2.5. *Methyl 3-Benzyl-5-(2-methoxyphenyl)-2-morpholino-1,5,6,10b-tetrahydro-2H-2,4a-epoxybenzo[f][1,7]-naphthyridine-1-carboxylate* (**3e**)

According to GP 1, methyl (*E*)-3-(2-aminophenyl)acrylate (17.7 mg), 2-methoxybenzaldehyde (14.4 μL), LiBr (4.3 mg) and 2-isocyano-1-morpholino-3-phenylpropan-1-one (26.9 mg) were reacted together in DCM (1.0 mL) to afford the product **3e** (39.9 mg, 74%) as a yellow solid; m.p. = 94–96 °C; *R*_f_ = 0.51 (Hex–EtOAc = 3/1, *v*/*v*); FT–IR (ATR)υ_max_/cm^−1^ 1494, 1736, 2848, 3320; ^1^H-NMR: δ 2.51–2.55 (m, 2H), 2.91–2.95 (m, 2H), 3.23 (d, *J* = 4.5 Hz, 1H), 3.54 (d, *J* = 4.4 Hz, 1H), 3.60–3.62 (m, 4H), 3.68–3.71 (m, 1H), 3.83–3.87 (m, 7H), 5.65 (s, 1H), 6.68 (dd, *J* = 1.0, 8.0 Hz, 1H), 6.81 (td, *J* = 1.1, 7.5 Hz, 1H), 6.92–6.99 (m, 2H), 7.07–7.14 (m, 5H), 7.18–7.19 (m, 2H), 7.31–7.35 (m, 1H), 7.70 (dd, *J* = 1.7, 7.7 Hz, 1H); ^13^C-NMR: δ 35.3, 48.2, 48.4, 50.5, 52.3, 52.5, 55.9, 67.0, 97.6, 106.1, 110.7, 115.8, 119.2, 120.3, 123.3, 125.9, 126.5, 127.3, 128.3, 128.9, 129.1, 129.9, 130.4, 135.8, 143.7, 158.0, 171.0, 177.8; HRMS (EI): calcd. for C_32_H_34_N_3_O_5_^+^ 540.2498, found 540.2504.

#### 3.2.6. *Methyl 3-Benzyl-5-(3-methoxyphenyl)-2-morpholino-1,5,6,10b-tetrahydro-2H-2,4a-epoxybenzo[f][1,7]-naphthyridine-1-carboxylate* (**3f**)

According to GP 1, methyl (*E*)-3-(2-aminophenyl)acrylate (17.7 mg), 3-methoxybenzaldehyde (14.5 μL), LiBr (4.3 mg) and 2-isocyano-1-morpholino-3-phenylpropan-1-one (26.9 mg) were reacted together in DCM (1.0 mL) to afford the product **3f** (37.8 mg, 70%) as a yellow solid; m.p. = 94–96 °C; *R*_f_ = 0.53 (Hex–EtOAc = 3/1, *v*/*v*); FT–IR (ATR)υ_max_/cm^−1^ 1494, 1736, 2848, 3320; ^1^H-NMR: δ 2.42–2.46 (m, 2H), 2.80–2.85 (m, 2H), 3.20 (d, *J* = 4.5 Hz, 1H), 3.45 (d, *J* = 4.4 Hz, 1H), 3.55–3.63 (m, 5H), 3.71–3.73 (m, 1H), 3.80–3.82 (m, 3H), 3.83–3.48 (m, 4H), 5.03 (s, 1H), 6.65 (d, *J* = 7.6 Hz, 1H), 6.77 (td, *J* = 1.1, 7.6 Hz, 1H), 6.87–6.89 (m, 1H), 7.02–7.06 (m, 4H), 7.14–7.15 (m, 2H), 7.25–7.27 (m, 2H), 7.53–7.54 (m, 2H); ^13^C-NMR: δ 35.3, 47.9, 48.3, 52.6, 55.4, 58.7, 67.0, 97.2, 106.2, 113.6, 115.0, 115.7, 119.4, 121.6, 123.0, 126.7, 127.7, 128.4, 128.9, 129.0, 130.0, 135.6, 139.3, 143.0, 159.4, 171.0, 177.8; HRMS (EI): calcd. for C_32_H_34_N_3_O_5_^+^ 540.2498, found 540.2509.

#### 3.2.7. *Methyl 3-Benzyl-5-(4-methoxyphenyl)-2-morpholino-1,5,6,10b-tetrahydro-2H-2,4a-epoxybenzo[f][1,7]-naphthyridine-1-carboxylate* (**3g**)

According to GP 1, methyl (*E*)-3-(2-aminophenyl)acrylate (17.7 mg), 4-metoxibenzaldehyde (14.5 μL), LiBr (4.3 mg) and 2-isocyano-1-morpholino-3-phenylpropan-1-one (26.9 mg) were reacted together in DCM (1.0 mL) to afford the product **3g** (35.1 mg, 65%) as a yellow solid; m.p. = 94–96 °C; *R*_f_ = 0.55 (Hex–EtOAc = 3/1, *v*/*v*); FT–IR (ATR)υ_max_/cm^−1^ 1494, 1736, 2848, 3320; ^1^H-NMR: δ 2.41–2.46 (m, 2H), 2.80–2.85 (m, 2H), 3.20 (d, *J* = 4.5 Hz, 1H), 3.45 (d, *J* = 4.4 Hz, 1H), 3.53–3.63 (m, 5H), 3.71–3.75 (m, 1H), 3.80 (s, 3H), 3.83 (s, 3H), 3.86 (d, *J* = 2.0 Hz, 1H), 5.03 (s, 1H), 6.65 (dd, *J* = 1.2, 8.1 Hz, 1H), 6.77 (td, *J* = 1.2, 7.4 Hz, 1H), 6.88 (d, *J* = 8.8 Hz, 2H), 7.01–7.08 (m, 3H), 7.14–7.15 (m, 2H), 7.23–7.27 (m, 1H), 7.30–7.39 (m, 1H), 7.53 (d, *J* = 8.8 Hz, 2H); ^13^C-NMR: δ 35.1, 47.7, 48.1, 52.4, 52.5, 55.2, 57.9, 66.9, 97.2, 106.0, 113.2, 115.5, 119.1, 122.9, 126.5, 127.4, 128.2, 128.5, 128.8, 129.2, 129.8, 130.2, 130.6, 135.5, 143.0, 159.5, 170.9, 177.6; HRMS (EI): calcd. for C_32_H_34_N_3_O_5_^+^ 540.2498, found 540.2504.

#### 3.2.8. *Ethyl 3-Benzyl-2-morpholino-5-phenyl-1,5,6,11b-tetrahydro-2H-2,4a-epoxy[1,3]dioxolo[4′,5′:4,5]-benzo[1–2-f][1,7]naphthyridine-1-carboxylate* (**3h**)

According to GP 1, ethyl (*E*)-3-(6-aminobenzo[*d*][1,3]dioxol-5-yl)acrylate (13.5 mg), benzaldehyde (12.2 μL), LiBr (4.3 mg) and 2-isocyano-1-morpholino-3-phenylpropan-1-one (26.9 mg) were reacted together in DCM (1.0 mL) to afford the product **3h** (40.9 mg, 72%) as a yellow solid; m.p. = 96–98 °C; *R*_f_ = 0.62 (Hex–EtOAc = 3/1, *v*/*v*); FT–IR (ATR)υ_max_/cm^−1^ 1494, 1736, 2848, 3320; ^1^H-NMR: δ 1.34 (t, *J* = 7.1 Hz, 3H), 2.41–2.44 (m, 2H), 2.84–2.85 (m, 2H), 3.11 (d, *J* = 4.5 Hz, 1H), 3.37 (d, *J* = 4.5 Hz, 1H), 3.52–3.62 (m, 6H), 3.74 (d, *J* = 16.3 Hz, 1H), 4.19–4.30 (m, 3H), 5.01 (s, 1H), 5.85 (dd, *J* = 1.4, 4.8 Hz, 2H), 6.22 (s, 1H), 6.55 (s, 1H), 6.97–6.99 (m, 2H), 7.11–7.13 (m, 2H), 7.33–7.34 (m, 2H), 7.59–7.61 (m, 2H); ^13^C-NMR: δ 14.4, 35.4, 48.0, 48.2, 52.7, 59.0, 61.6, 67.0, 97.3, 97.5, 100.8, 106.0, 108.8, 114.9, 126.6, 128.0, 128.4, 128.9, 129.3, 135.7, 137.9, 141.4, 147.1, 170.5, 177.9; HRMS (EI): calcd. for C_33_H_34_N_3_O_6_^+^ 568.2448, found 568.2437.

### 3.3. Synthesis and Characterization of the Polysubstituted 5-Arylbenzo[f][1,7]naphthyridines ***4a**–**h***

General procedure 2 (GP 2)

The corresponding oxa-bridged 5-arylbenzo[*f*][1,7]naphthyridine (0.05 mmol, 1.0 equiv.) was placed in a round-bottomed flask equipped with a magnetic stirring bar and diluted in anhydrous acetonitrile [0.01 M] at 0 °C. Then, trifluoroacetic acid (1.0 equiv.) was dropwise added at 0 °C. Then, the mixture was stirred for 60 min at 0 °C and 240 min at room temperature. After that, an aqueous solution of sodium bicarbonate (5 mL) was added and the combined layers were placed into a corning separation flask (125 mL), where vigorous extractions using dichloromethane (3 × 5 mL) were performed. The organic phase was washed with brine, dried using anhydrous sodium sulphate and filtered over a Celite pad (1 cm). Then, the solvent was removed to dryness under vacuum. Finally, the crude residue was purified immediately by silica-gel column chromatography using a mixture of Hex–EtOAc in 3:1 (*v*/*v*) proportion as mobile phase to afford the corresponding products **4a**–**h**.

#### 3.3.1. *Methyl 3-Benzyl-2-morpholino-5-phenylbenzo[f][1,7]naphthyridine-1-carboxylate* (**4a**)

According to GP 2, methyl 3-benzyl-2-morpholino-5-phenyl-1,5,6,10*b*-tetrahydro-2*H*-2,4*a*-epoxybenzo[*f*][1,7]naphthyridine-1-carboxylate (**3a**, 25.5 mg) and TFA (3.8 μL) were reacted together in ACN (5.0 mL) to afford the product **4a** (19.1 mg, 75%) as a yellow foaming solid; m.p. = 159–161 °C; *R*_f_ = 0.65 (Hex–EtOAc = 3/1, *v*/*v*); FT–IR (ATR)υ_max_/cm^−1^ 1109, 1434, 1731, 2854; ^1^H-NMR: δ 3.04–3.35 (m, 4H), 3.74–3.86 (m, 4H), 4.18 (s, 3H), 4.44 (s, 2H), 7.25–7.32 (m, 5H), 7.40–7.46 (m, 3H), 7.65 (ddd, *J* = 1.4, 7.0, 8.5 Hz, 1H), 7.80 (ddd, *J* = 1.3, 7.0, 8.3 Hz, 1H), 7.93–7.95 (m, 2H), 8.20 (dd, *J* = 0.7, 8.5 Hz, 1H), 8.30 (dd, *J* = 0.9, 8.2 Hz, 1H); ^13^C-NMR: δ 41.4, 51.1, 53.2, 67.9, 123.6, 126.6, 127.4, 127.7, 128.6, 128.8, 129.5, 131.2, 131.4, 138.5, 138.8, 138.9 (2), 143.0, 144.6, 159.9, 161.0, 169.7; HRMS (EI): calcd. for C_31_H_28_N_3_O_3_^+^ 490.2131, found 490.2109.

#### 3.3.2. *Methyl 3-Benzyl-5-(2-bromophenyl)-2-morpholinobenzo[f][1,7]naphthyridine-1-carboxylate* (**4b**)

According to GP 2, methyl 3-benzyl-5-(2-bromophenyl)-2-morpholino-1,5,6,10*b*-tetrahydro-2*H*-2,4*a*-epoxybenzo[*f*][1,7]naphthyridine-1-carboxylate (**3b**, 29.4 mg) and TFA (3.8 μL) were reacted together in ACN (5.0 mL) to afford the product **4b** (13.2 mg, 45%) as a yellow foaming solid; m.p. = 171–173 °C; *R*_f_ = 0.50 (Hex–EtOAc = 3/1, *v*/*v*); FT–IR (ATR)υ_max_/cm^−1^ 1109, 1434, 1731, 2854; ^1^H-NMR: δ 2.95–3.03 (m, 2H), 3.23–3.32 (m, 2H), 3.62–3.64 (m, 2H), 3.76–3.78 (m, 2H), 4.16 (s, 3H), 4.29 (s, 2H), 7.14–7.16 (m, 5H), 7.32–7.35 (m, 1H), 7.41–7.43 (m, 2H), 7.65–7.68 (m, 2H), 7.78–7.81 (m, 1H), 8.18–8.20 (m, 1H), 8.27–8.29 (m, 1H); ^13^C-NMR: δ 41.1, 51.0, 53.1, 67.8, 126.3, 126.9, 127.7, 128.1, 129.3, 129.5 (2), 130.7, 131.1, 132.3, 138.1, 138.4, 138.7, 140.7, 143.2, 144.2, 161.0, 161.5, 169.5; HRMS (EI): calcd. for C_32_H_27_BrN_3_O_5_^+^ 568.1236, found 568.1234.

#### 3.3.3. *Methyl 3-Benzyl-5-(3-bromophenyl)-2-morpholinobenzo[f][1,7]naphthyridine-1-carboxylate* (**4c**)

According to GP 2, methyl 3-benzyl-5-(3-bromophenyl)-2-morpholino-1,5,6,10*b*-tetrahydro-2*H*-2,4*a*-epoxybenzo[*f*][1,7]naphthyridine-1-carboxylate (**3c**, 29.4 mg) and TFA (3.8 μL) were reacted together in ACN (5.0 mL) to afford the product **4c** (16.2 mg, 55%) as a yellow foaming solid; m.p. = 147–149 °C; *R*_f_ = 0.51 (Hex–EtOAc = 3/1, *v*/*v*); FT–IR (ATR) υ_max_/cm^−1^ 1109, 1434, 1731, 2854; ^1^H-NMR: δ 3.00–3.32 (m, 4H), 3.60–3.64 (m, 2H), 3.76–3.78 (m, 2H), 4.16 (s, 3H), 4.42 (s, 2H), 7.21–7.23 (m, 4H), 7.28–7.31 (m, 2H), 7.57 (ddd, *J* = 1.0, 2.1, 8.0 Hz, 1H), 7.62–7.66 (m, 1H), 7.77–7.80 (m, 2H), 8.17 (ddd, *J* = 0.5, 1.2, 8.5 Hz, 1H), 8.19–8.20 (m, 1H), 8.27 (ddd, *J* = 0.5, 1.4, 8.2 Hz, 1H); ^13^C-NMR: δ 41.2, 51.0, 53.2, 67.8, 124.4, 126.5, 127.6, 128.5, 129.0, 129.3, 129.6, 130.2, 131.1, 131.6, 133.8, 138.5, 138.8, 140.5, 143.1, 144.3, 158.2, 161.1, 169.5; HRMS (EI): calcd. for C_32_H_27_BrN_3_O_5_^+^ 568.1236, found 568.1224.

#### 3.3.4. *Methyl 3-Benzyl-5-(4-bromophenyl)-2-morpholinobenzo[f][1,7]naphthyridine-1-carboxylate* (**4d**)

According to GP 2, methyl 3-benzyl-5-(4-bromophenyl)-2-morpholino-1,5,6,10*b*-tetrahydro-2*H*-2,4*a*-epoxybenzo[*f*][1,7]naphthyridine-1-carboxylate (**3d**, 29.4 mg) and TFA (3.8 μL) were reacted together in ACN (5.0 mL) to afford the product **4d** (7.4 mg, 25%) as a yellow foaming solid; m.p. = 186–188 °C; *R*_f_ = 0.50 (Hex–EtOAc = 3/1, *v*/*v*); FT–IR (ATR)υ_max_/cm^−1^ 1109, 1434, 1731, 2854; ^1^H-NMR: δ 3.06–3.33 (m, 4H), 3.71–3.82 (m, 4H), 4.16 (s, 3H), 4.41 (s, 2H), 6.75–6.78 (m, 1H), 7.18–7.24 (m, 5H), 7.46–7.49 (m, 2H), 7.61–7.65 (m, 1H), 7.77–7.79 (m, 2H), 8.17 (ddd, *J* = 0.5, 1.3, 8.5 Hz, 1H), 8.24 (ddd, *J* = 0.5, 1.4, 8.2 Hz, 1H); ^13^C-NMR: δ 41.4, 51.1, 53.3, 67.9, 125.6, 126.3, 126.7, 127.6, 128.4, 128.7, 129.1, 129.6, 130.8, 131.5, 133.1, 137.4, 138.7, 143.1, 144.6, 161.2, 169.7; HRMS (EI): calcd. for C_32_H_27_BrN_3_O_5_^+^ 568.1236, found 568.1231.

#### 3.3.5. *3-Benzyl-5-(2-methoxyphenyl)-2-morpholinobenzo[f][1,7]naphthyridine-1-carboxylate* (**4e**)

According to GP 2, methyl 3-benzyl-5-(2-methoxyphenyl)-2-morpholino-1,5,6,10*b*-tetrahydro-2*H*-2,4*a*-epoxybenzo[*f*][1,7]naphthyridine-1-carboxylate (**3e**, 27.0 mg) and TFA (3.8 μL) were reacted together in ACN (5.0 mL) to afford the product **4e** (13.5 mg, 50%) as a yellow foaming solid; m.p. = 94–96 °C; *R*_f_ = 0.45 (Hex–EtOAc = 3/1, *v*/*v*); FT–IR (ATR)υ_max_/cm^−1^ 1109, 1434, 1731, 2854; ^1^H-NMR: δ 2.77–2.82 (m, 1H), 3.00–3.09 (m, 3H), 3.53–3.60 (m, 1H), 3.62–3.69 (m, 2H), 3.74–3.79 (m, 1H), 3.90 (s, 3H), 3.93 (s, 3H), 4.24 (d, *J* = 3.9 Hz, 2H), 5,94 (s, 1H), 6.54–6.58 (m, 2H), 6.68–6.72 (m, 2H), 6.87 (dd, *J* = 0.9, 8.2 Hz, 1H), 7.00–7.03 (m, 1H), 7.12–7.16 (m, 5H), 7.41 (dd, *J* = 1.2, 8.0 Hz, 1H); ^13^C-NMR: δ 40.5, 52.8, 55.6, 56.1, 67.9, 68.1, 110.6, 116.3, 118.4, 119.2, 120.4, 125.4, 120.5, 124.0, 125.4, 126.2, 128.3, 128.7, 129.9, 138.9, 139.5, 141.7, 144.9, 152.4, 157.1, 158.0, 169.8; HRMS (EI): calcd. for C_32_H_30_N_3_O_4_^+^ 520.2236, found 520.2229.

#### 3.3.6. *3-Benzyl-5-(3-Methoxyphenyl)-2-morpholinobenzo[f][1,7]naphthyridine-1-carboxylate* (**4f**)

According to GP 2, methyl 3-benzyl-5-(3-methoxyphenyl)-2-morpholino-1,5,6,10*b*-tetrahydro-2*H*-2,4a-epoxybenzo[*f*][1,7]naphthyridine-1-carboxylate (**3f**, 27.0 mg) and TFA (3.8 μL) were reacted together in ACN (5.0 mL) to afford the product **4f** (12.4 mg, 46%) as a yellow foaming solid; m.p. = 139–141 °C; *R*_f_ = 0.47 (Hex–EtOAc = 3/1, *v*/*v*); FT–IR (ATR)υ_max_/cm^−1^ 1109, 1434, 1731, 2854; ^1^H-NMR: δ 3.62–3.64 (m, 2H), 3.77–378 (m, 2H), 3.89 (s, 3H), 4.16 (s, 3H), 4.44 (s, 2H), 6.89–6.90 (m, 2H), 7.23–7.25 (m, 2H), 7.29–7.34 (m, 3H), 7.57–7.61 (m, 1H), 7.74–7.77 (m, 1H), 7.94–7.95 (m, 2H), 8.15 (ddd, *J* = 0.5, 1.3, 8.4 Hz, 1H), 8.24 (ddd, *J* = 0.5, 1.4, 8.2 Hz, 1H); ^13^C-NMR: δ 41.5, 51.1, 53.3, 67.9, 72.4, 113.2, 121.7, 123.6, 124.5, 126.6, 127.1, 128.6, 129.5, 129.6, 131.1, 133.1, 138.9, 139.0, 142.9, 144.7, 159.1, 160.4, 160.9, 169.8; HRMS (EI): calcd. for C_32_H_30_N_3_O_4_^+^ 520.2236, found 520.2226.

#### 3.3.7. *3-Benzyl-5-(4-methoxyphenyl)-2-morpholinobenzo[f][1,7]naphthyridine-1-carboxylate* (**4g**)

According to GP 2, methyl 3-benzyl-5-(4-methoxyphenyl)-2-morpholino-1,5,6,10*b*-tetrahydro-2*H*-2,4*a*-epoxybenzo[*f*][1,7]naphthyridine-1-carboxylate (**3g**, 27.0 mg) and TFA (3.8 μL) were reacted together in ACN (5.0 mL) to afford the product **4g** (7.0 mg, 26%) as a yellow foaming solid; m.p. = 146–148 °C; *R*_f_ = 0.46 (Hex–EtOAc = 3/1, *v*/*v*); FT–IR (ATR)υ_max_/cm^−1^ 1109, 1434, 1731, 2854; ^1^H-NMR: δ 3.01–3.34 (m, 4H), 3.76–3.79 (m, 4H), 3.89 (s, 3H), 4.16 (s, 3H), 4.44 (s, 2H), 6.89–6.91 (m, 2H), 7.23–7.25 (m, 2H), 7.29–7.34 (m, 3H), 7.59 (ddd, *J* = 1.4, 7.0, 8.5 Hz, 1H), 7.76 (ddd, *J* = 1.3, 7.0, 8.3 Hz, 1H), 7.94–7.95 (m, 2H), 8.15 (ddd, *J* = 0.5, 1.3, 8.4 Hz, 1H), 8.24 (ddd, *J* = 0.5, 1.4, 8.2 Hz, 1H); ^13^C-NMR: δ 41.5, 51.1, 53.1, 67.9, 72.4, 113.2, 121.7, 123.6, 124.5, 126.6, 127.1, 128.6, 129.5, 129.6, 131.1, 133.1, 138.9, 142.9, 144.7, 160.4, 160.9, 169.8; HRMS (EI): calcd. for C_32_H_30_N_3_O_4_^+^ 520.2236, found 520.2229.

#### 3.3.8. *Ethyl 3-Benzyl-2-morpholino-5-phenyl-[1,3]dioxolo[4′,5′:4,5]benzo[1,2-f][1,7]naphthyridine-1-carboxylate* (**4h**)

According to GP 2, ethyl 3-benzyl-2-morpholino-5-phenyl-1,5,6,11*b*-tetrahydro-2*H*-2,4*a*-epoxy[1,3]dioxolo[4′,5′:4,5]benzo[1,2-*f*][1,7]naphthyridine-1-carboxylate (**3h**, 28.4 mg) and TFA (3.8 μL) were reacted together in ACN (5.0 mL) to afford the product **4h** (11.4 mg, 40%) as a yellow foaming solid; m.p. = 209–211 °C; *R*_f_ = 0.64 (Hex–EtOAc = 3/1, *v*/*v*); FT–IR (ATR)υ_max_/cm^−1^ 1109, 1434, 1731, 2854; ^1^H-NMR: δ 1.49 (t, *J* = 7.2 Hz, 3H), 2.94–3.07 (m, 2H), 3.28–3.42 (m, 2H), 3.71–3.84 (m, 4H), 4.39 (s, 2H), 4.64 (q, *J* = 7.2 Hz, 2H), 6.16 (s, 2H), 7.25–7.26 (m, 5H), 7.36–7.40 (m, 3H), 7.59–7.60 (m, 2H), 7.86–7.88 (m, 2H); ^13^C-NMR: δ 14.2, 29.9, 41.4, 51.1, 62.7, 67.9, 101.1, 102.2, 108.7, 117.6, 126.5, 127.7, 128.6 (2), 128.7, 129.6, 131.4, 138.1, 138.9, 142.6, 148.5, 149.8, 157.7, 160.3, 169.4; HRMS (EI): calcd. for C_33_H_30_N_3_O_5_^+^ 548.2185, found 548.2176.

## 4. Conclusions

The synthesis or isolation of benzo[*f*][1,7]naphthyridines with a substituted aromatic ring in the C-5 position has not been published before. However, a replacement of the solvent (DCM by PhMe), a decrease in the amount of LiBr (0.05 instead of 1 equiv.) to activate the involved Schiff-bases, and the use of MW-irradiation as a heat source allowed the synthesis in good yields of the oxa-bridged 5-aryl benzo[*f*][1,7]naphthyridines as a mixtures of separable or inseparable diasteroisomers, which were used immediately for a further aromatization process under mild-acidic conditions toward eight new 5-aryl benzo[*f*][1,7]naphthyridines. The methodology described in this work means an improvement of our previous reports to access to the aromatic version of the polyheterocycles saving resources like time by using MW as heat source. 

## Supplementary Materials

The following are available online: spectra of the products **3a**–**h** and **4a**–**h**. Figures S1–S77.
