# Supplementary material for: Synthesis of New 5-Aryl-benzo[f][1,7]naphthyridines via a Cascade Process (Ugi-3CR/Intramolecular Aza-Diels-Alder Cycloaddition)/Aromatization
Source: Molecules. 2018 Aug 14;23(8):2029. doi: 10.3390/molecules23082029 (PMC6222401; doi:10.3390/molecules23082029)
Supplement: Supplementary file 1 [file molecules-23-02029-s001.pdf]

SUPPLEMENTARY MATERIAL

Synthesis of new 5-aryl-benzo[*f*][1,7]naphthyridines via a cascade process (Ugi-3CR / intramolecular aza Diels-Alder cycloaddition) / aromatization

Óscar Vázquez-Vera [1], Daniel Segura-Olvera [2], Mónica A. Rincón-Guevara [3], Atilano Gutiérrez-Carrillo [2], Miguel A. García-Sánchez [2], Ilich A. Ibarra [4], Leticia Lomas-Romero [2,*], Alejandro Islas-Jácome [2,*], and Eduardo González-Zamora [2,*]

[1] Unidad Profesional Interdisciplinaria de Biotecnología, Instituto Politécnico Nacional, Av. Acueducto de Guadalupe S/N, Barr. La Laguna Ticomán, C.P. 07340, Gustavo A Madero, Ciudad de México; vaveos@yahoo.com.mx (O.V.-V.)

[2] Departamento de Química, Universidad Autónoma Metropolitana-Iztapalapa, San Rafael Atlixco 186, Col. Vicentina, C.P. 09340, Iztapalapa, Ciudad de México; mudo-2@hotmail.com (D.S.-O.); agrmn@xanum.uam.mx (A.G.-C.); mags@xanum.uam.mx (M.A.G.-S.)

[3] Departamento de Biotecnología, Universidad Autónoma Metropolitana-Iztapalapa, San Rafael Atlixco 186, Col. Vicentina, C.P. 09340, Iztapalapa, Ciudad de México; monicarinconguevara@gmail.com (M.A.R.-G.)

[4] Laboratorio de Fisicoquímica y Reactividad de Superficies, Instituto de Investigaciones en Materiales, Universidad Nacional Autónoma de México, Circuito Exterior S/N, Ciudad Universitaria, C.P. 04510, Coyoacán, Ciudad de México; argel@unam.mx (I.A.I.)

* Correspondence: llr@xanum.uam.mx (L.L.-R.); aij@xanum.uam.mx (A.I.-J.); egz@xanum.uam.mx (E.G.-Z.); Tel.: +52-55-5804-4913 (L.L.-R.; A.I.-J.; E.G.-Z.)

CONTENTS

1H and 13C NMR spectra of the products **3a-h** **S1-S9**

1H and 13C NMR spectra of the products **4a-h** **S10-S17**

2D-NMR spectra of the products **3a, 3c-h** **S18-S28**

2D-NMR spectra of the products **4a-h** **S28-S40**

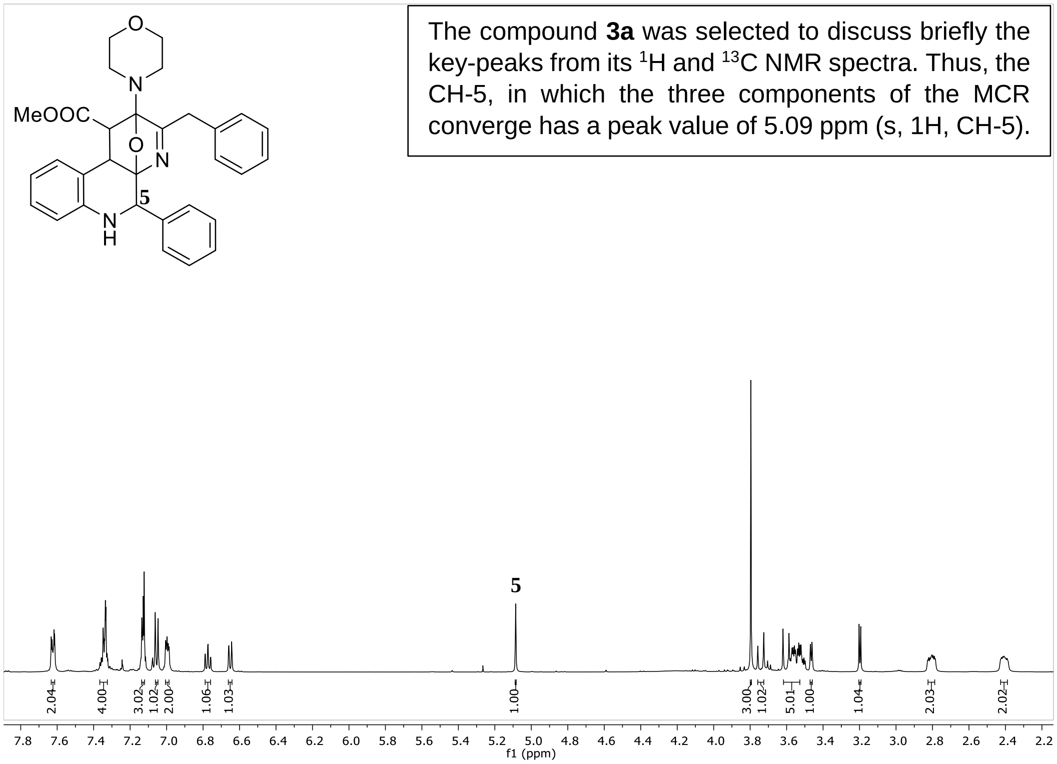

Figure S1. ^{1}H NMR spectrum of the product **3a**

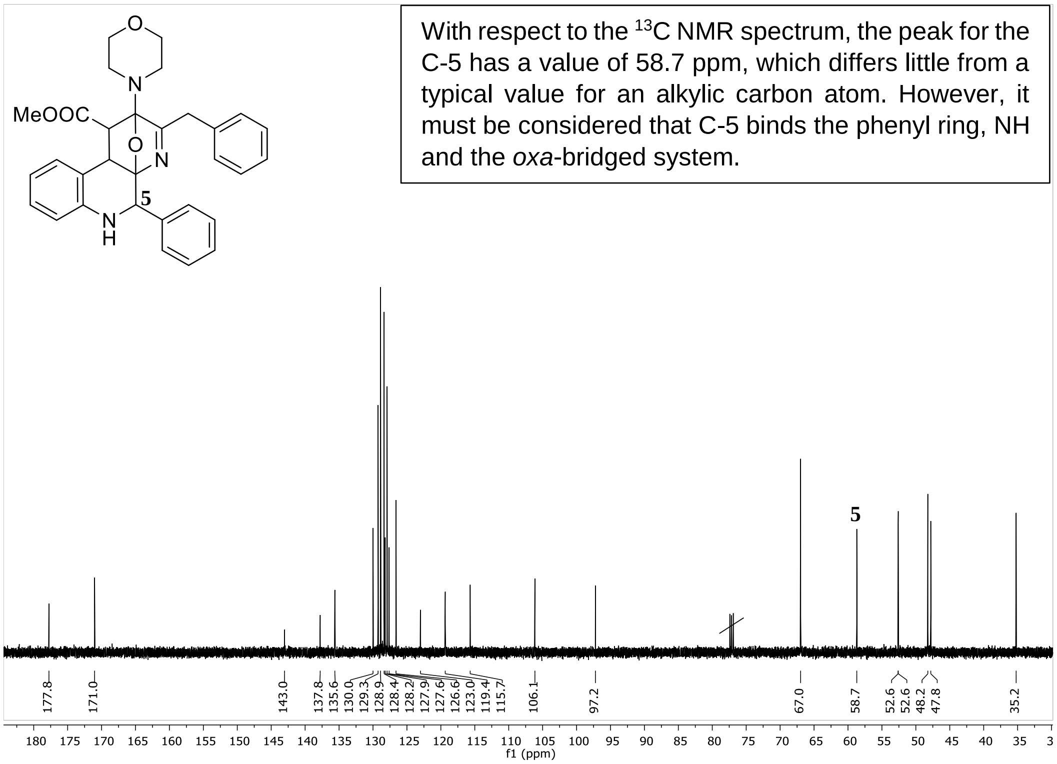

Figure S2. ^{13}C NMR spectrum of the product **3a**

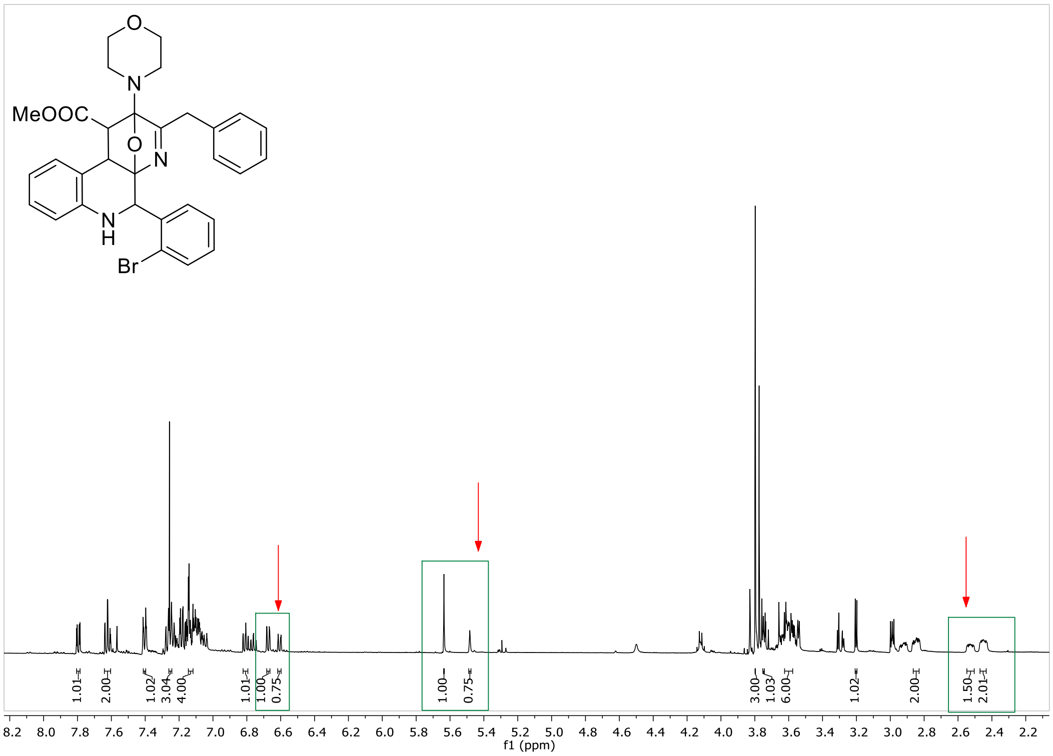

Figure S3. ^{1}H NMR spectrum of the product **3b** (mixture of inseparable diasteroisomers r = 4:3)

Mass Spectrum SmartFormula Report

Analysis Info

Acquisition Date 7/6/2018 6:20:19 PM

Analysis Name D:\Data\Eduardo Gonzalez\201807006_EGZ_Br_5.d
Method tune_low_27032017_LC.m
Sample Name 201807006_EGZ_Br_5
Comment

Operator CBS_UAM_I
Instrument micrOTOF 213750.00410

Acquisition Parameter

Source Type	ESI	Ion Polarity	Positive	Set Nebulizer	0.5 Bar
Focus	Active	Set Capillary	4500 V	Set Dry Heater	160 °C
Scan Begin	50 m/z	Set End Plate Offset	-500 V	Set Dry Gas	4.0 l/min
Scan End	1000 m/z	Set Charging Voltage	0 V	Set Divert Valve	Source
		Set Corona	0 nA	Set APCI Heater	0 °C

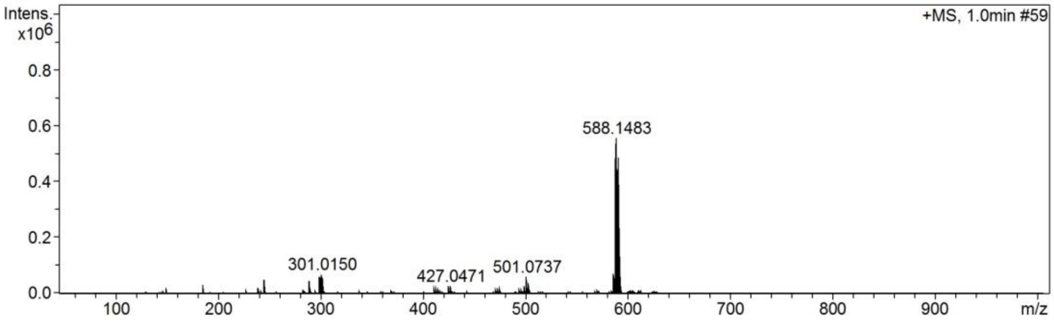

Meas. m/z	#	Ion Formula	m/z	err [ppm]	mSigma	# Sigma	Score	rdb	e¯ Conf	N-Rule
588.1483	1	C31H31BrN3O4	588.1492	-1.6	52.7	2	100.00	17.5	even	ok

Figure S4. HRMS spectrum of the product **3b**

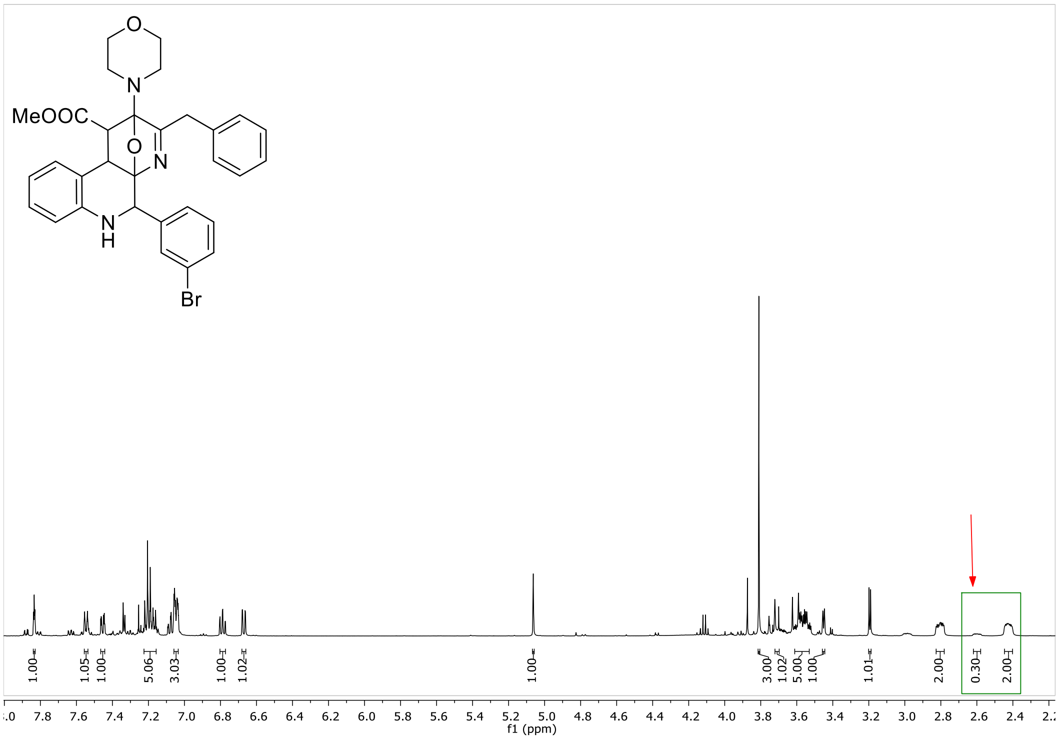

Figure S5. ^{1}H NMR spectrum of the product **3c** (mixture of inseparable diasteroisomers r = 20:3)

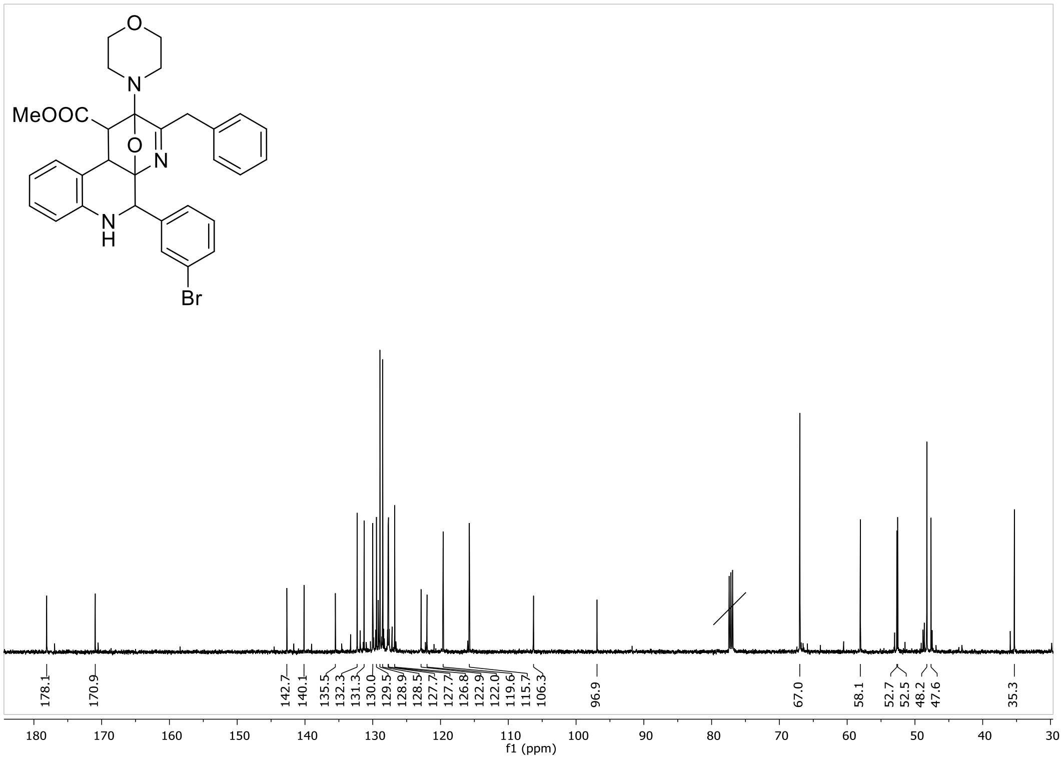

Figure S6. ^{13}C NMR spectrum of the product **3c**

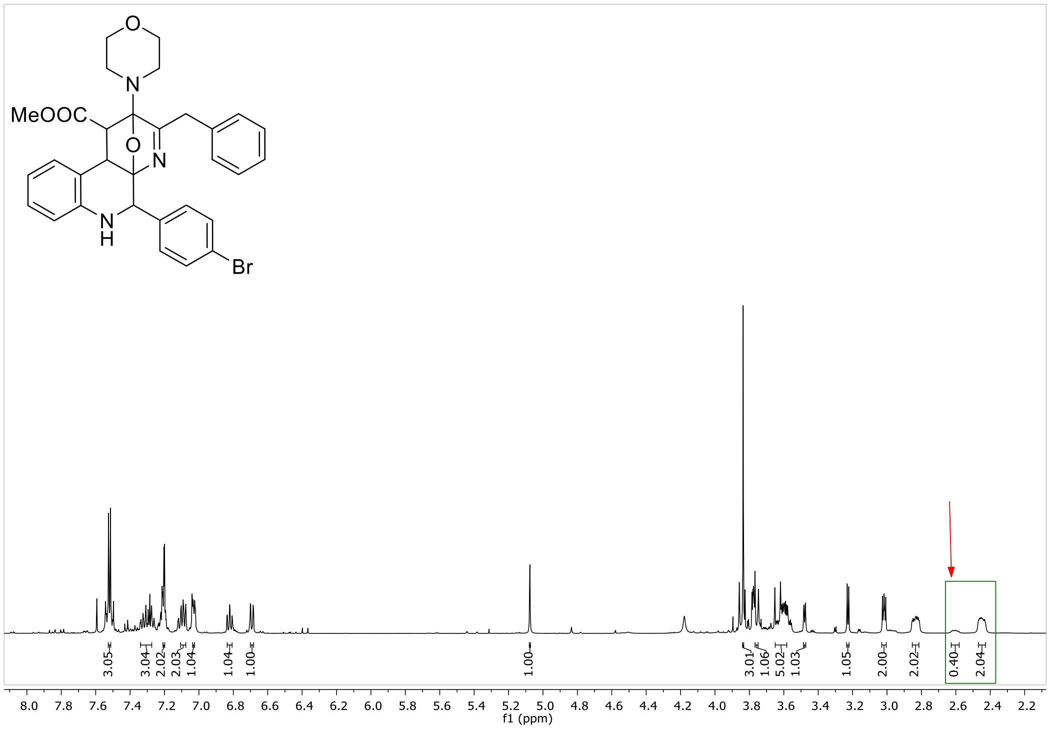

Figure S7. ^{1}H NMR spectrum of the product **3d** (mixture of inseparable diasteroisomers r = 5:1)

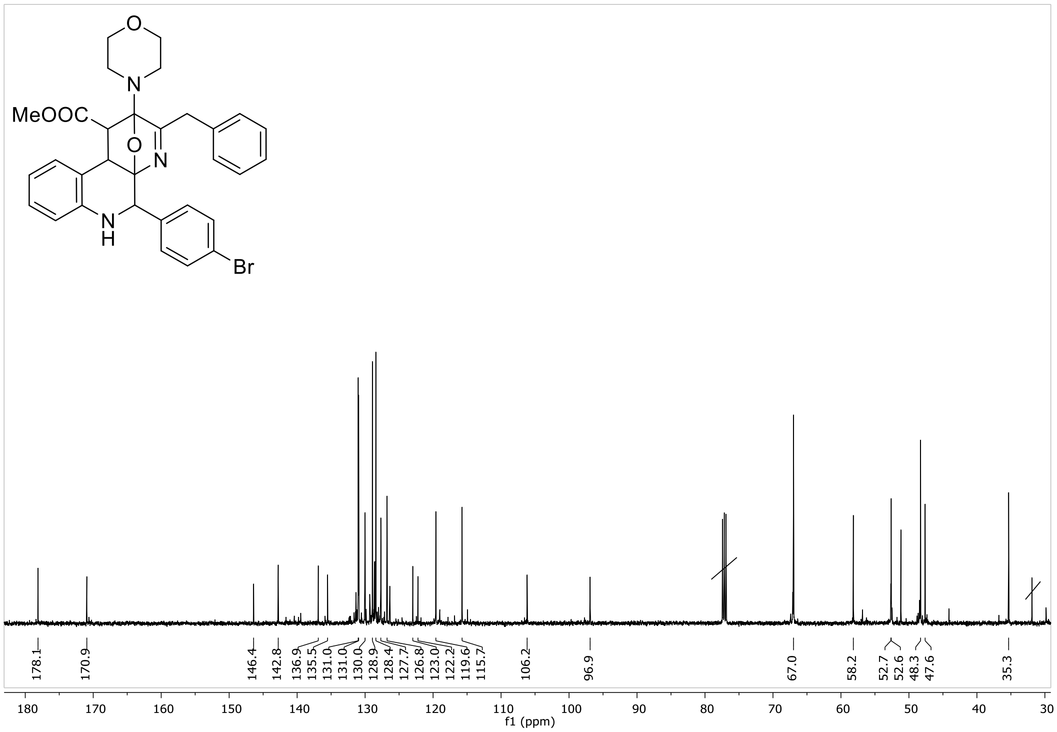

Figure S8. ^{13}C NMR spectrum of the product **3d**

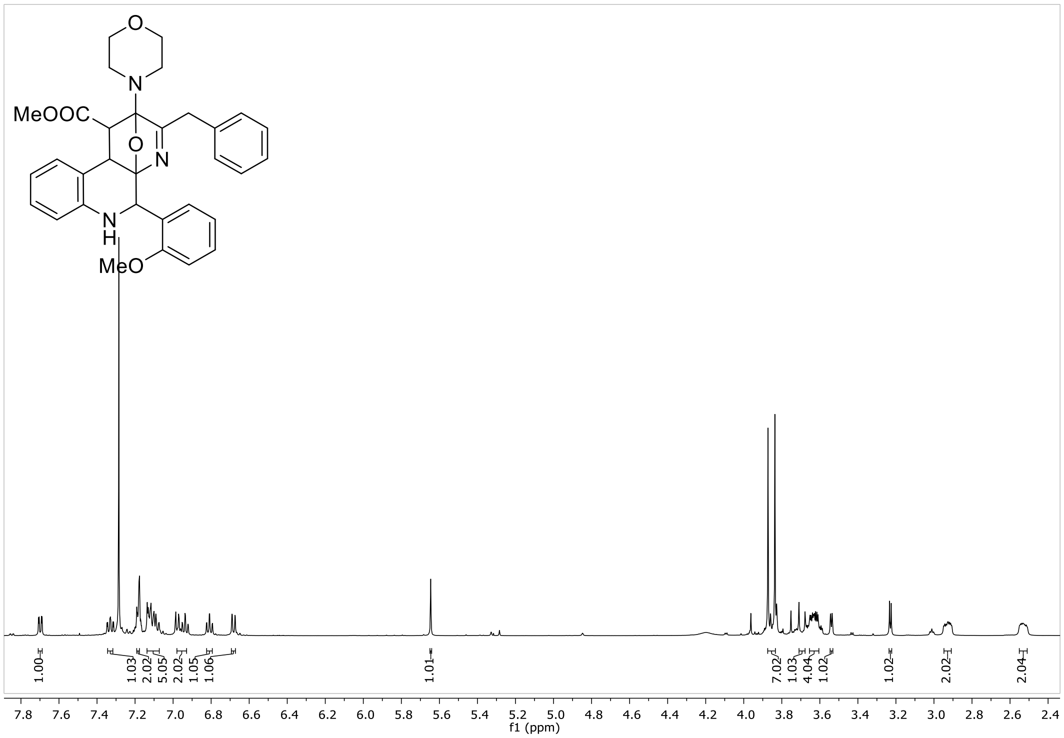

Figure S9. ^{1}H NMR spectrum of the product **3e**

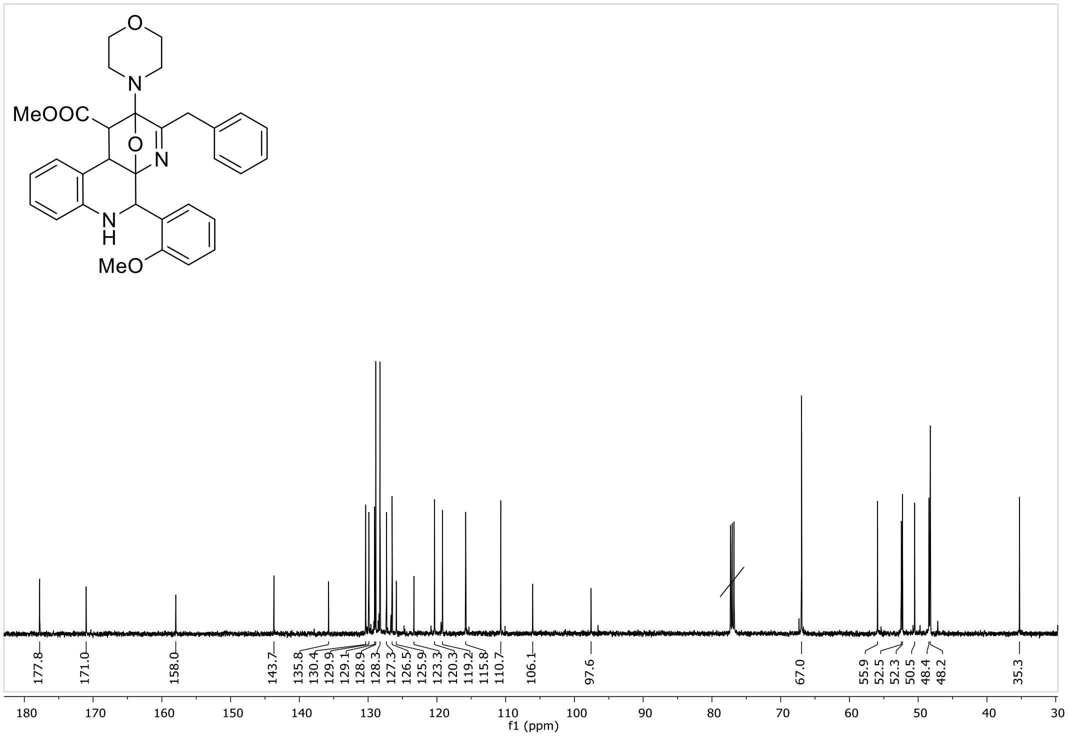

Figure S10. ^{13}C NMR spectrum of the product **3e**

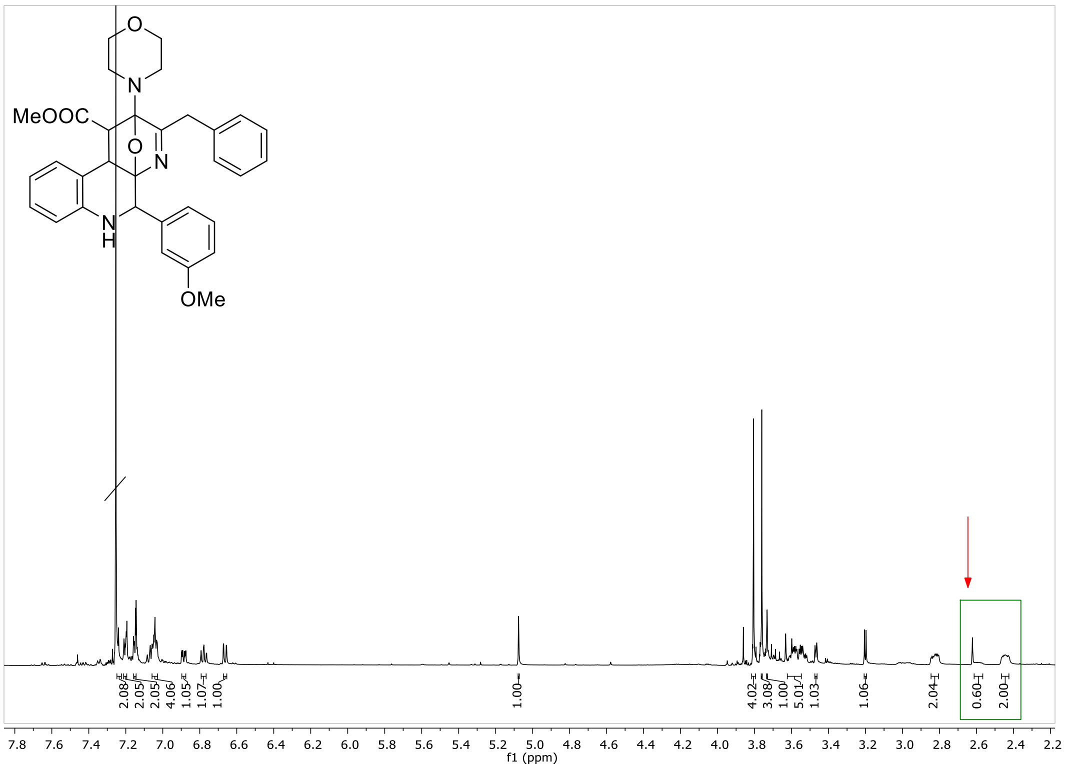

Figure S11. ^{1}H NMR spectrum of the product **3f** (mixture of inseparable diasteroisomers r = 10:3)

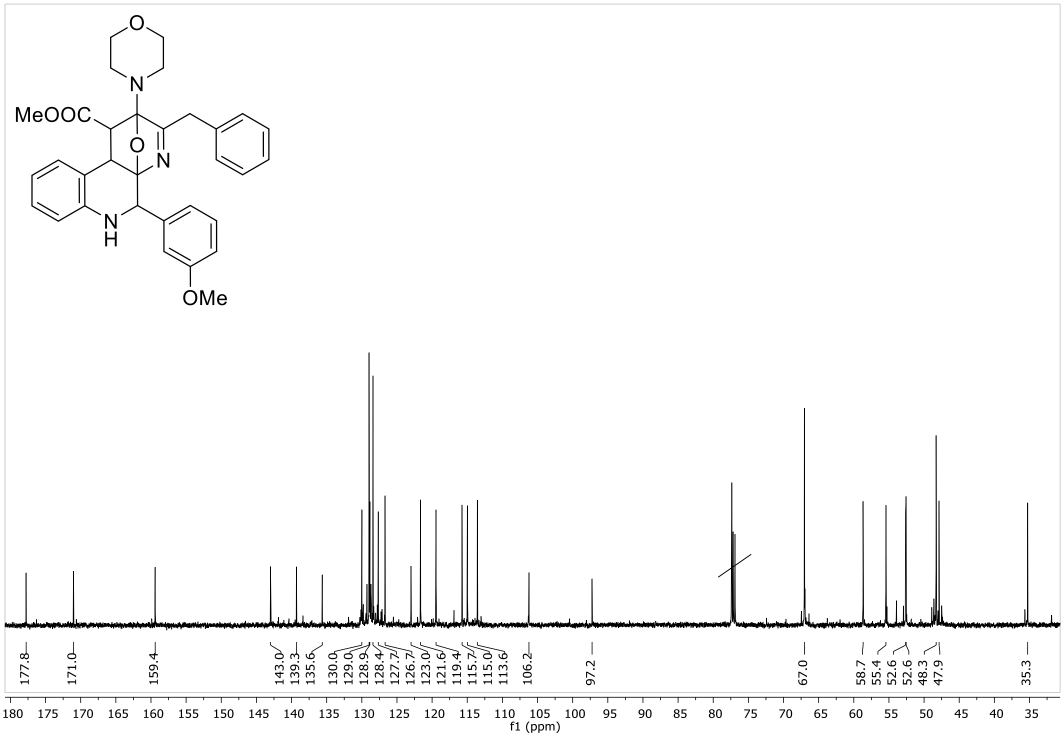

Figure S12. ^{13}C NMR spectrum of the product **3f**

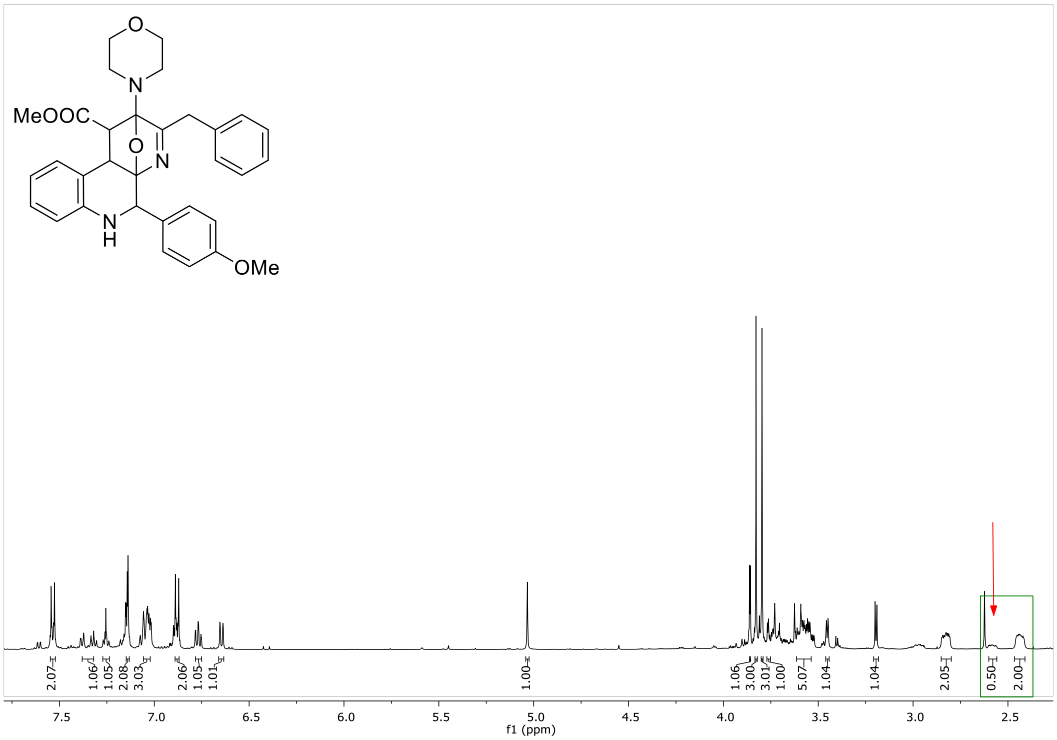

Figure S13. ^{1}H NMR spectrum of the product **3g** (mixture of inseparable diasteroisomers r = 4:1)

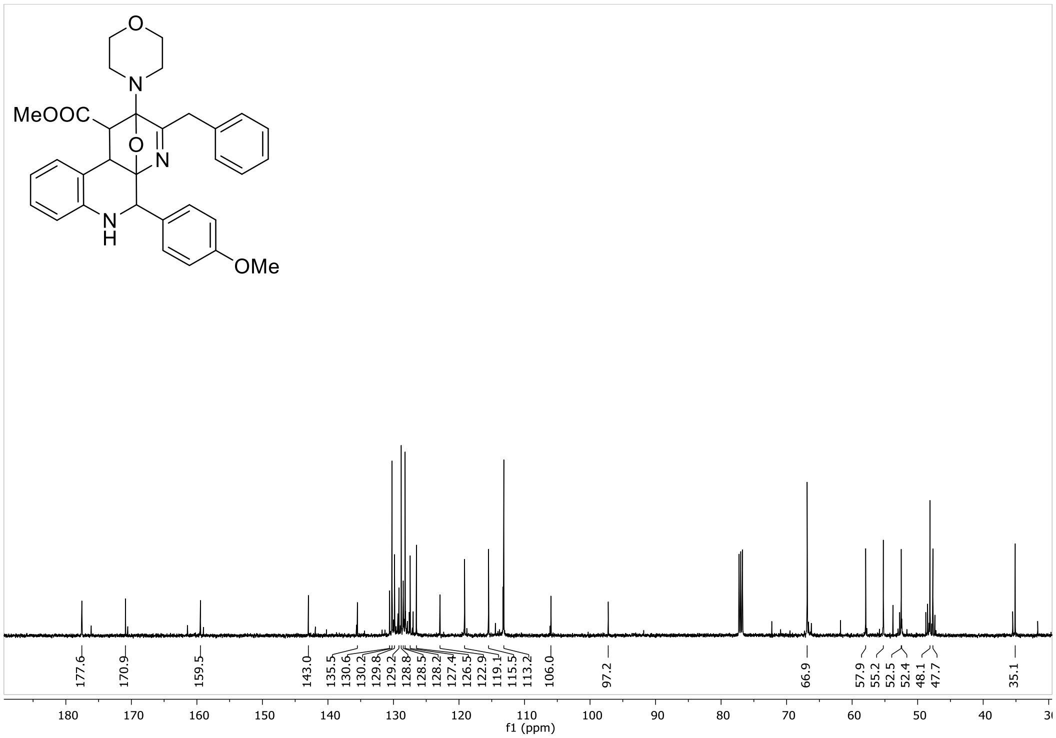

Figure S14. ^{13}C NMR spectrum of the product **3g**

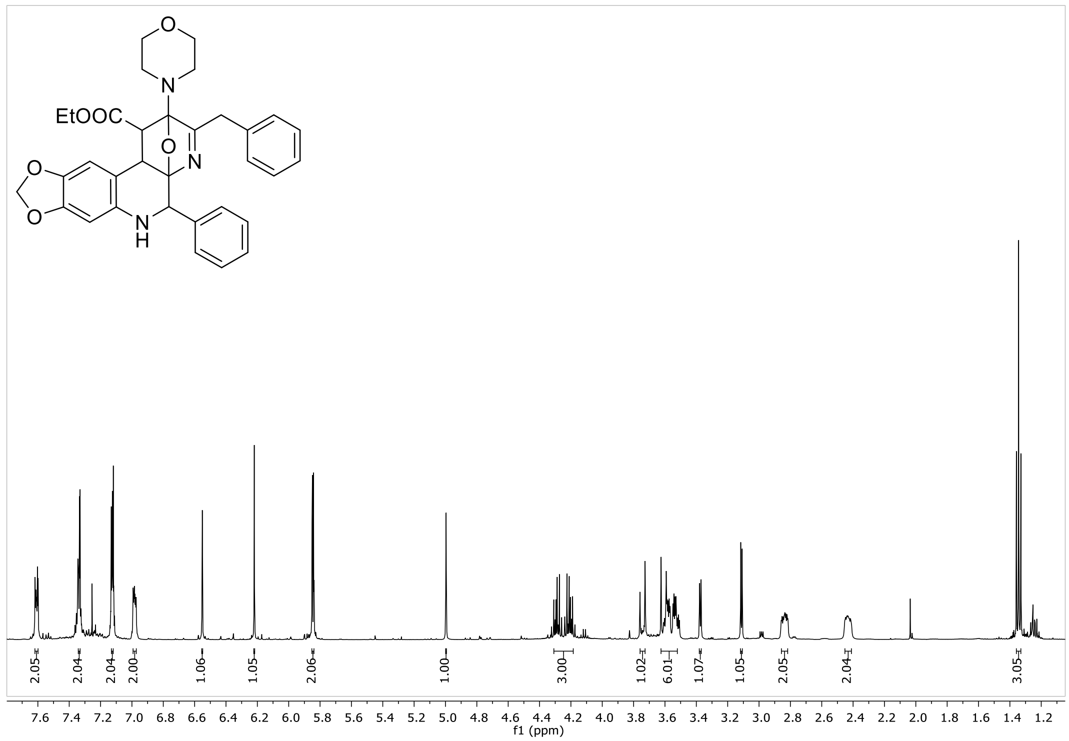

Figure S15. ^{1}H NMR spectrum of the product **3h**

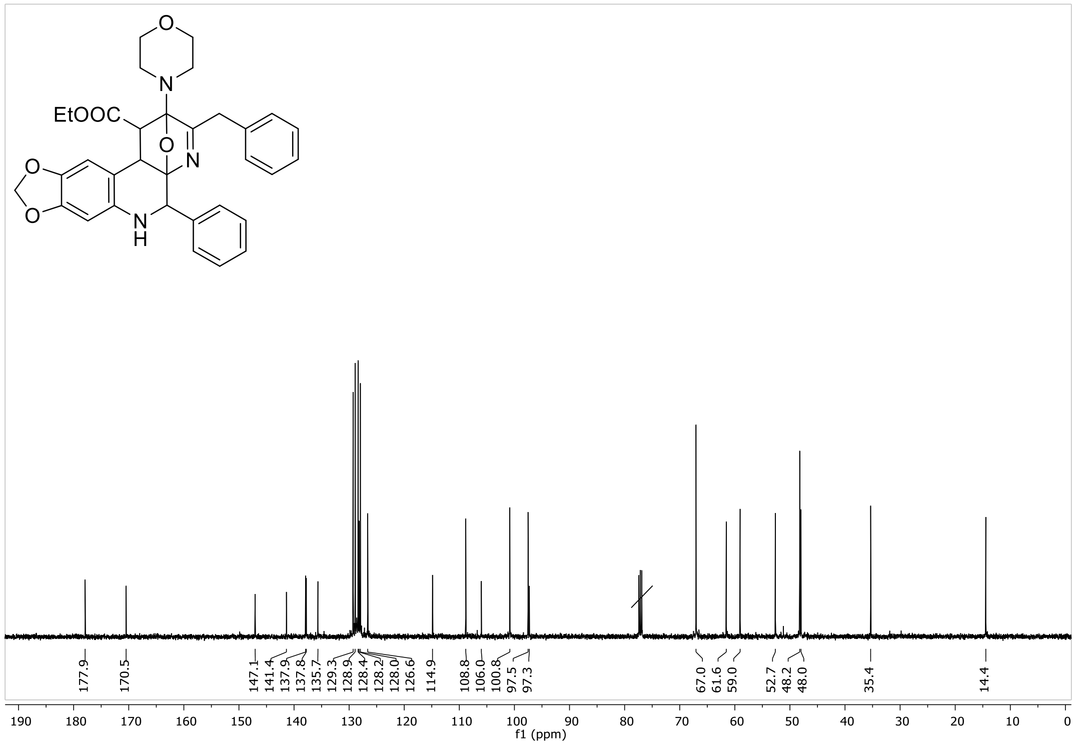

Figure S16. ^{13}C NMR spectrum of the product **3h**

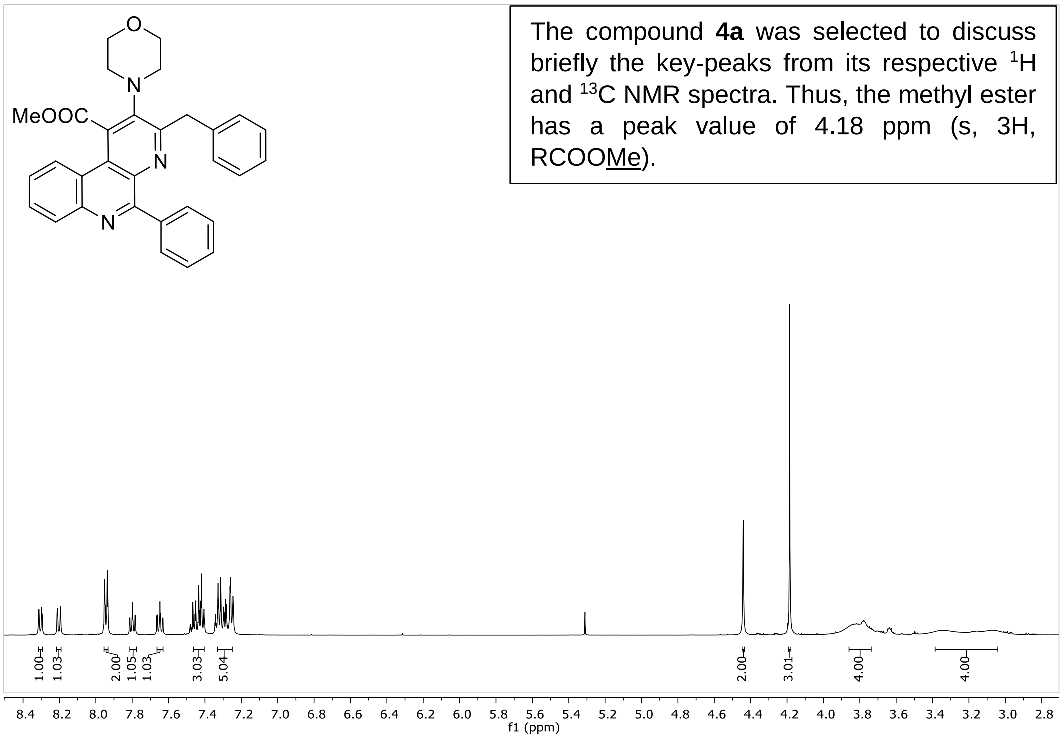

The compound **4a** was selected to discuss briefly the key-peaks from its respective ^{1}H and ^{13}C NMR spectra. Thus, the methyl ester has a peak value of 4.18 ppm (s, 3H, RCOOMe).

Figure S17. ^{1}H NMR spectrum of the product **4a**

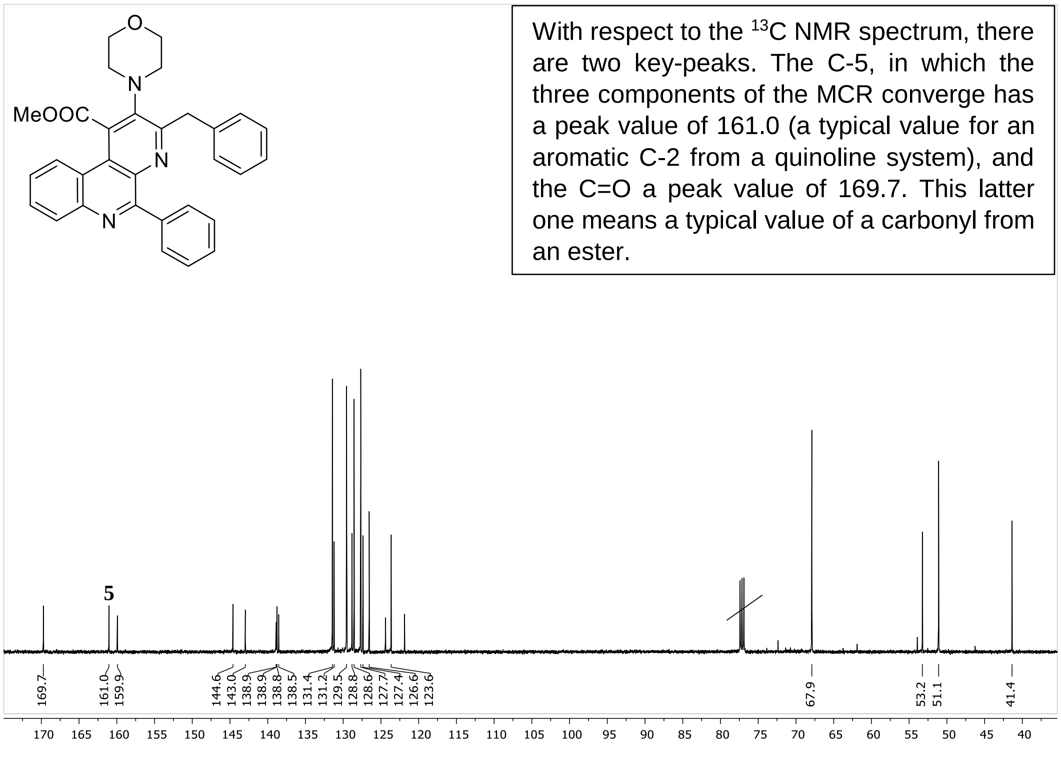

With respect to the ^{13}C NMR spectrum, there are two key-peaks. The C-5, in which the three components of the MCR converge has a peak value of 161.0 (a typical value for an aromatic C-2 from a quinoline system), and the C=O a peak value of 169.7. This latter one means a typical value of a carbonyl from an ester.

Figure S18. ^{13}C NMR spectrum of the product **4a**

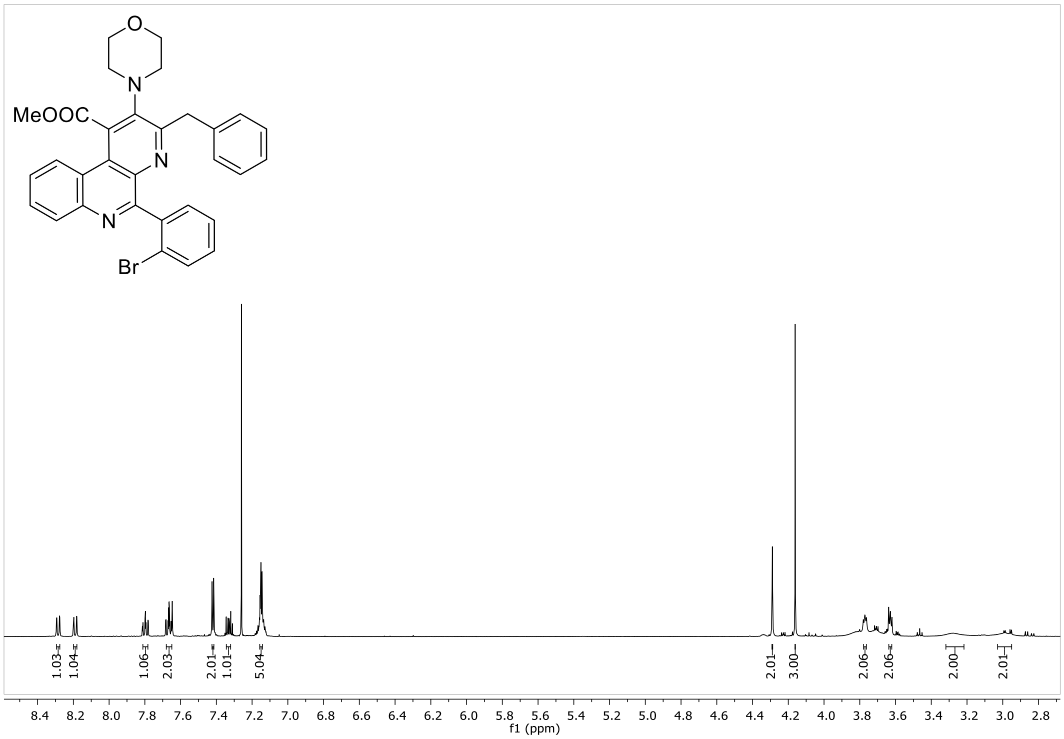

Figure S19. ^{1}H NMR spectrum of the product **4b**

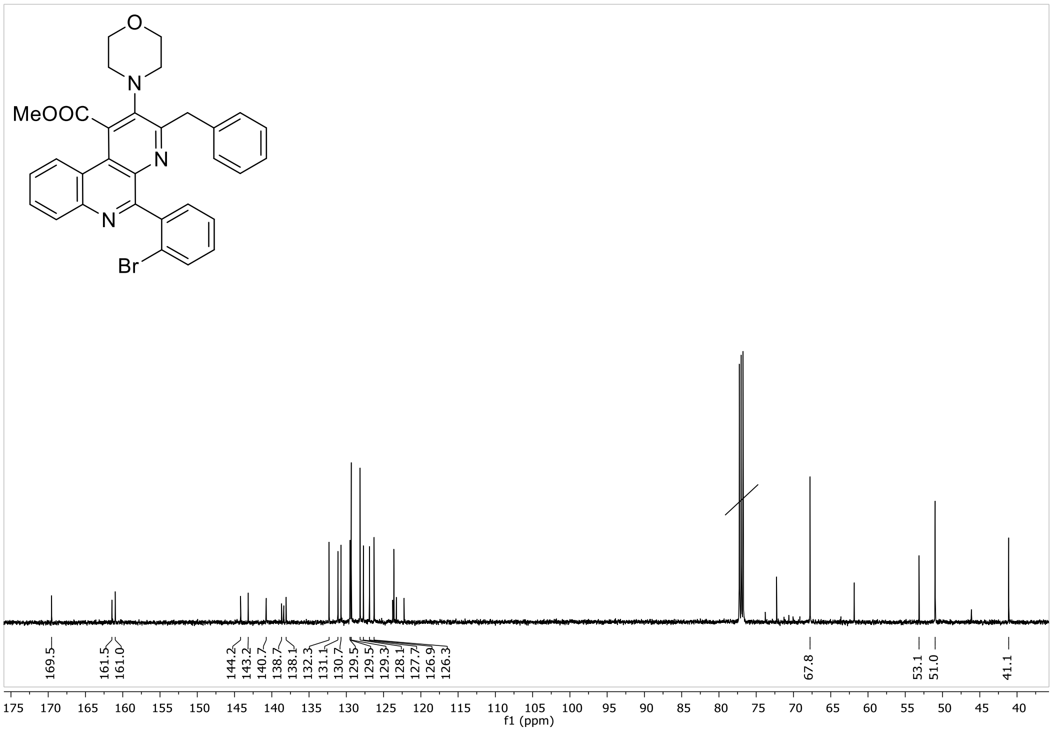

Figure S20. ^{13}C NMR spectrum of the product **4b**

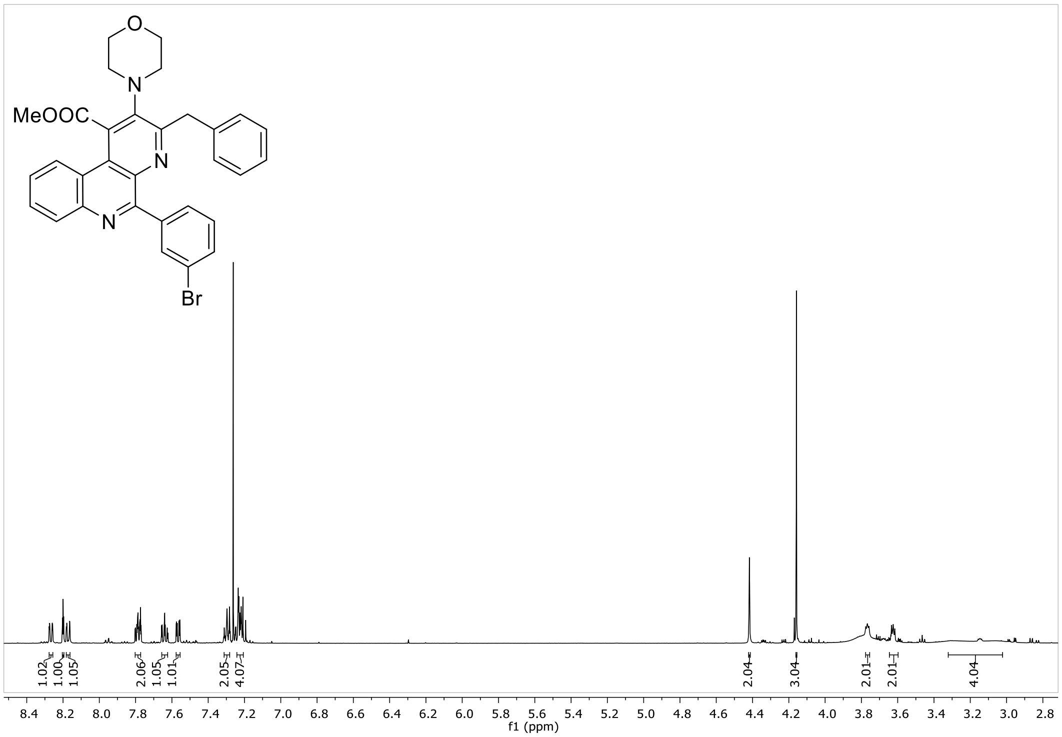

Figure S21. ^{1}H NMR spectrum of the product **4c**

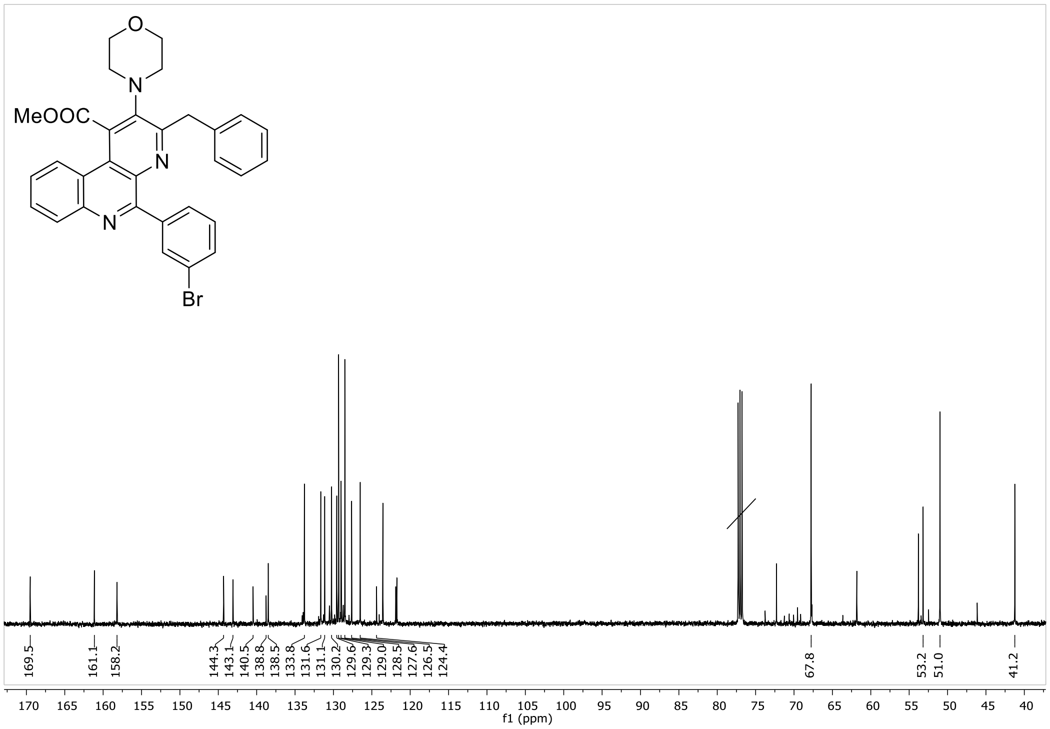

Figure S22. ^{13}C NMR spectrum of the product **4c**

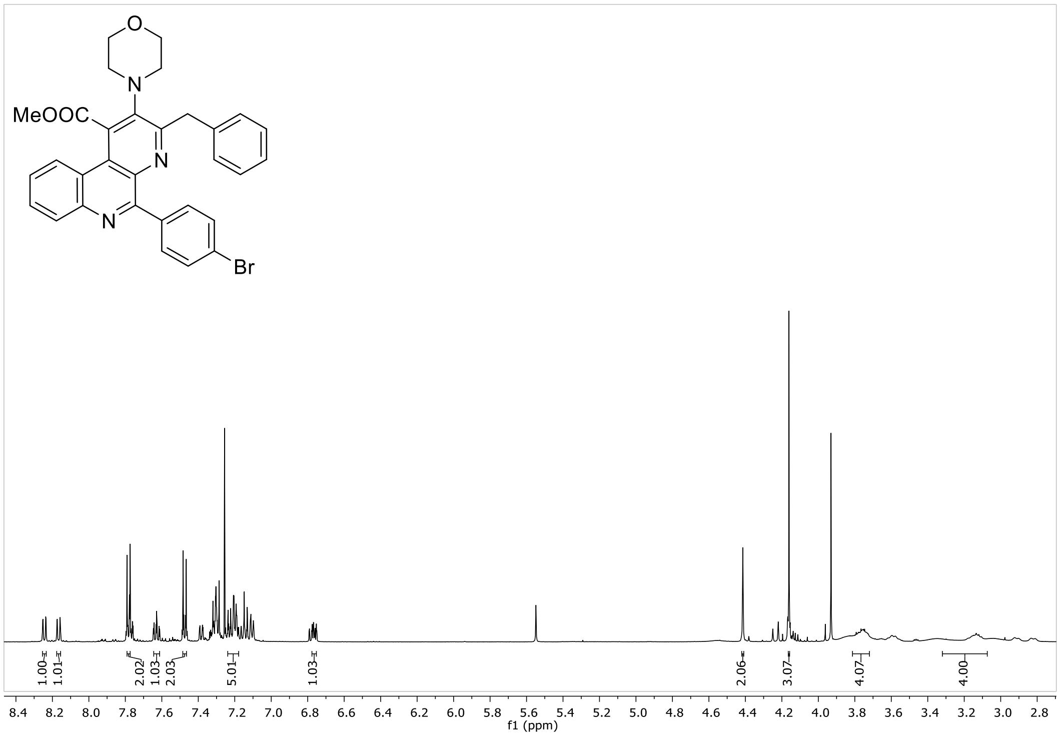

Figure S23. ^{1}H NMR spectrum of the product **4d**

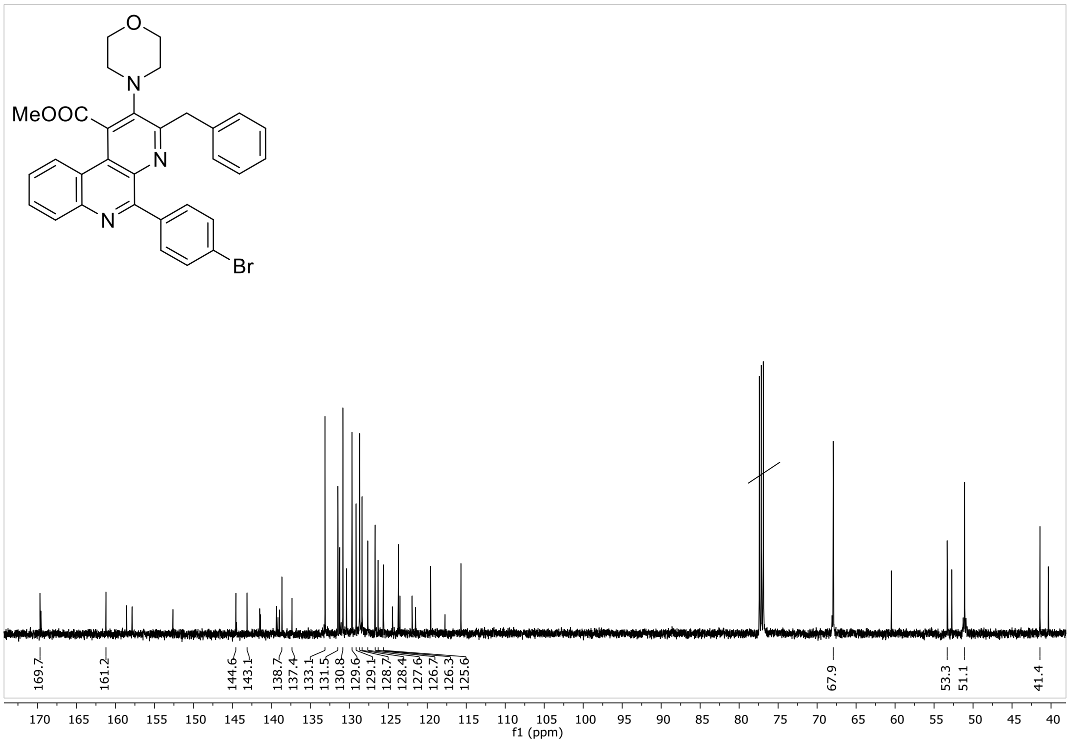

Figure S24. ^{13}C NMR spectrum of the product **4d**

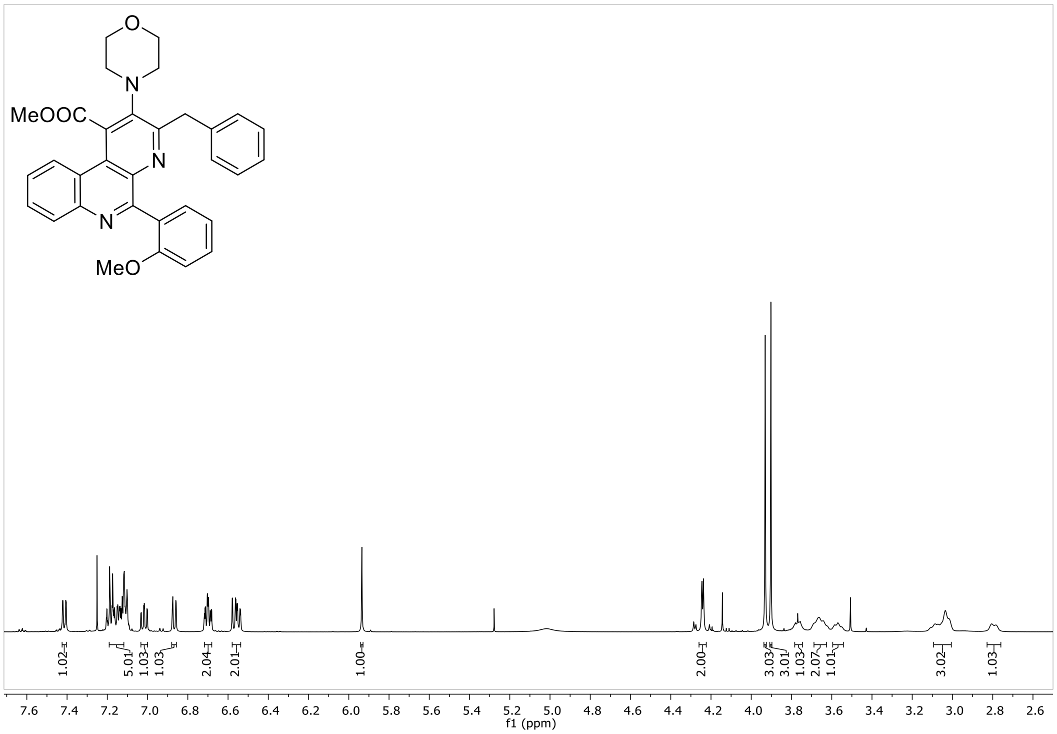

Figure S25. ^{1}H NMR spectrum of the product **4e**

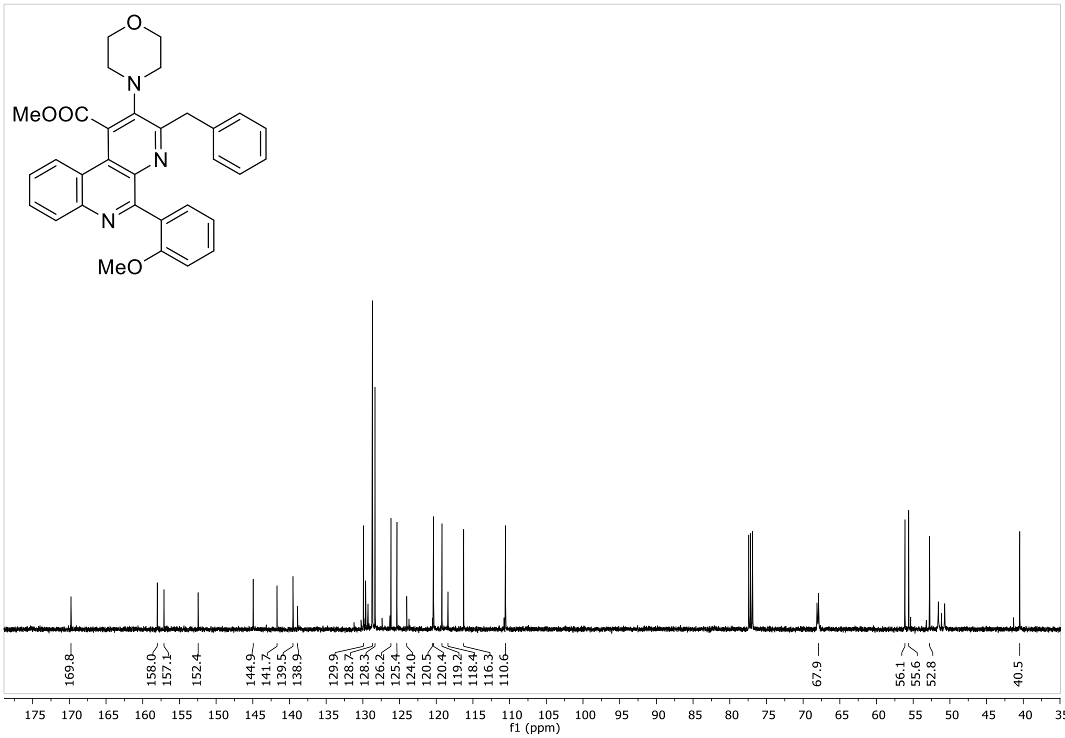

Figure S26. ^{13}C NMR spectrum of the product **4e**

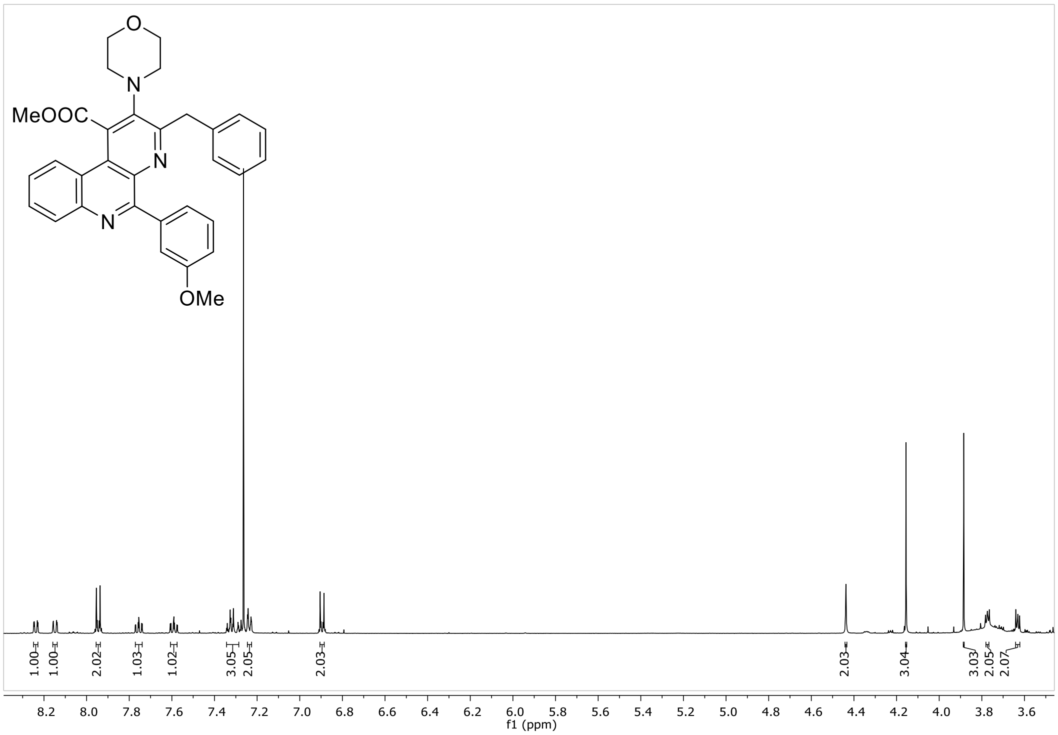

Figure S27. ^{1}H NMR spectrum of the product **4f**

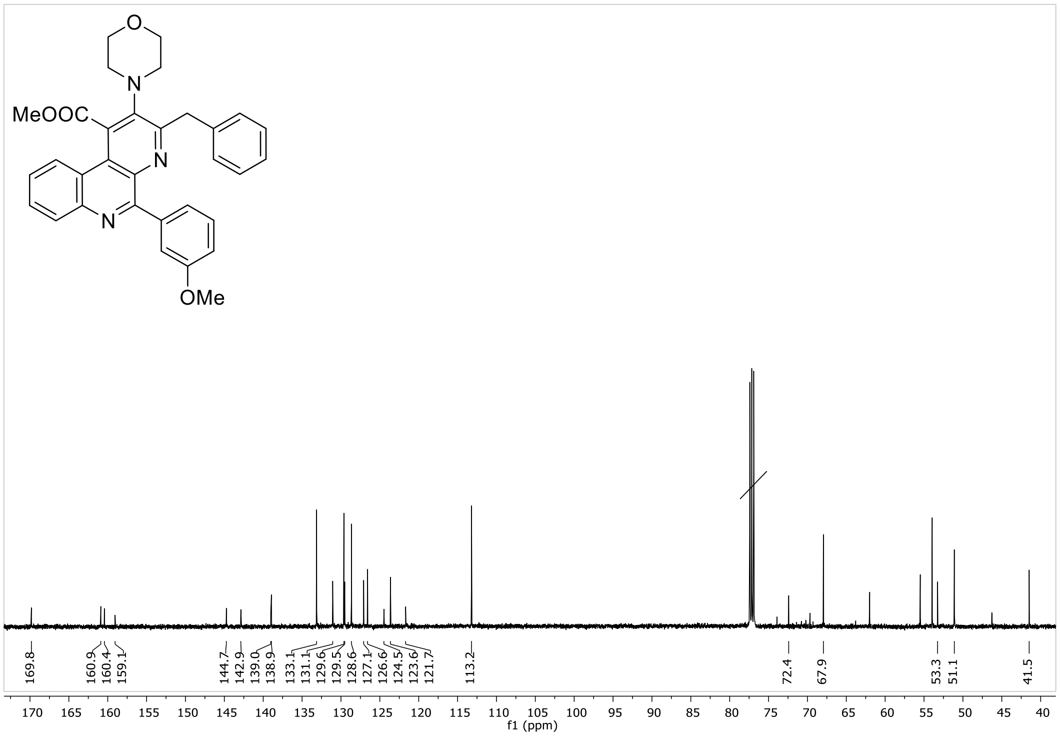

Figure S28. ^{13}C NMR spectrum of the product **4f**

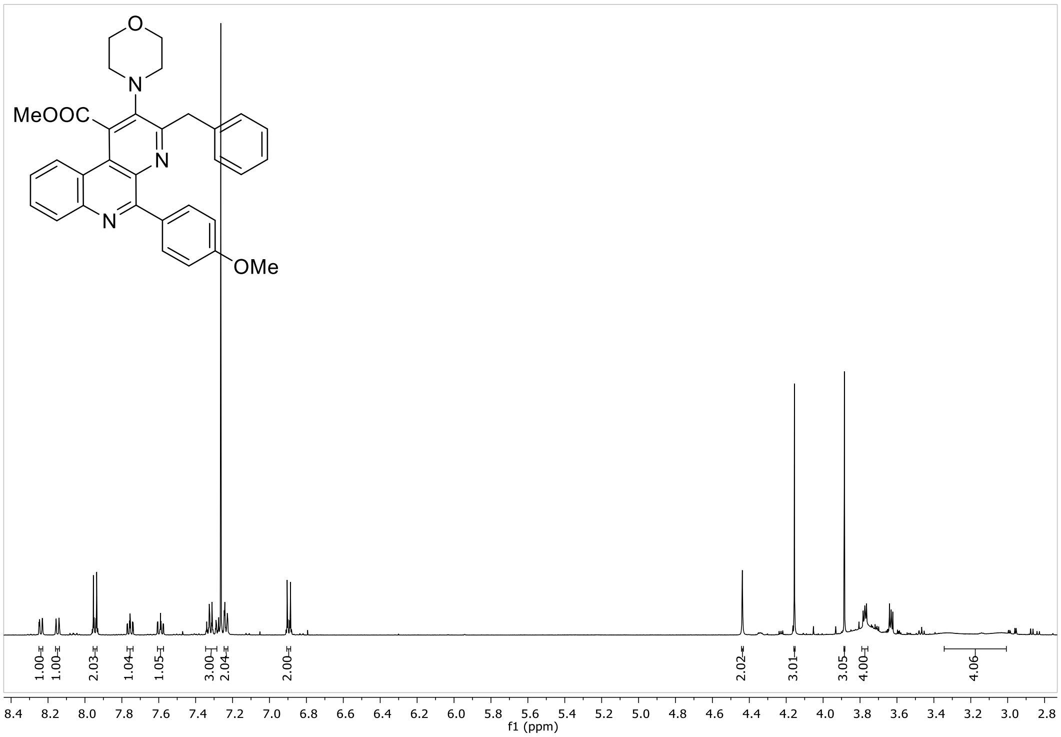

Figure S29. ^{1}H NMR spectrum of the product **4g**

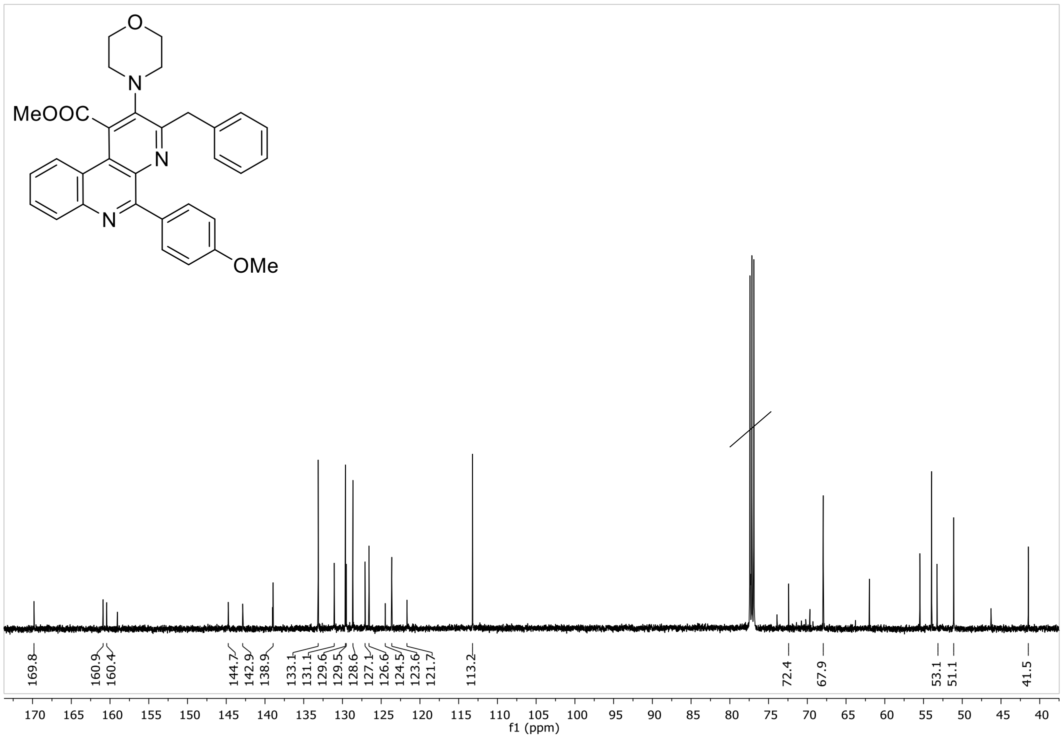

Figure S30. ^{13}C NMR spectrum of the product **4g**

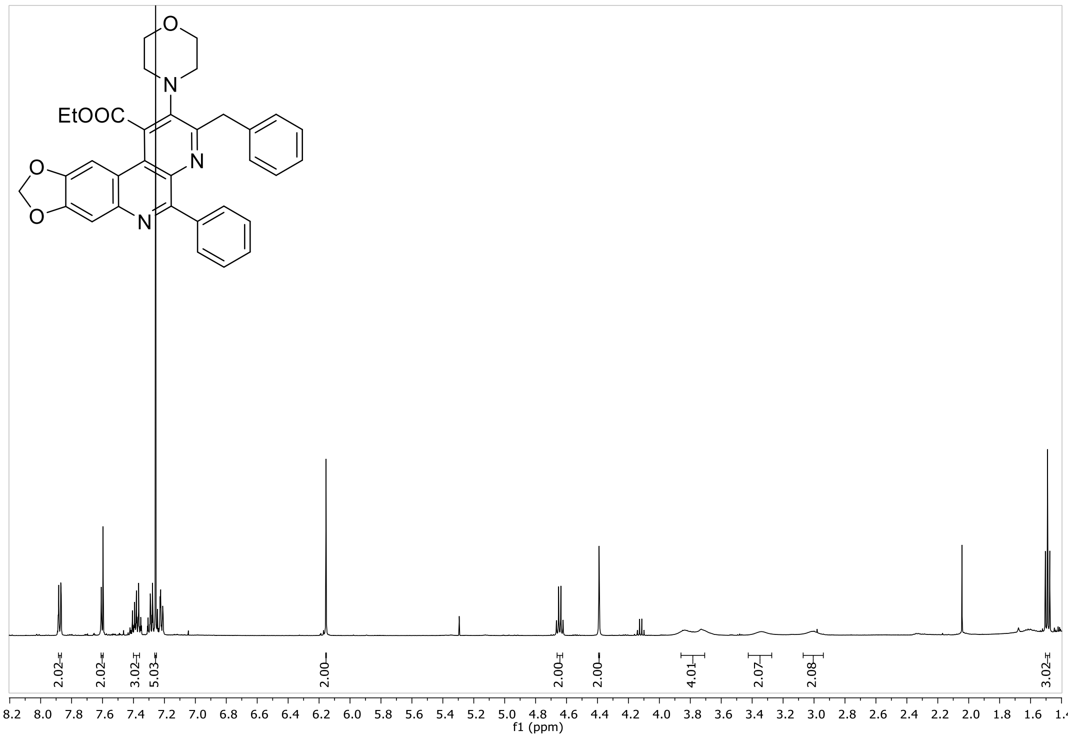

Figure S31. ^{1}H NMR spectrum of the product **4h**

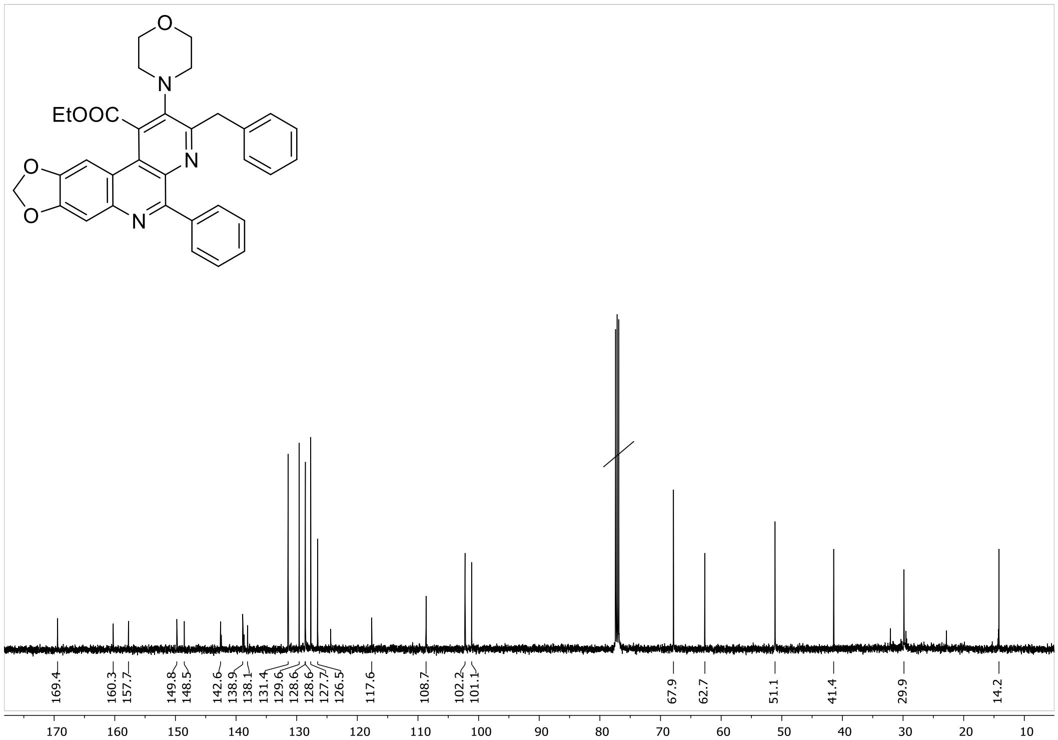

Figure S32. ^{13}C NMR spectrum of the product **4h**

2D-NMR spectra for the products **3a, 3c–h**

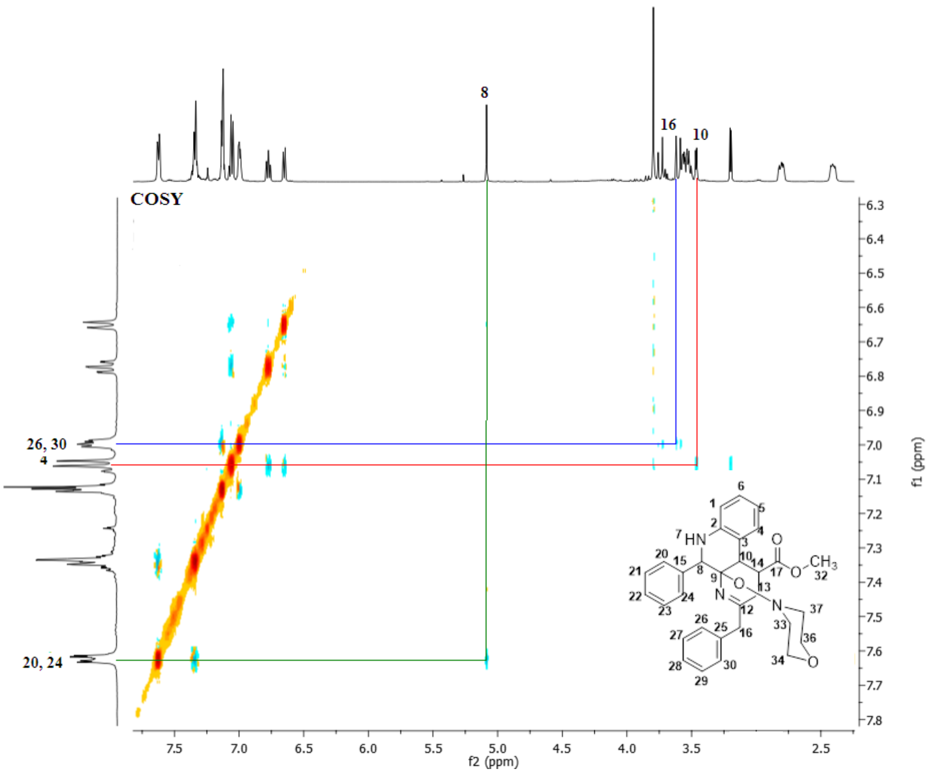

Figure S33. COSY spectrum of the product **3a**

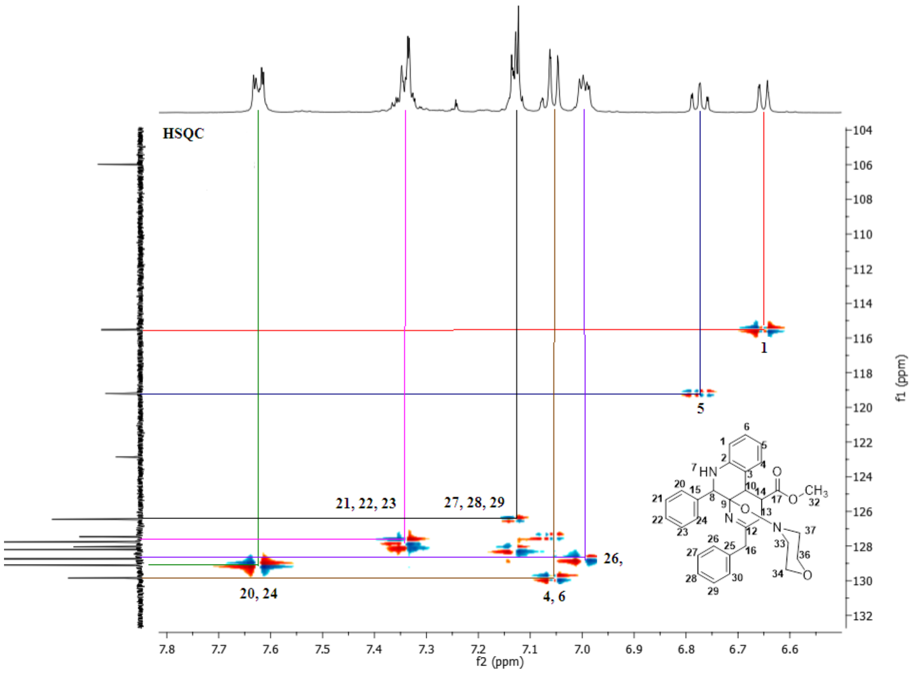

Figure S34. HSQC spectrum of the product **3a**

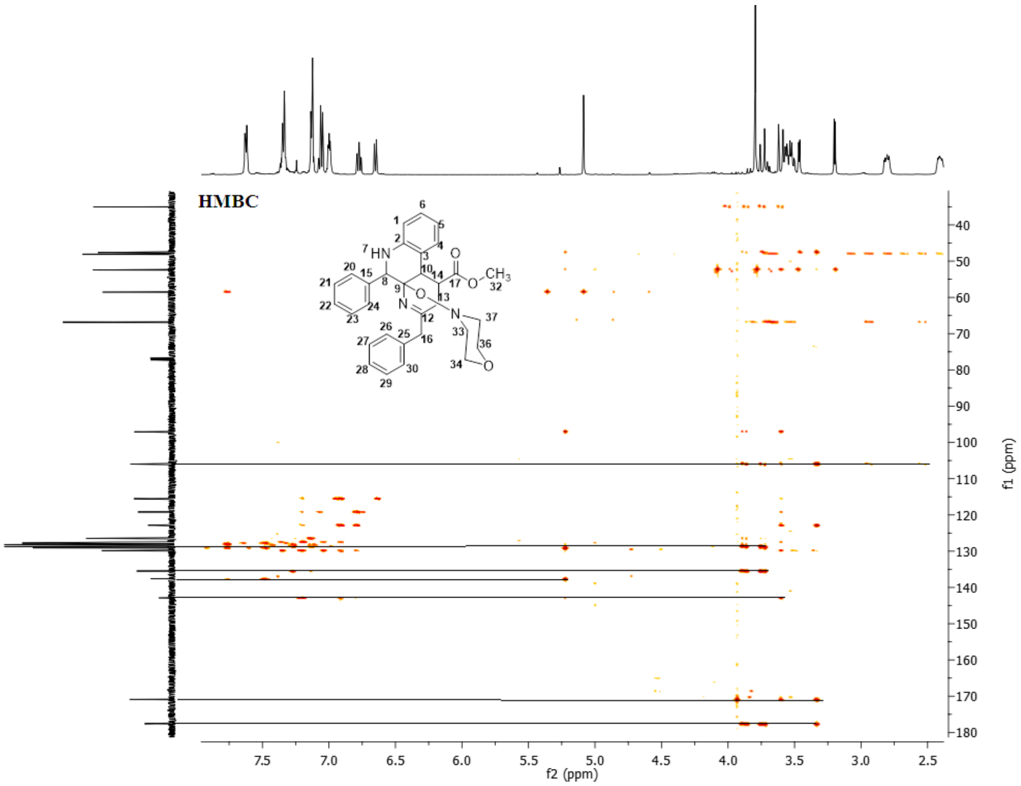

Figure S35. HMBC spectrum of the product **3a**

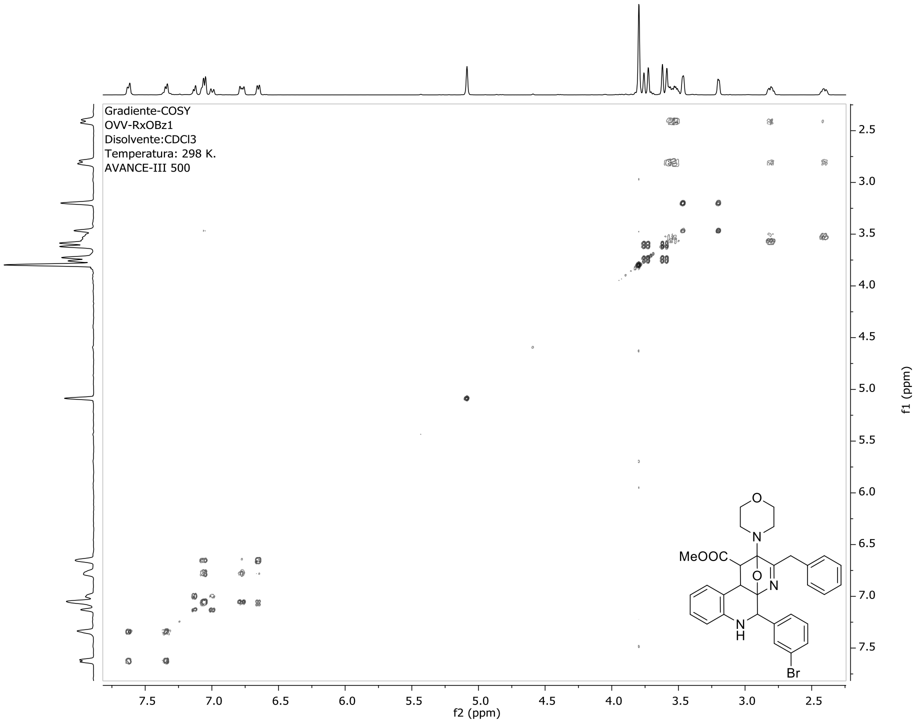

Figure S36. COSY spectrum of the product **3c**

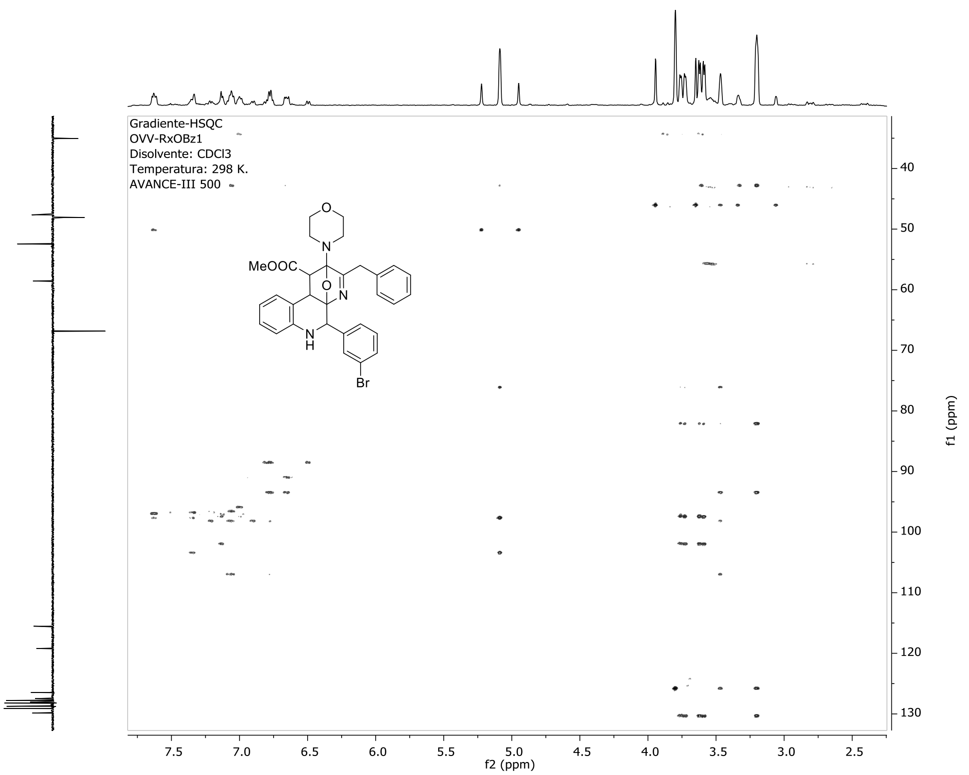

Figure S37. HSQC spectrum of the product **3c**

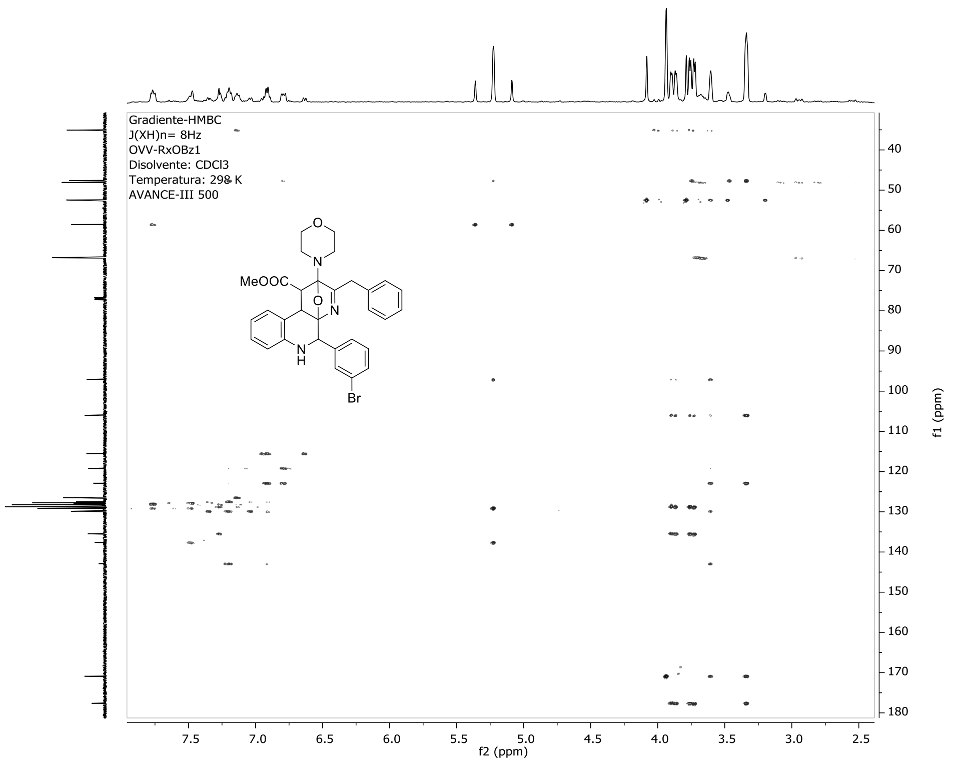

Figure S38. HMBC spectrum of the product **3c**

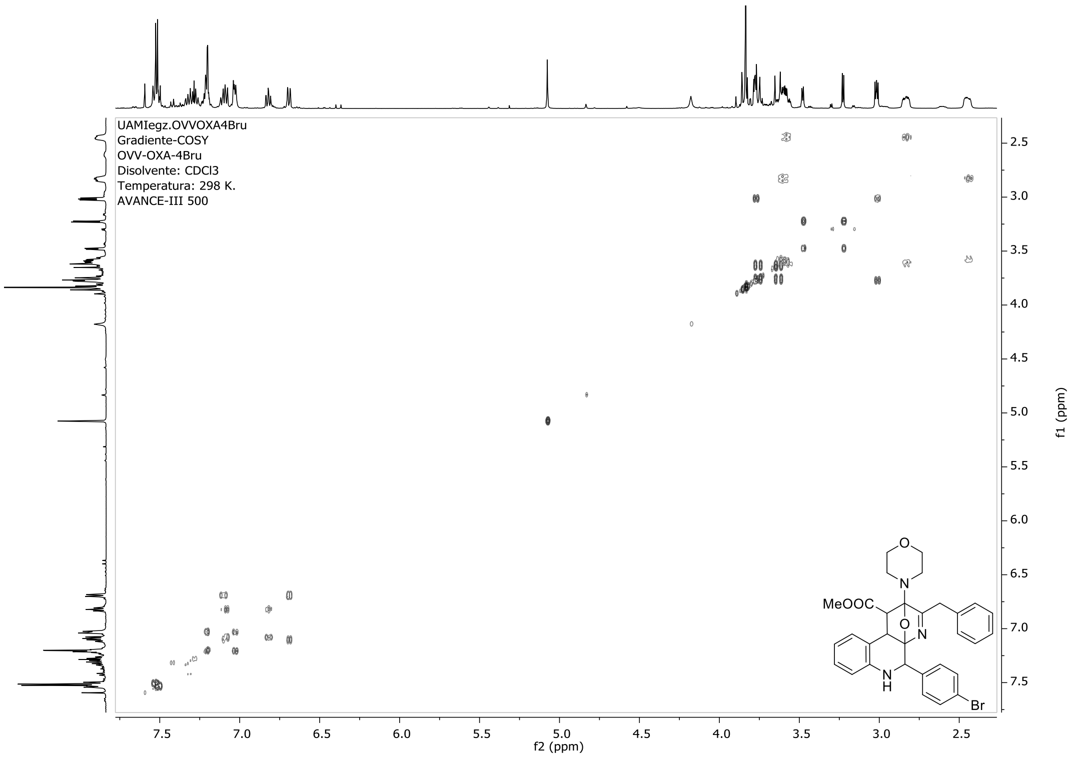

Figure S39. COSY spectrum of the product **3d**

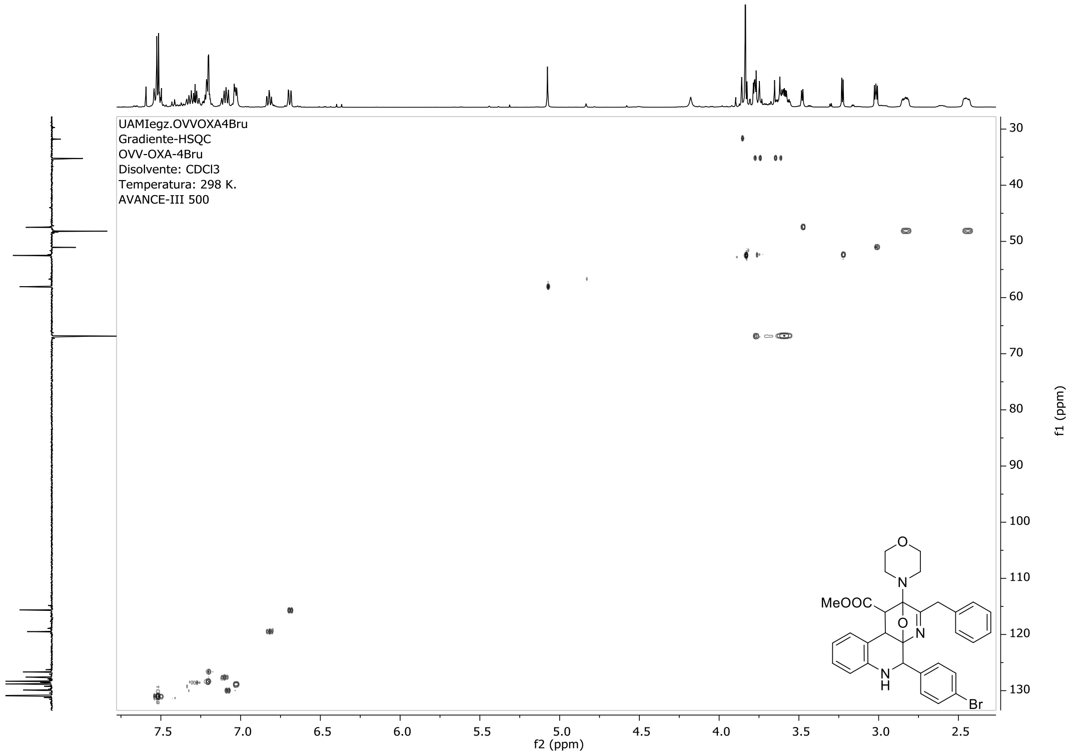

Figure S40. HSQC spectrum of the product **3d**

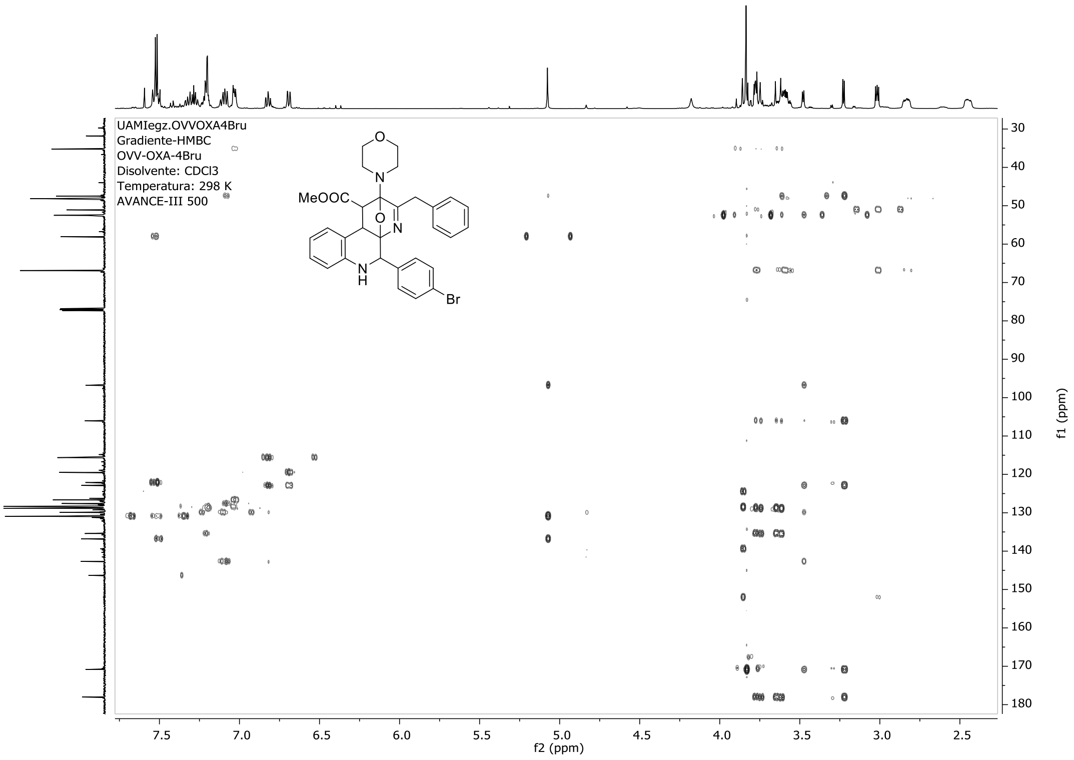

Figure S41. HMBC spectrum of the product **3d**

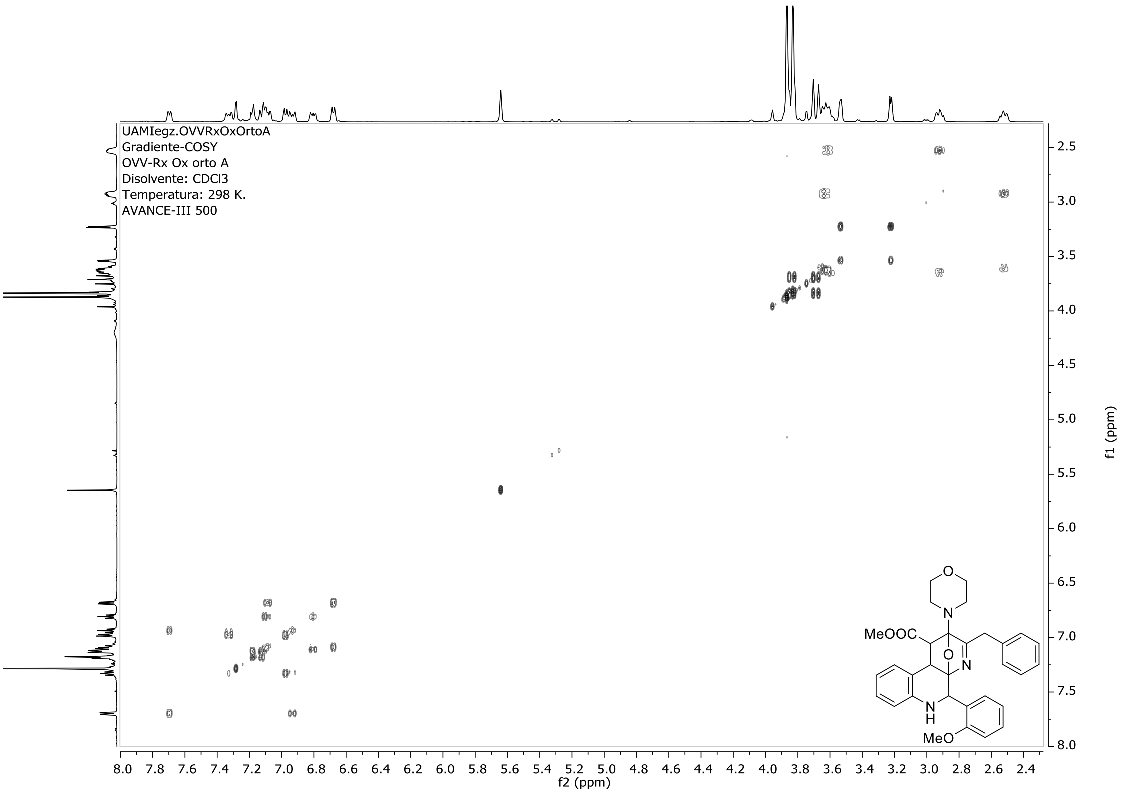

Figure S42. COSY spectrum of the product **3e**

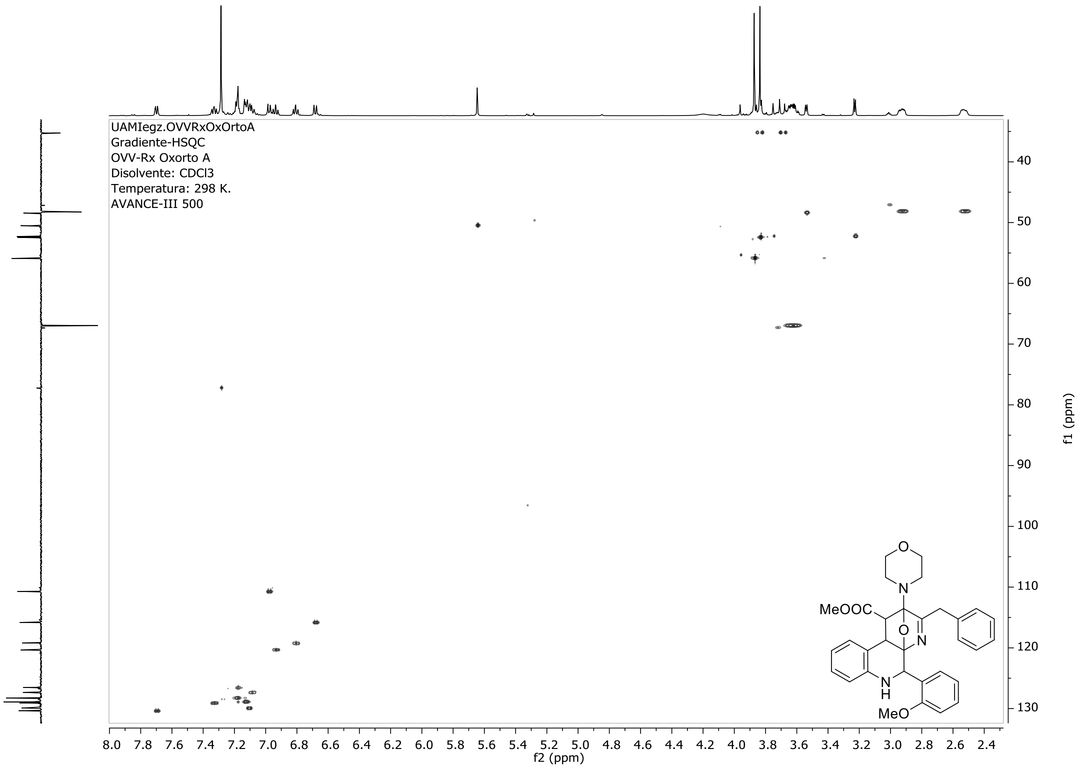

Figure S43. HSQC spectrum of the product **3e**

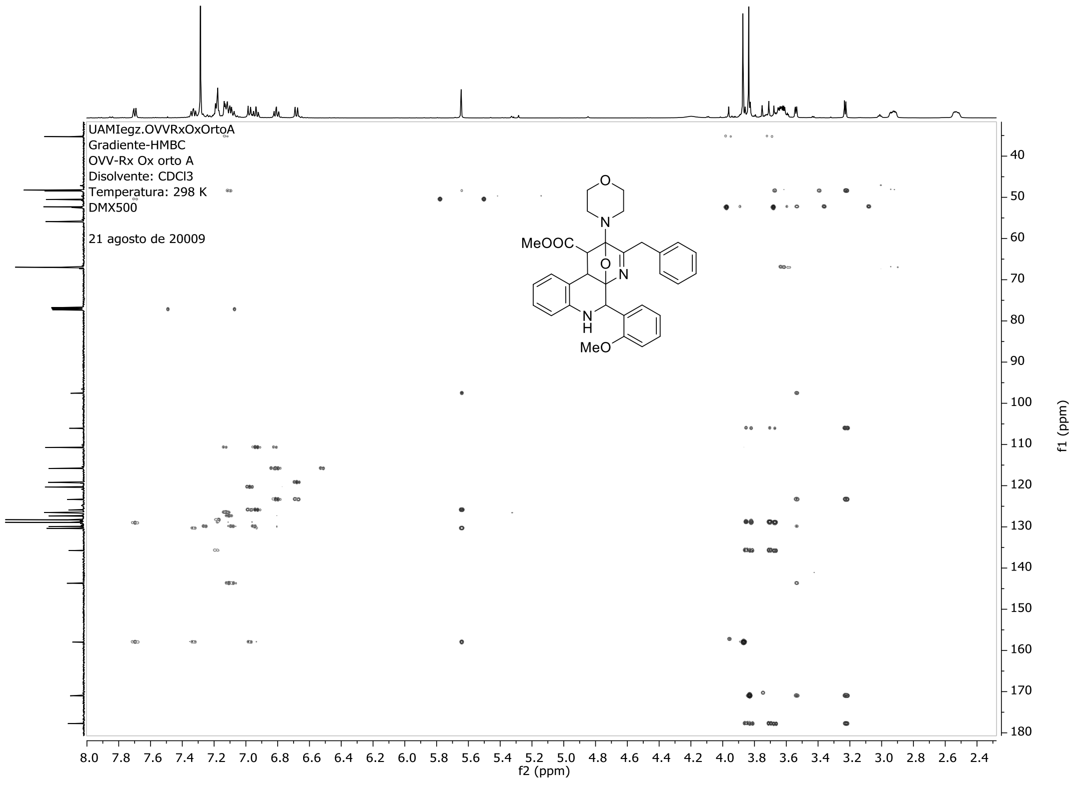

Figure S44. HMBC spectrum of the product **3e**

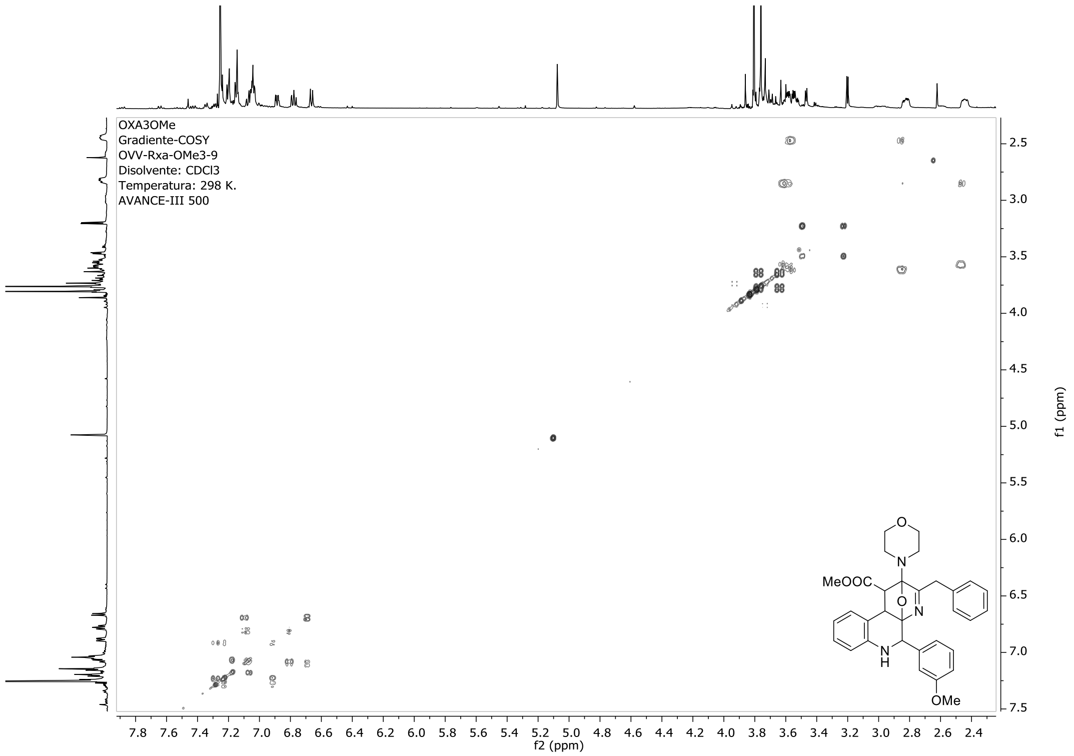

Figure S45. COSY spectrum of the product **3f**

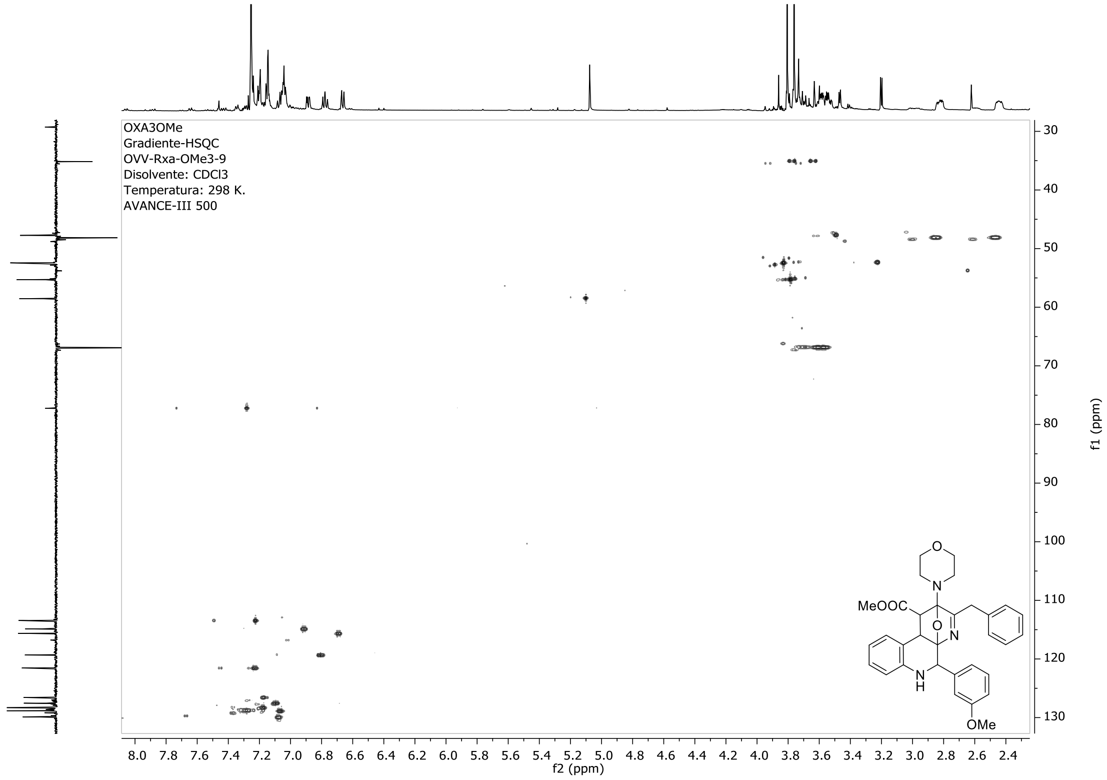

Figure S46. HSQC spectrum of the product **3f**

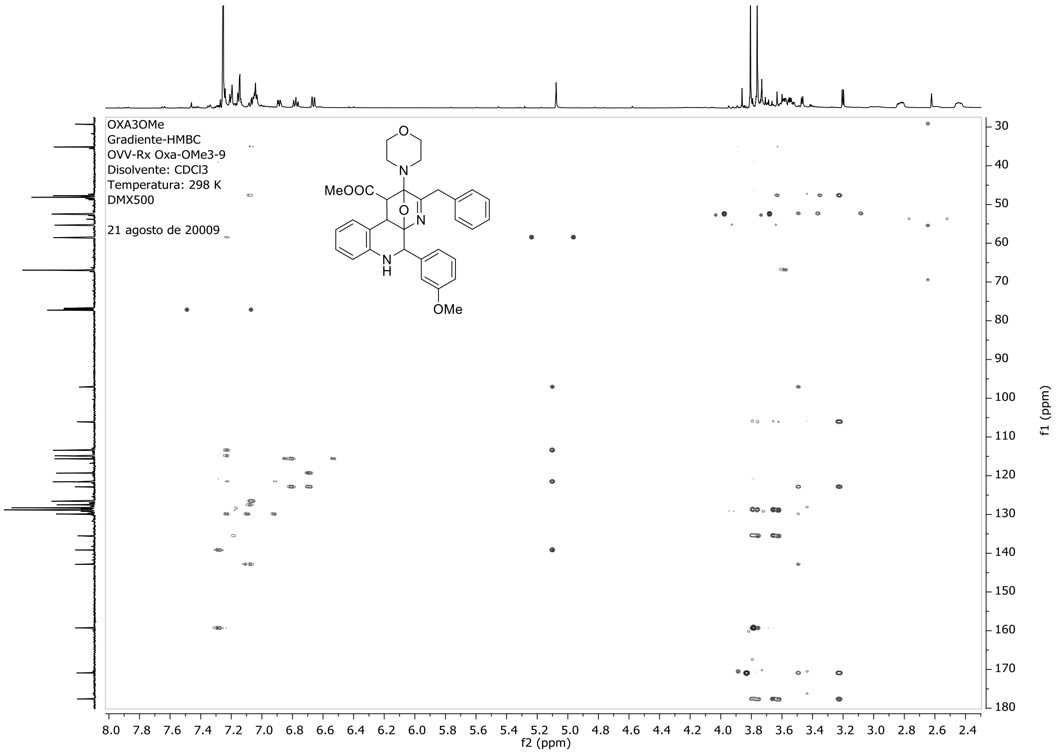

Figure S47. HMBC spectrum of the product **3f**

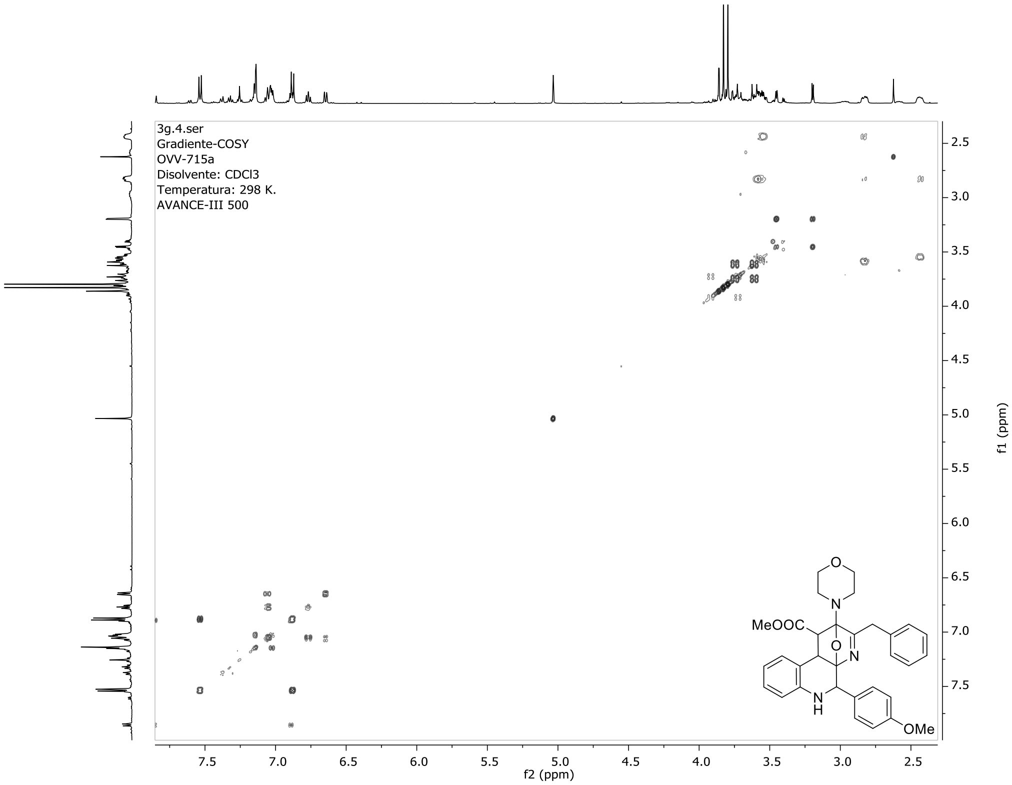

Figure S48. COSY spectrum of the product **3g**

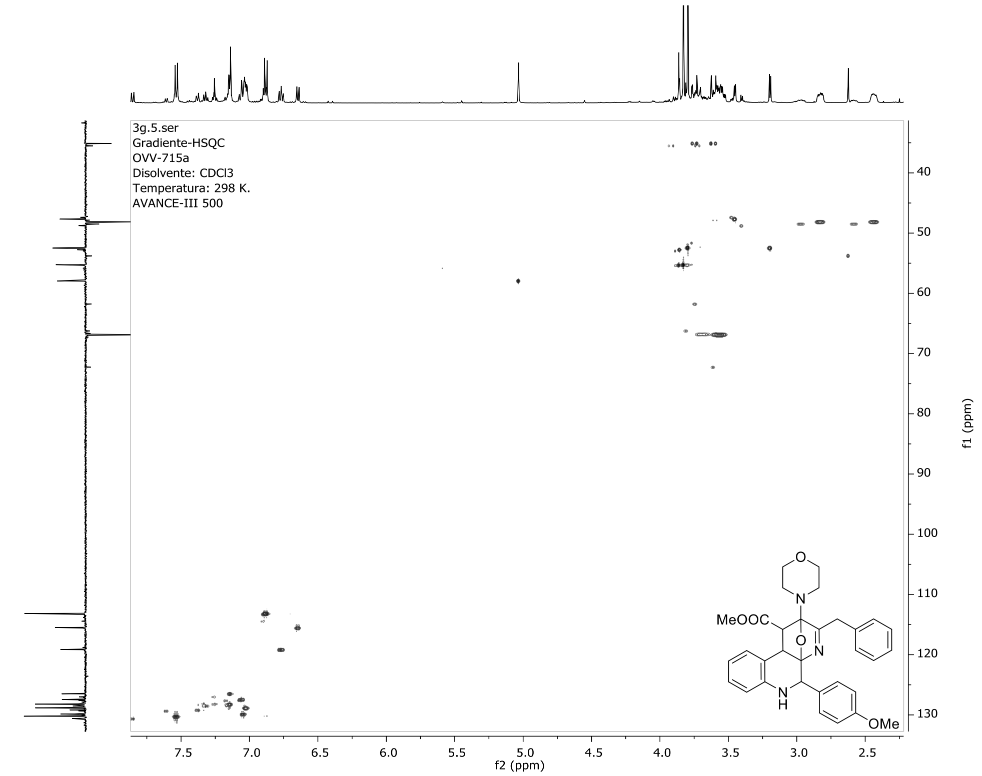

Figure S49. HSQC spectrum of the product **3g**

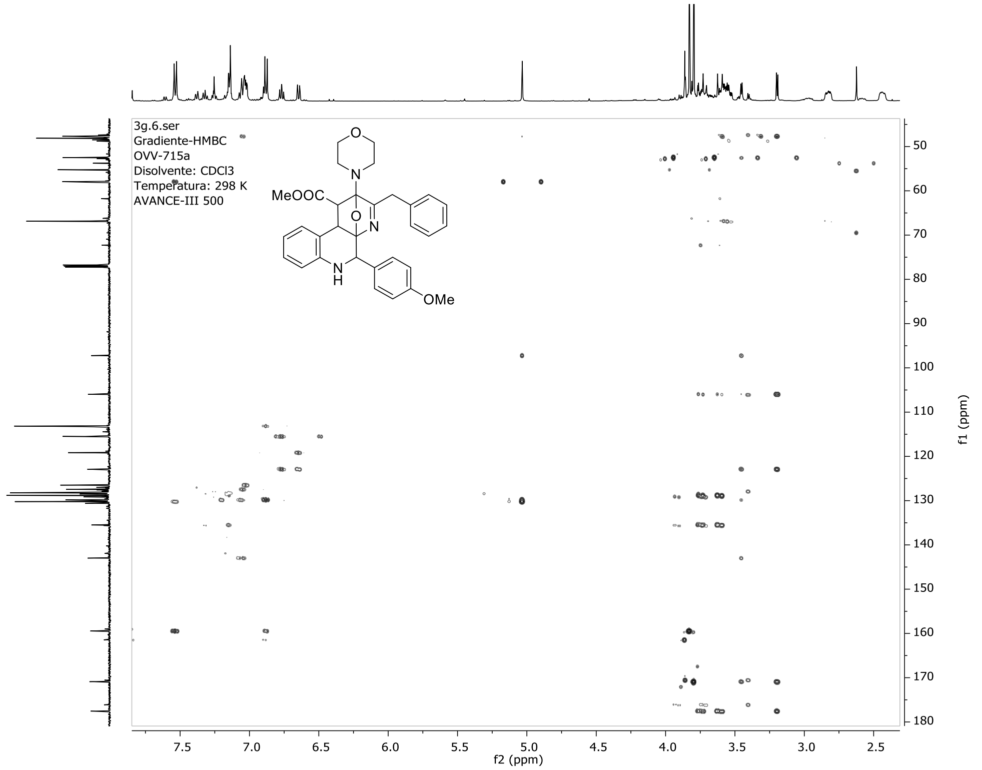

Figure S50. HMBC spectrum of the product **3g**

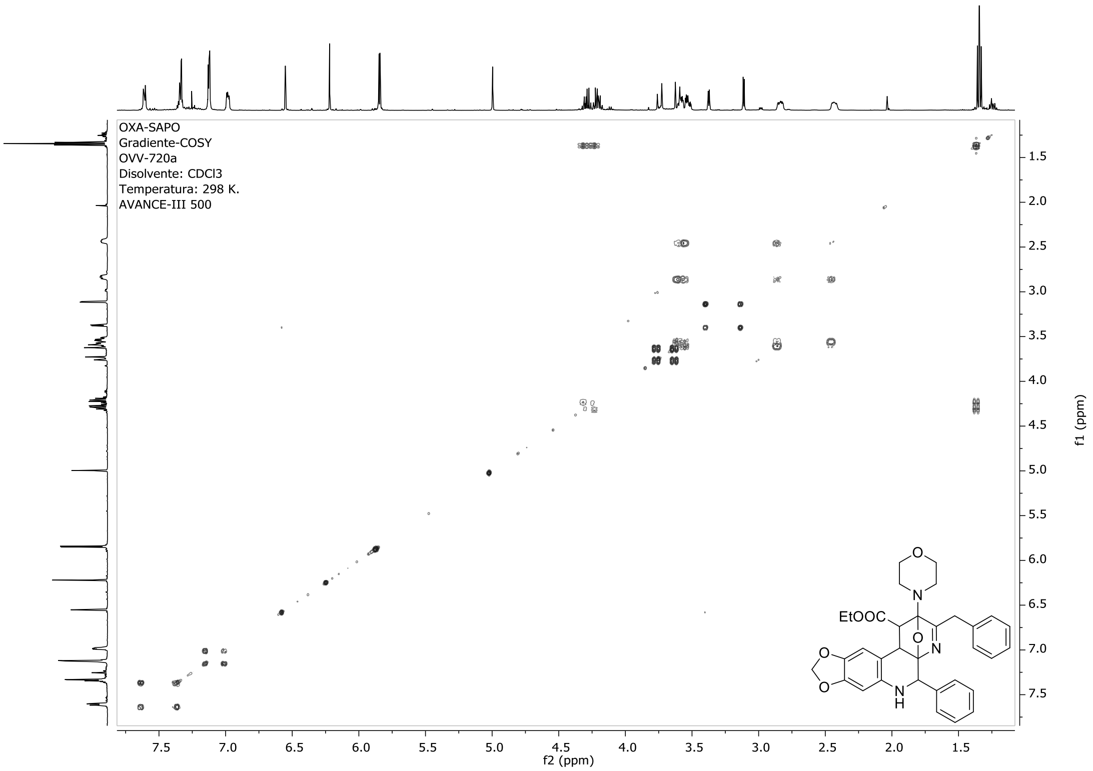

Figure S51. COSY spectrum of the product **3h**

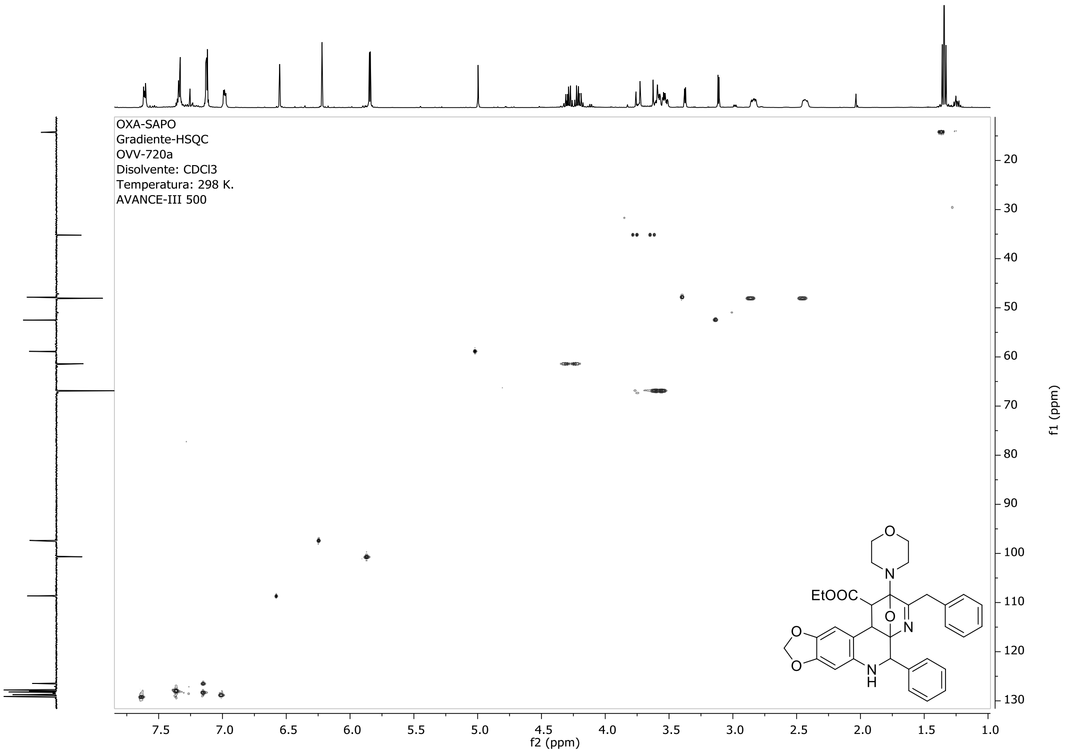

Figure S52. HSQC spectrum of the product **3h**

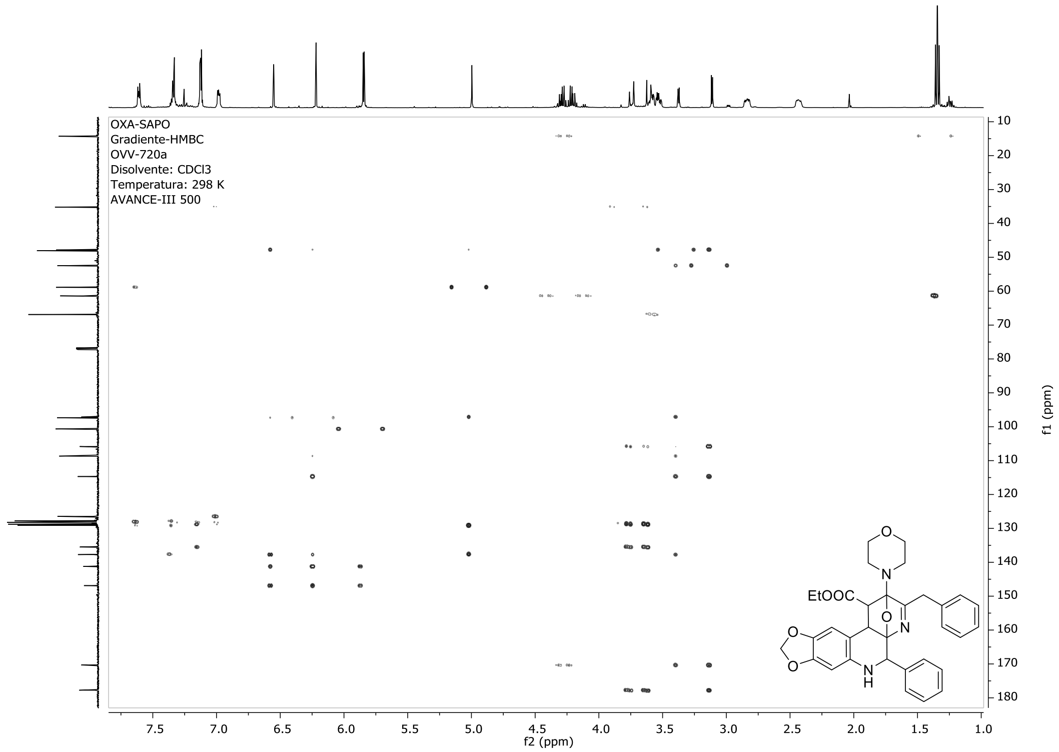

Figure S53. HMBC spectrum of the product **3h**

2D-NMR spectra for the products **4a–h**

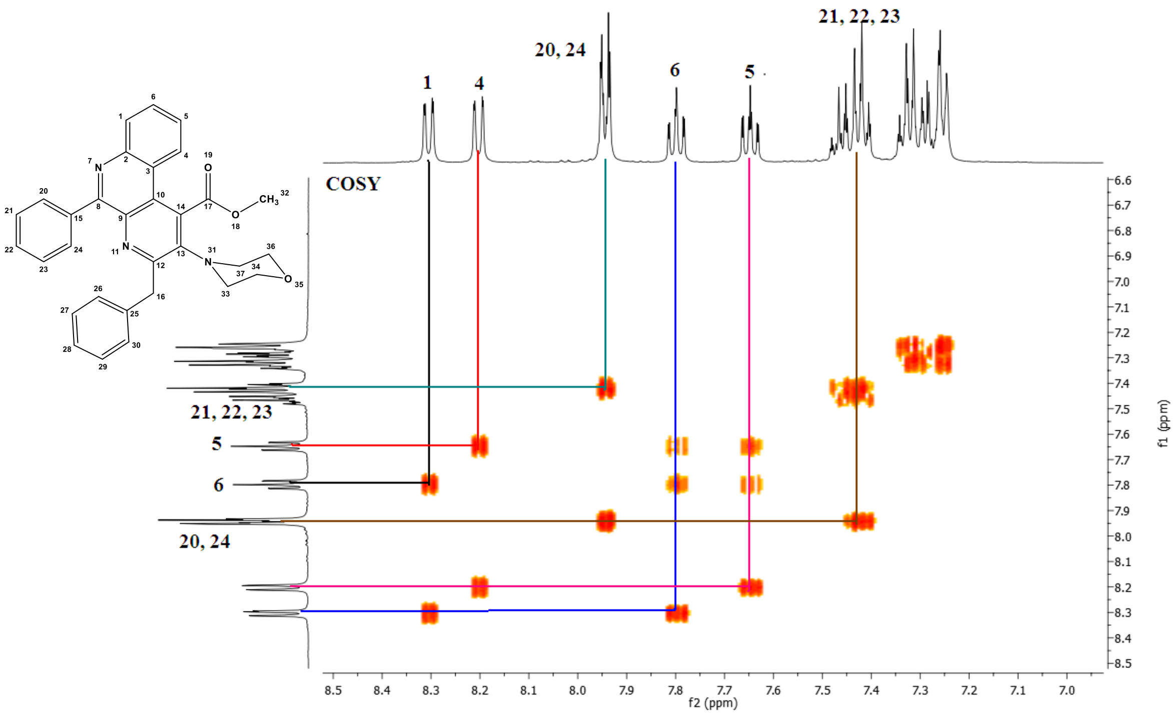

Figure S54. COSY spectrum of the product **4a**

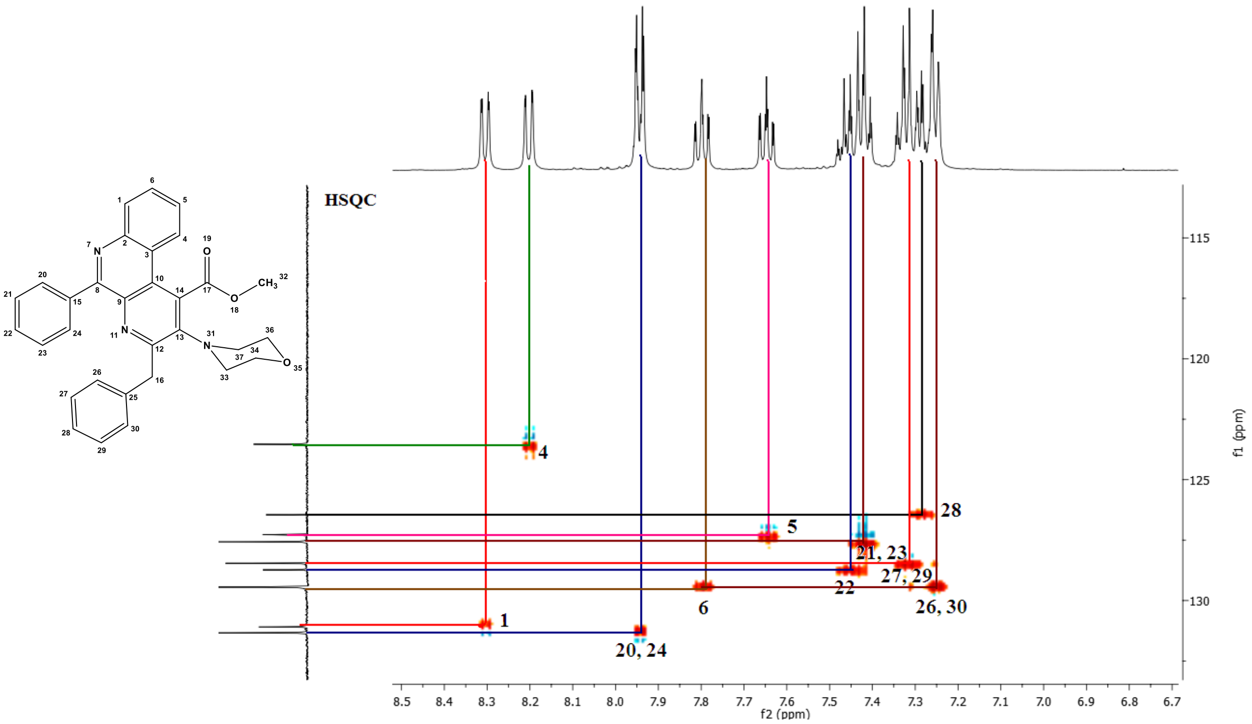

Figure S55. HSQC spectrum of the product **4a**

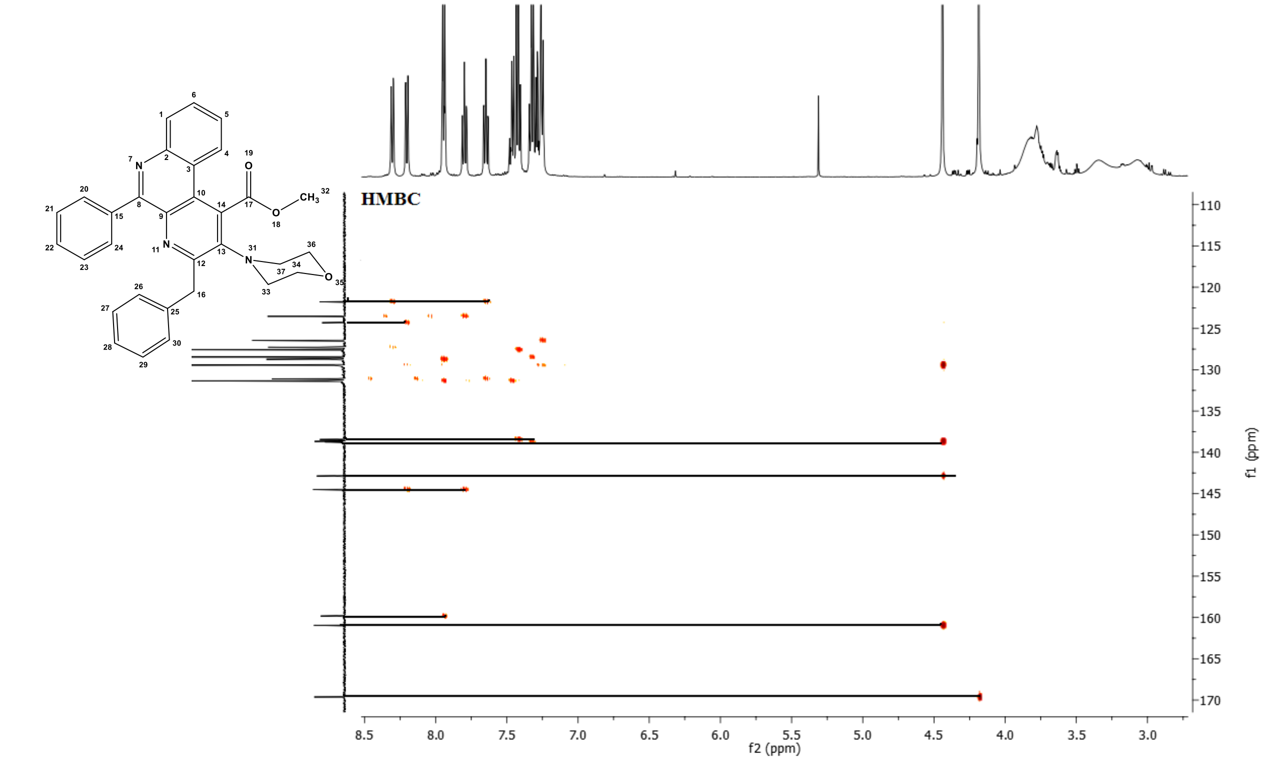

Figure S56. HMBC spectrum of the product **4a**

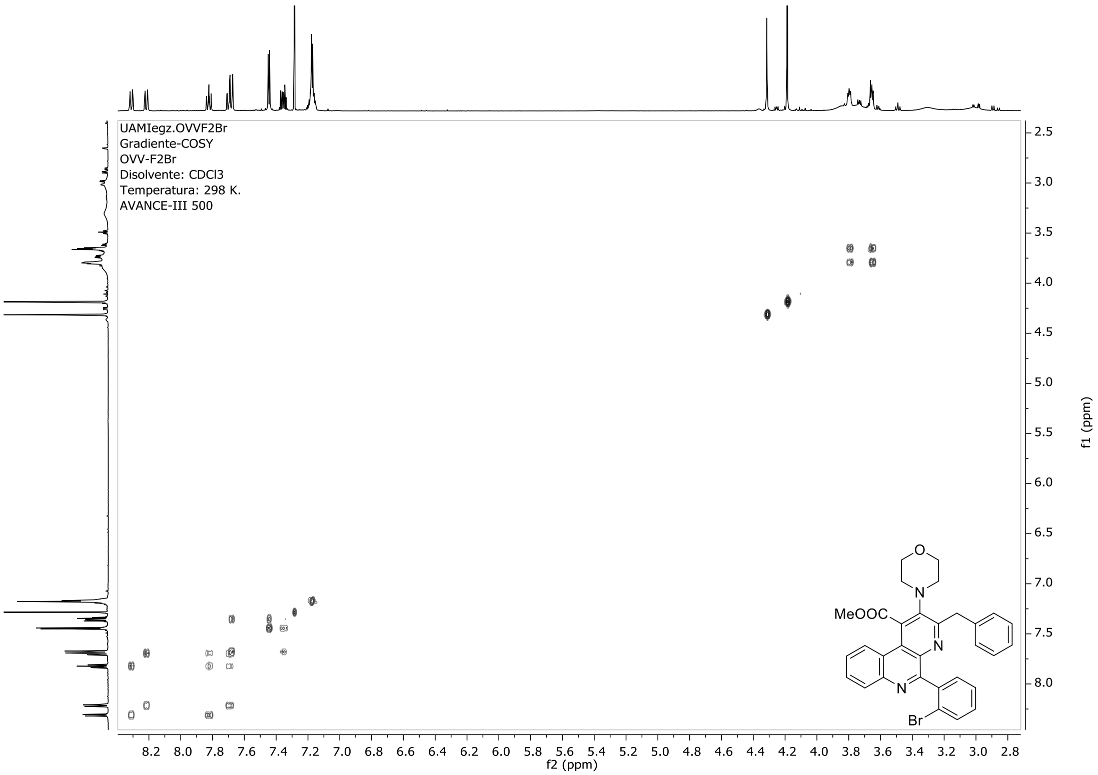

Figure S57. COSY spectrum of the product **4b**

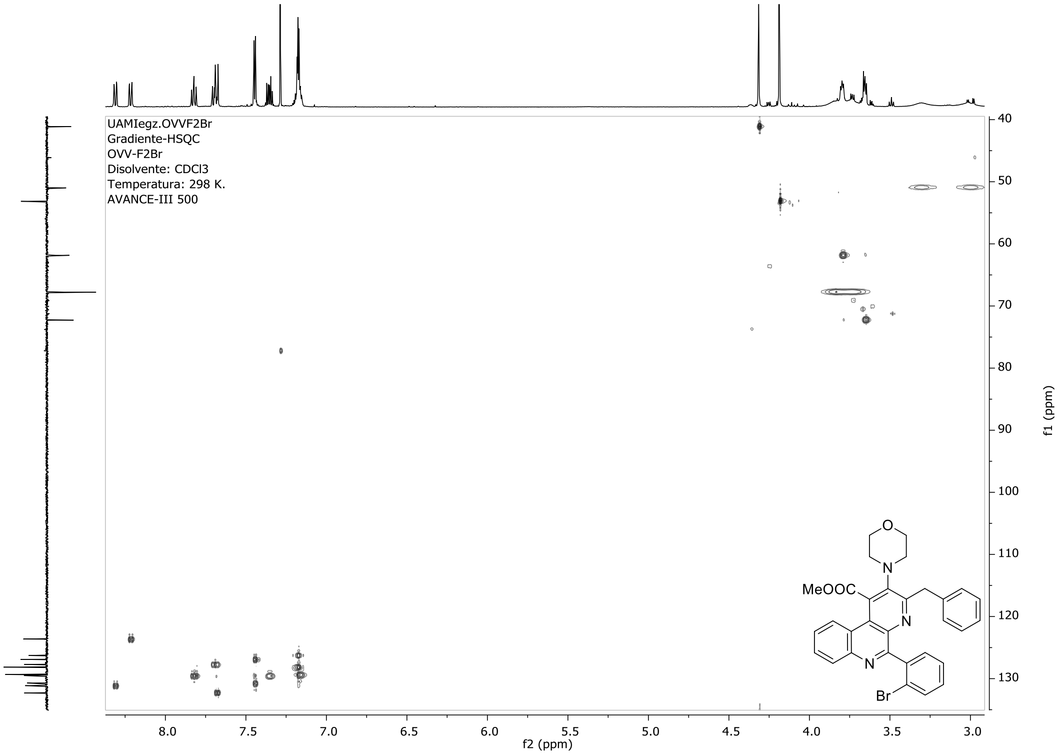

Figure S58. HSQC spectrum of the product **4b**

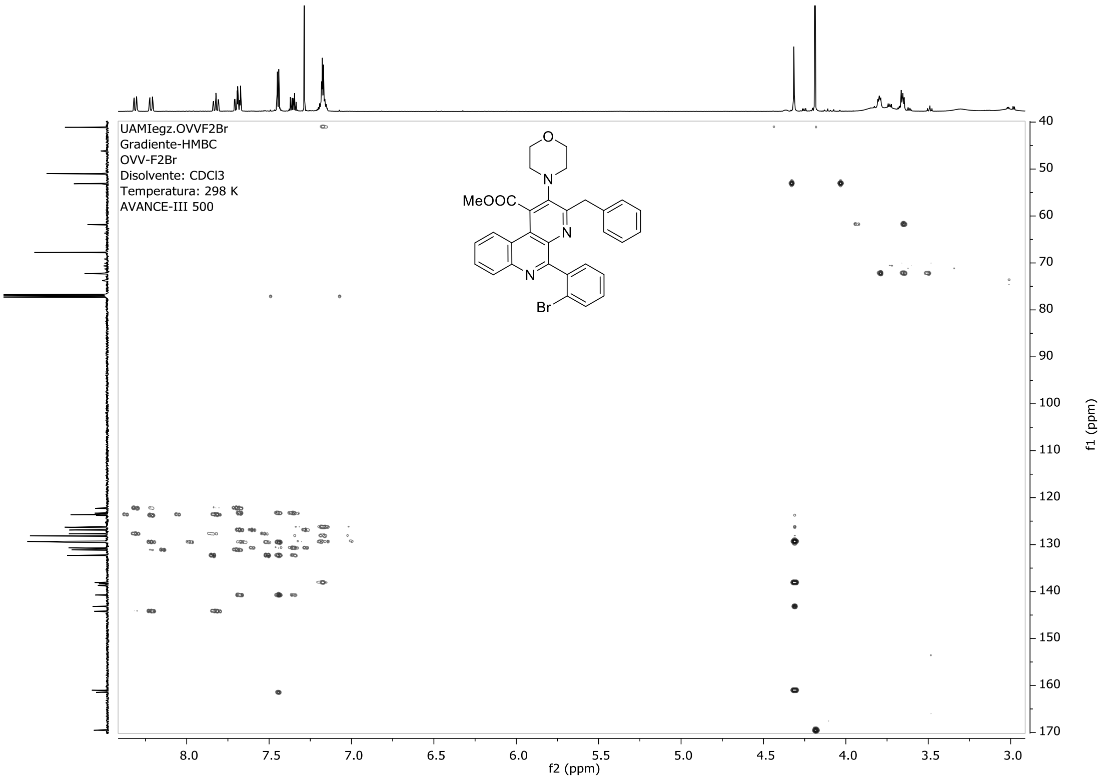

Figure S59. HMBC spectrum of the product **4b**

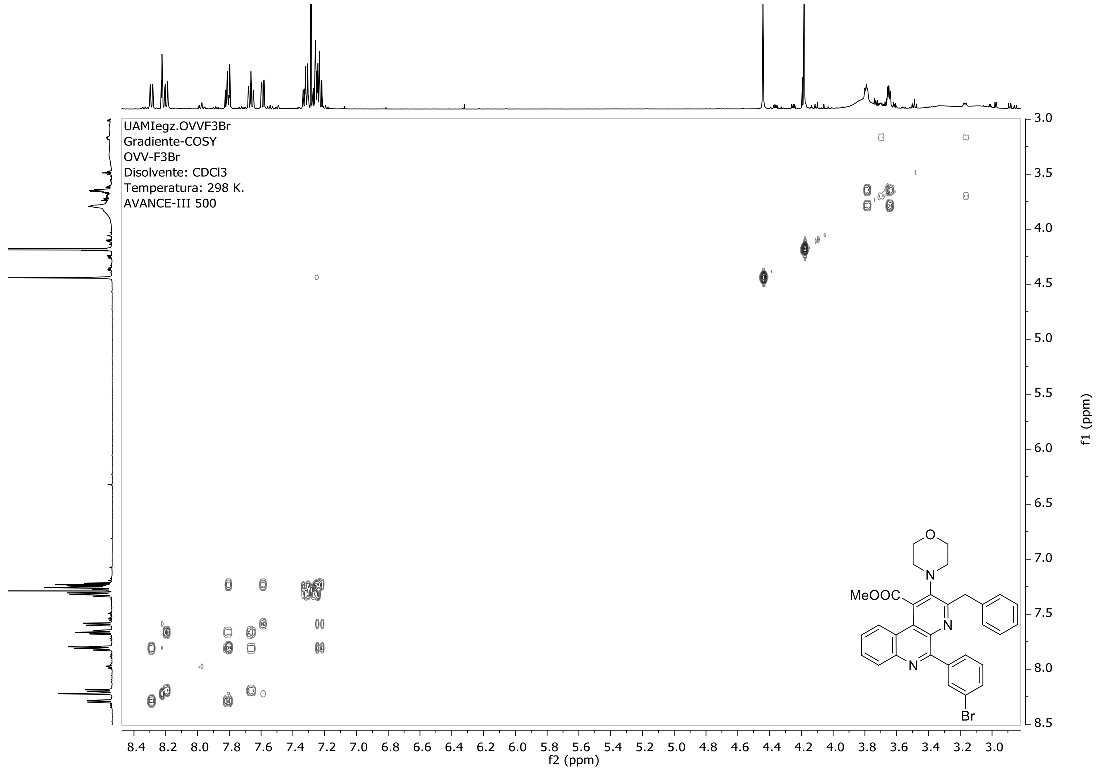

Figure S60. COSY spectrum of the product **4c**

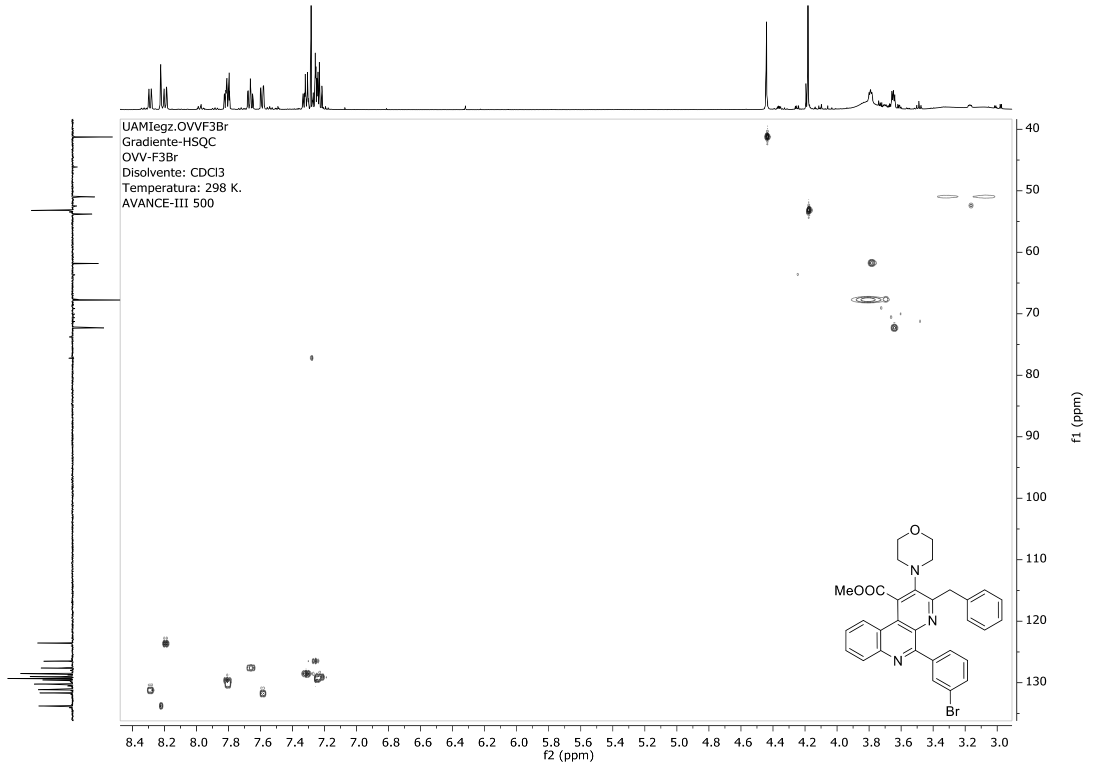

Figure S61. HSQC spectrum of the product **4c**

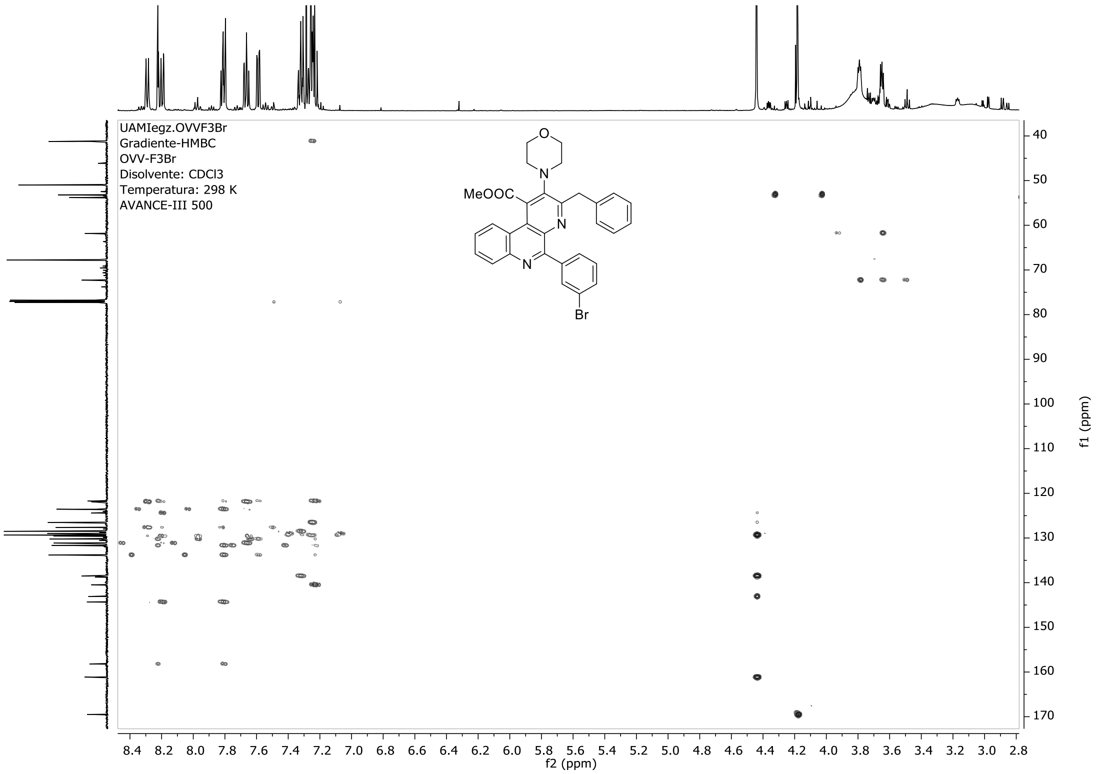

Figure S62. HMBC spectrum of the product **4c**

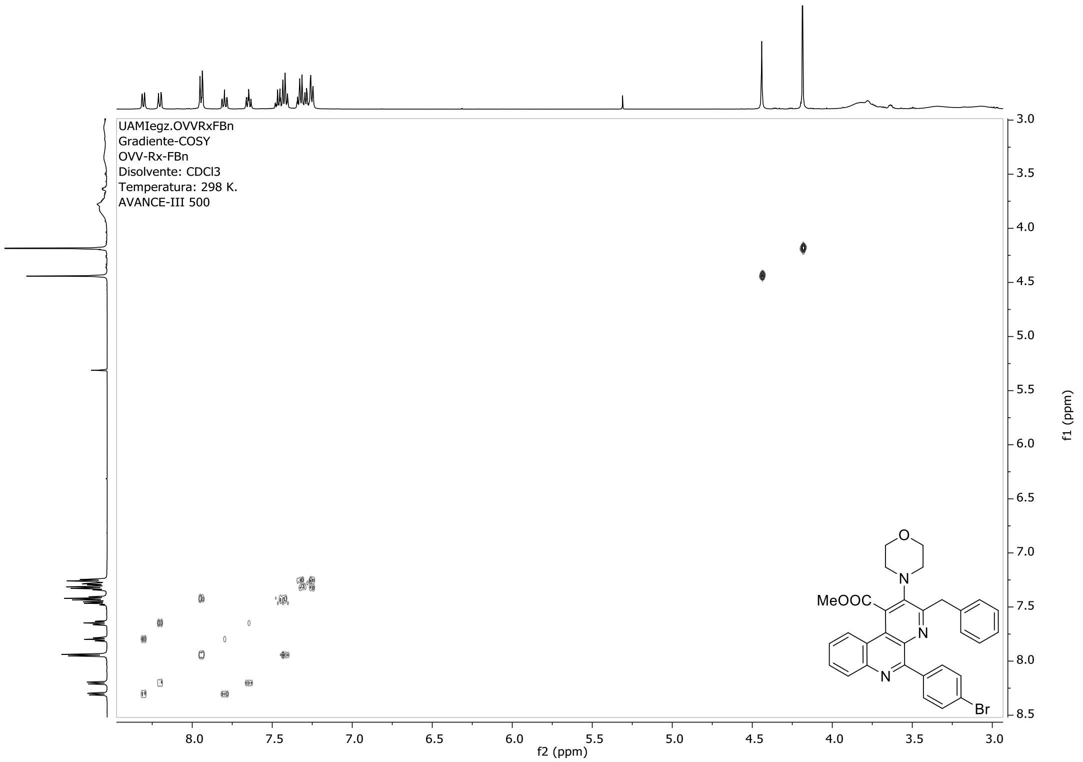

Figure S63. COSY spectrum of the product **4d**

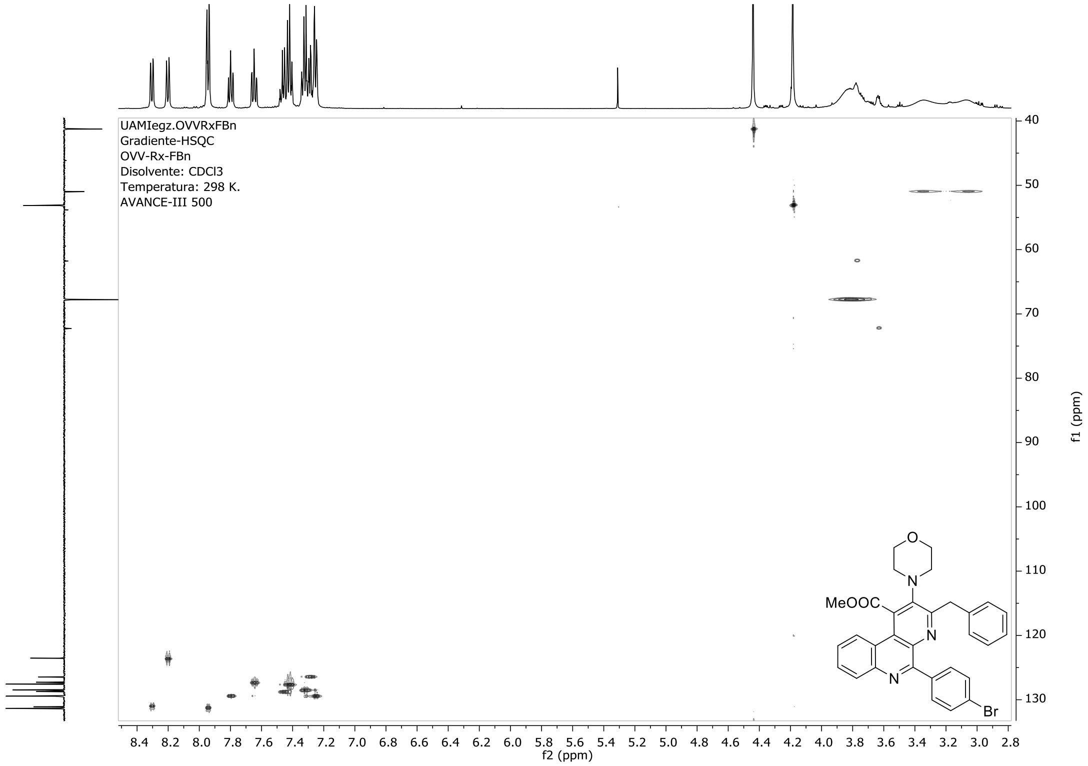

Figure S64. HSQC spectrum of the product **4d**

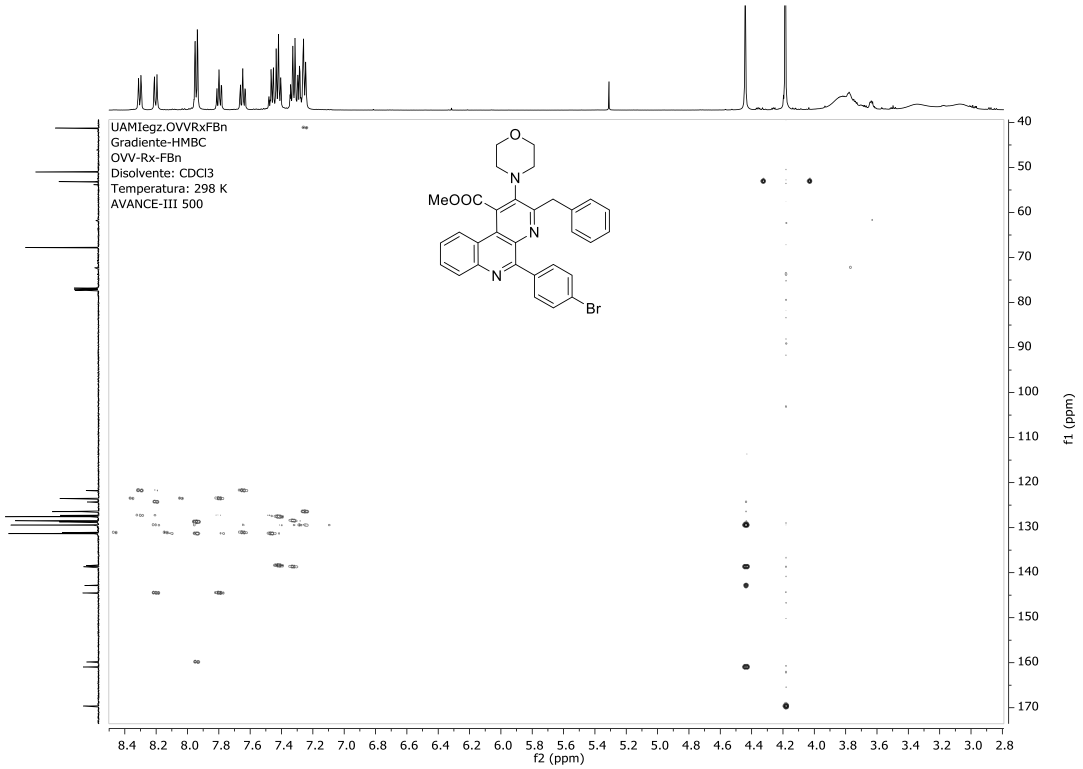

Figure S65. HMBC spectrum of the product **4d**

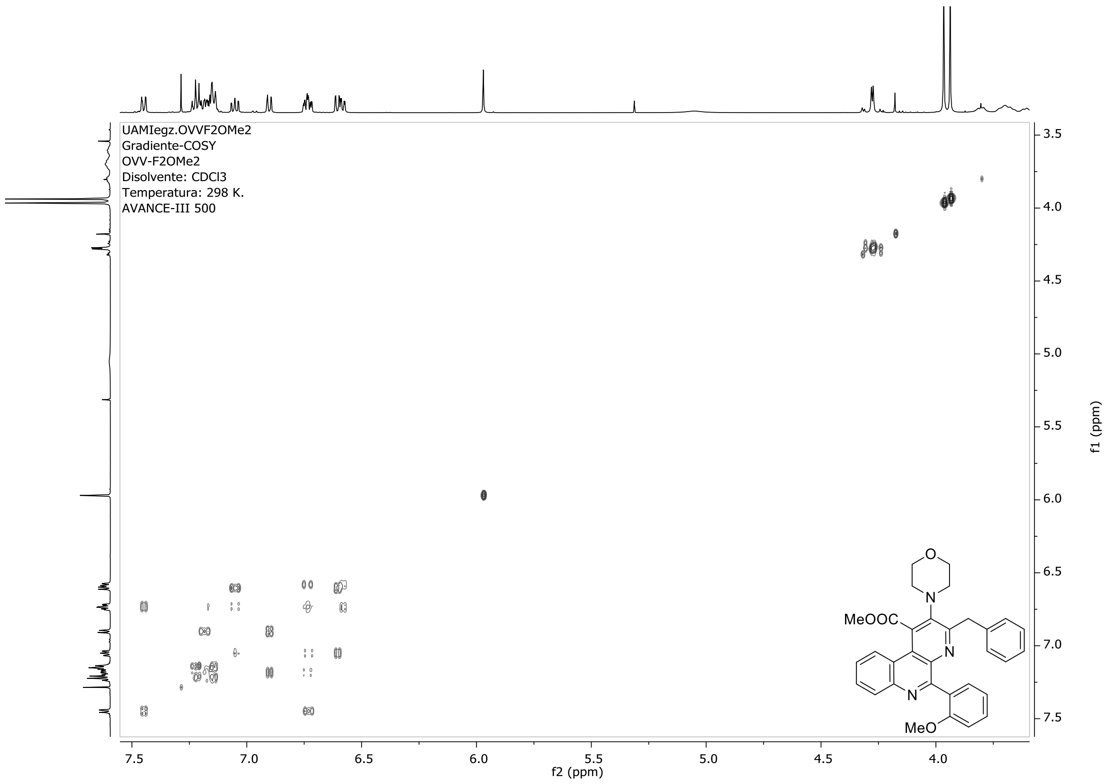

Figure S66. COSY spectrum of the product **4e**

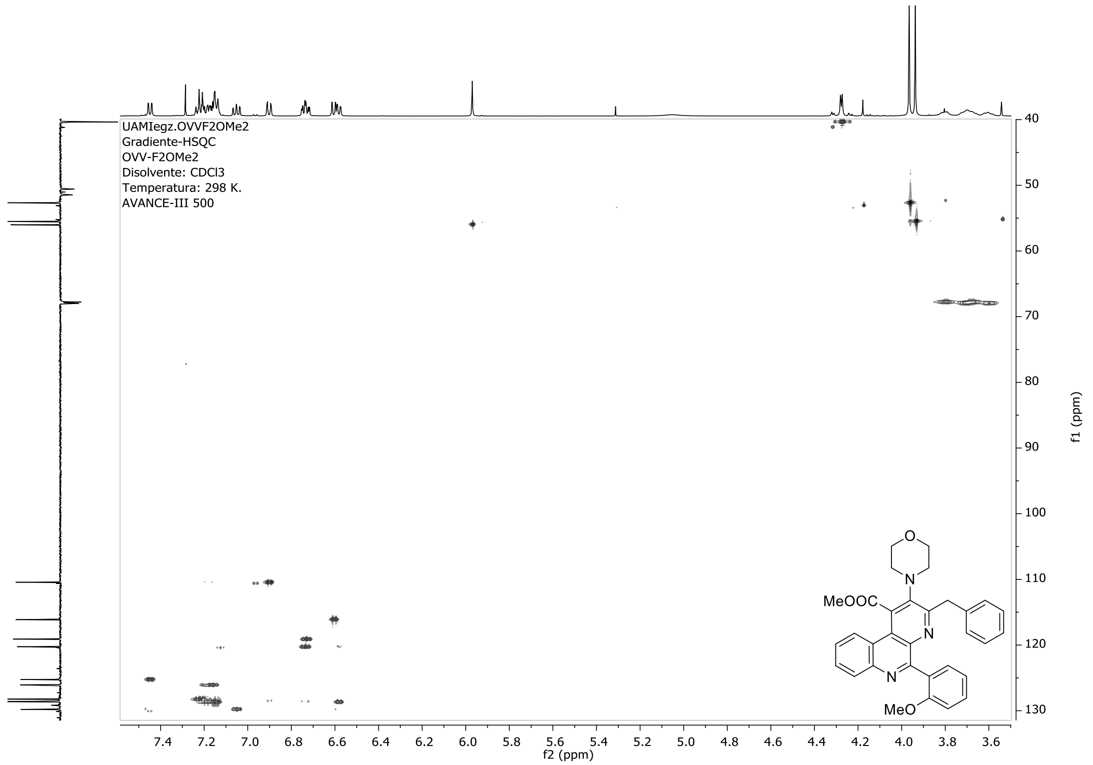

Figure S67. HSQC spectrum of the product **4e**

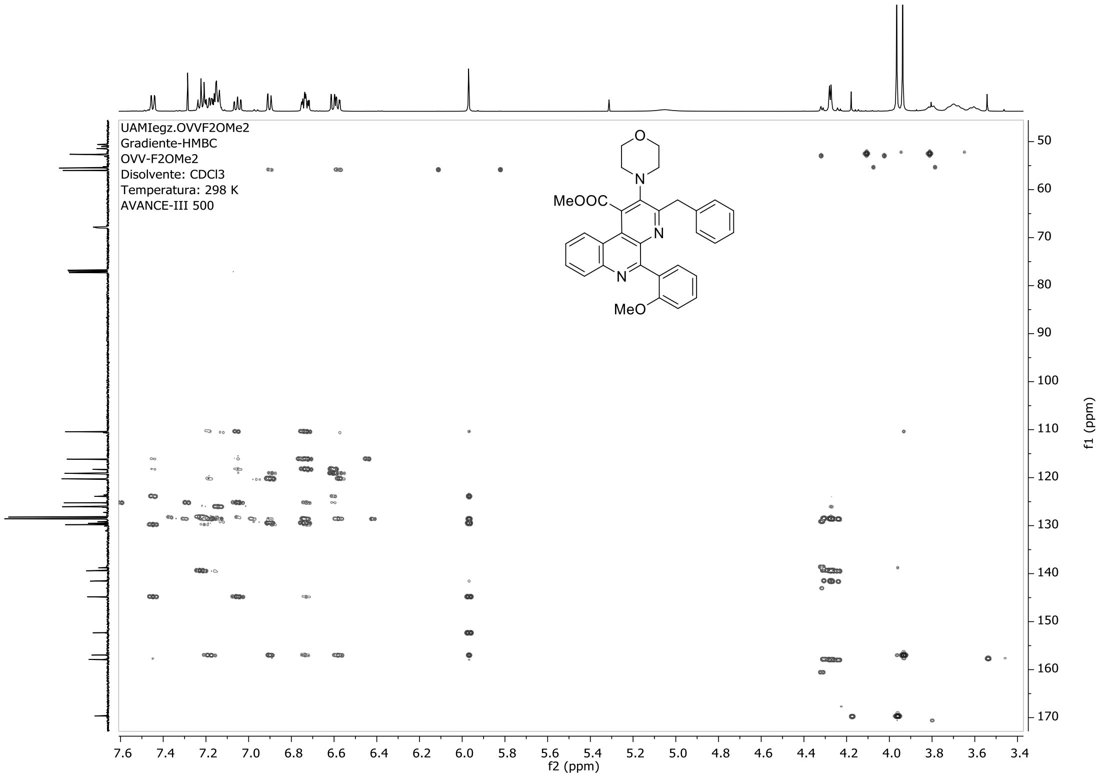

Figure S68. HMBC spectrum of the product **4e**

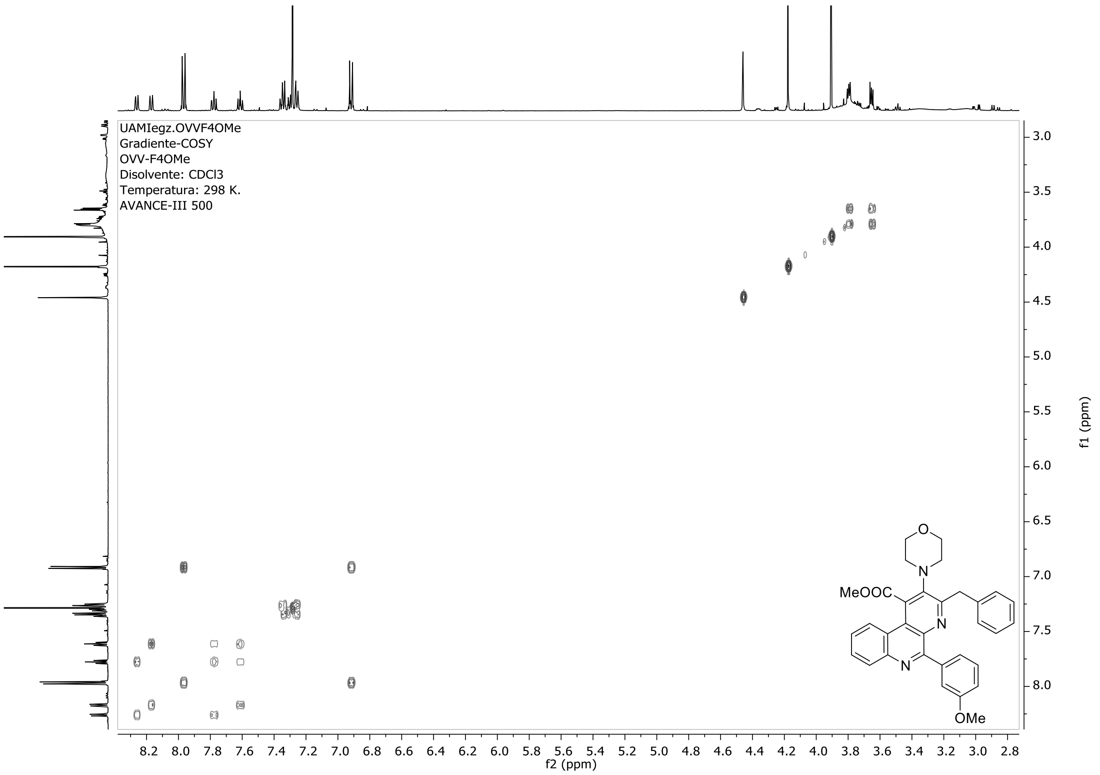

Figure S69. COSY spectrum of the product **4f**

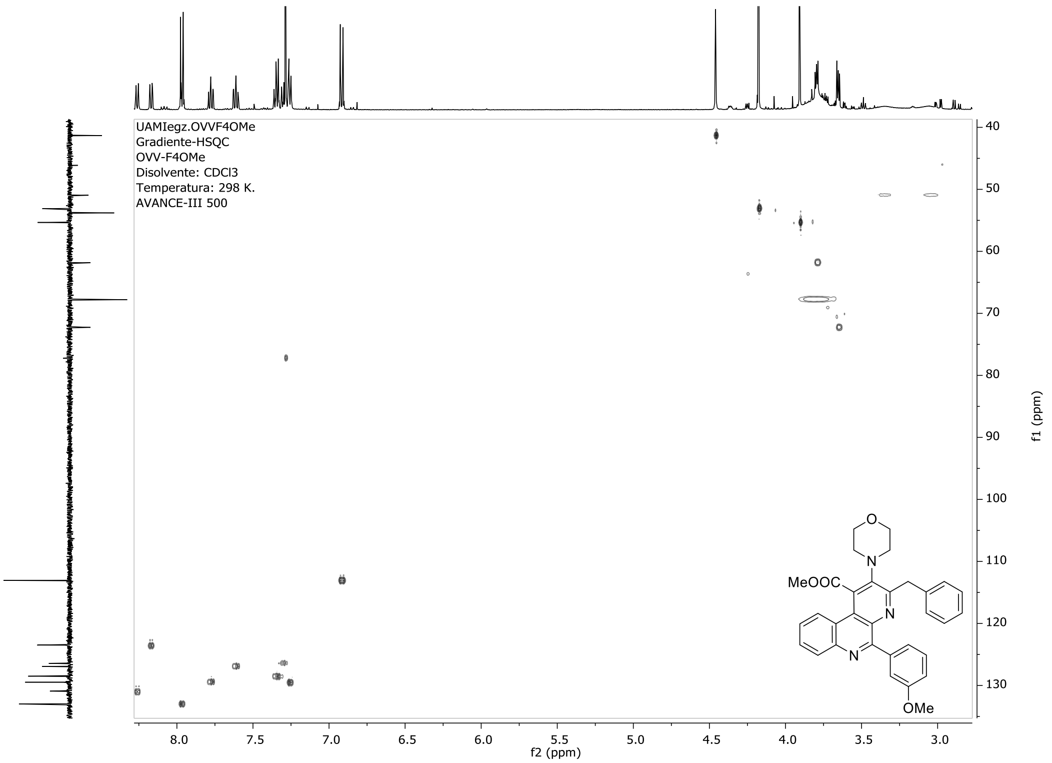

Figure S70. HSQC spectrum of the product **4f**

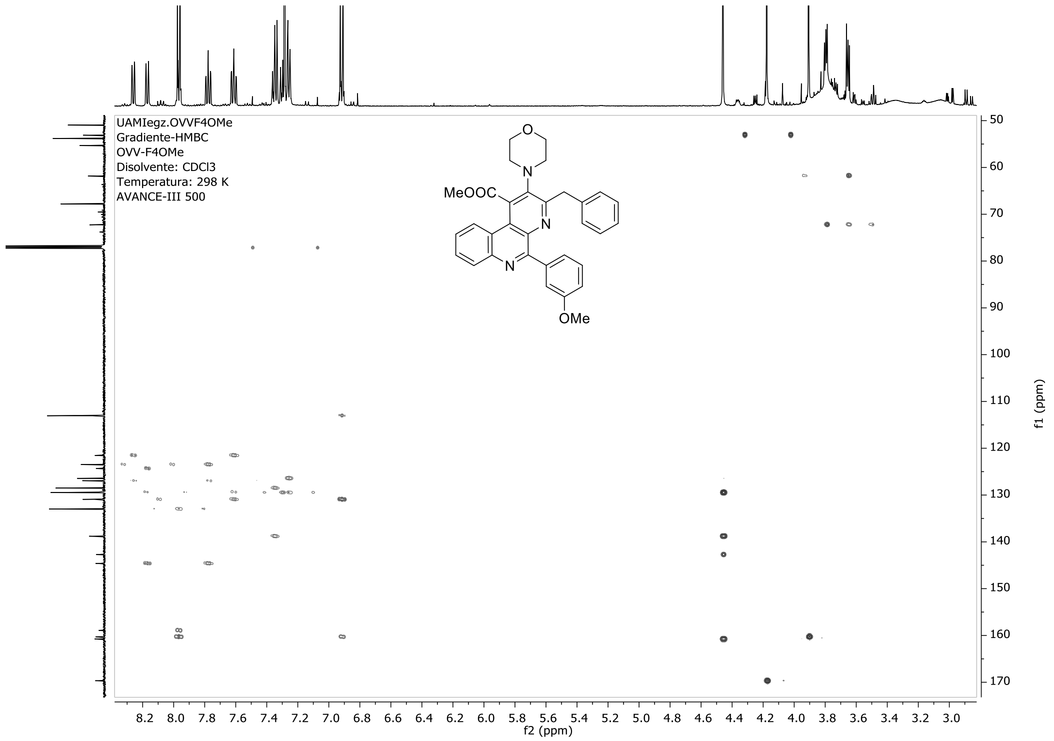

Figure S71. HMBC spectrum of the product **4f**

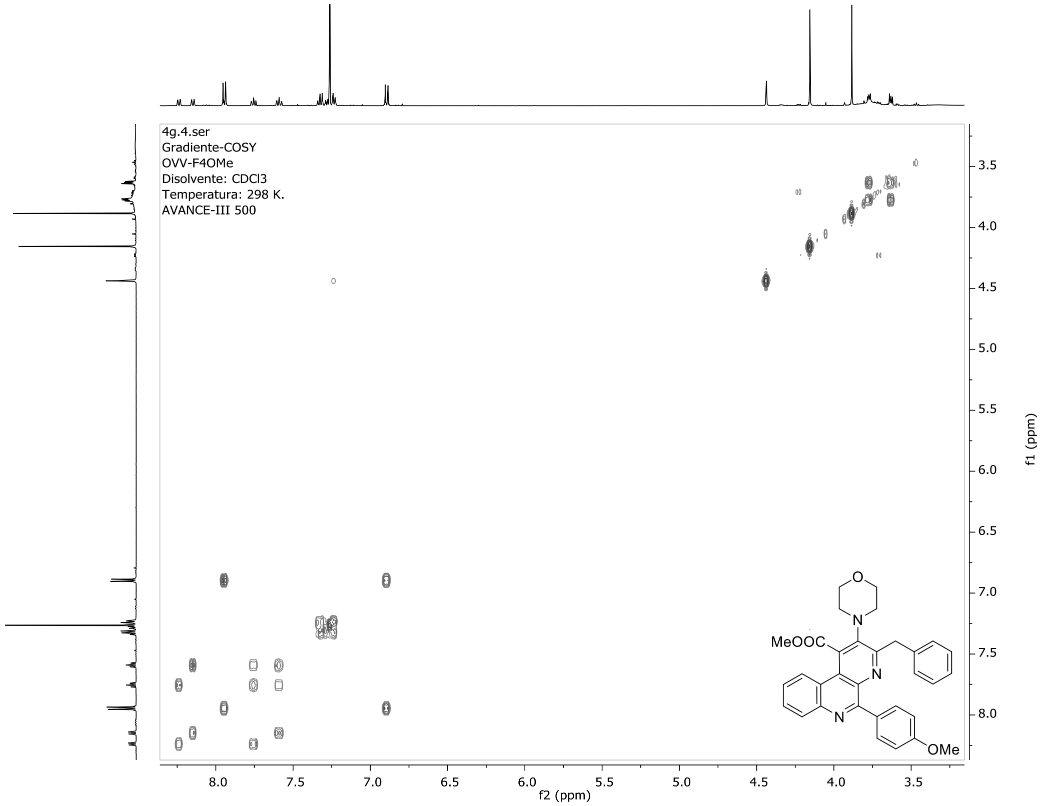

Figure S72. COSY spectrum of the product **4g**

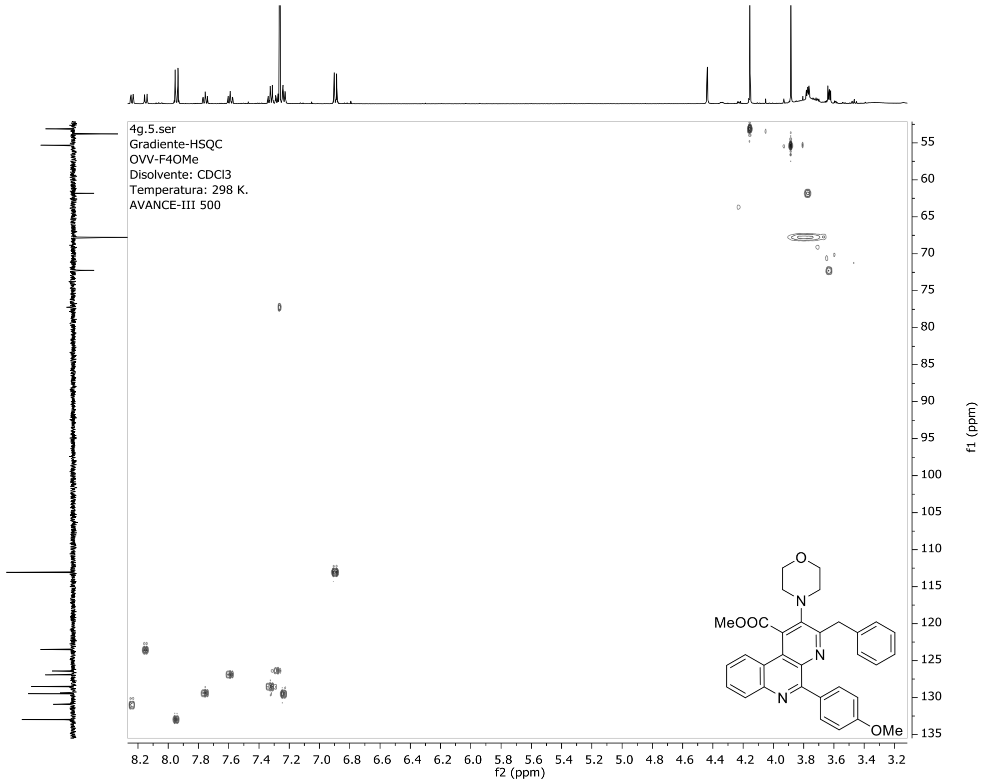

Figure S73. HSQC spectrum of the product **4g**

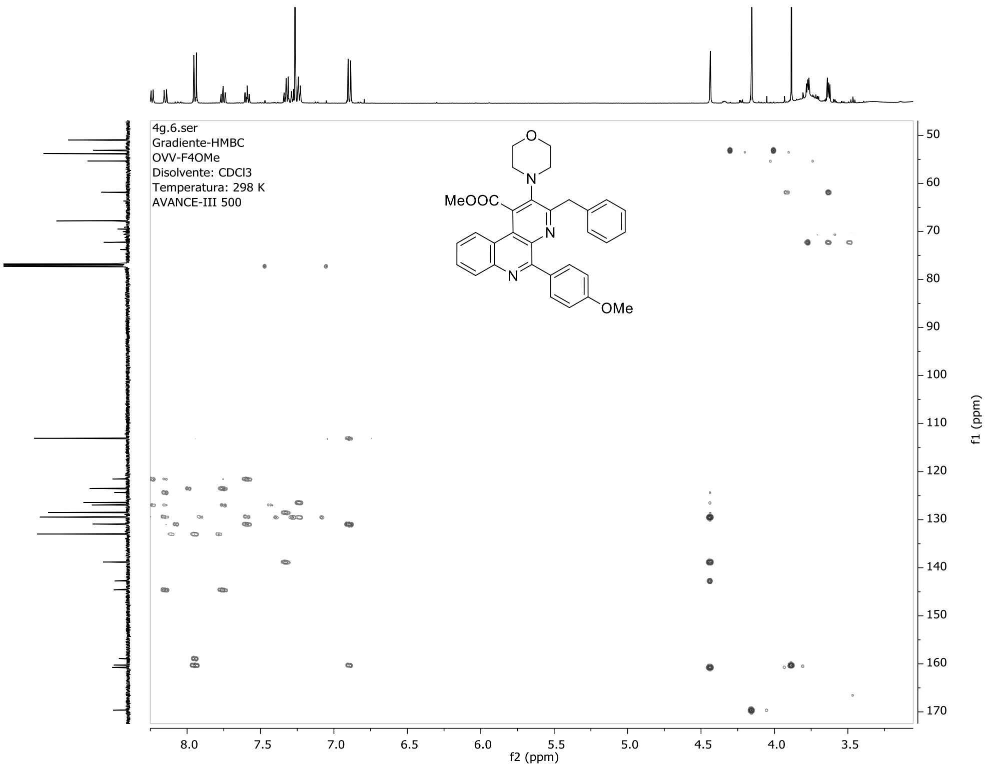

Figure S74. HMBC spectrum of the product **4g**

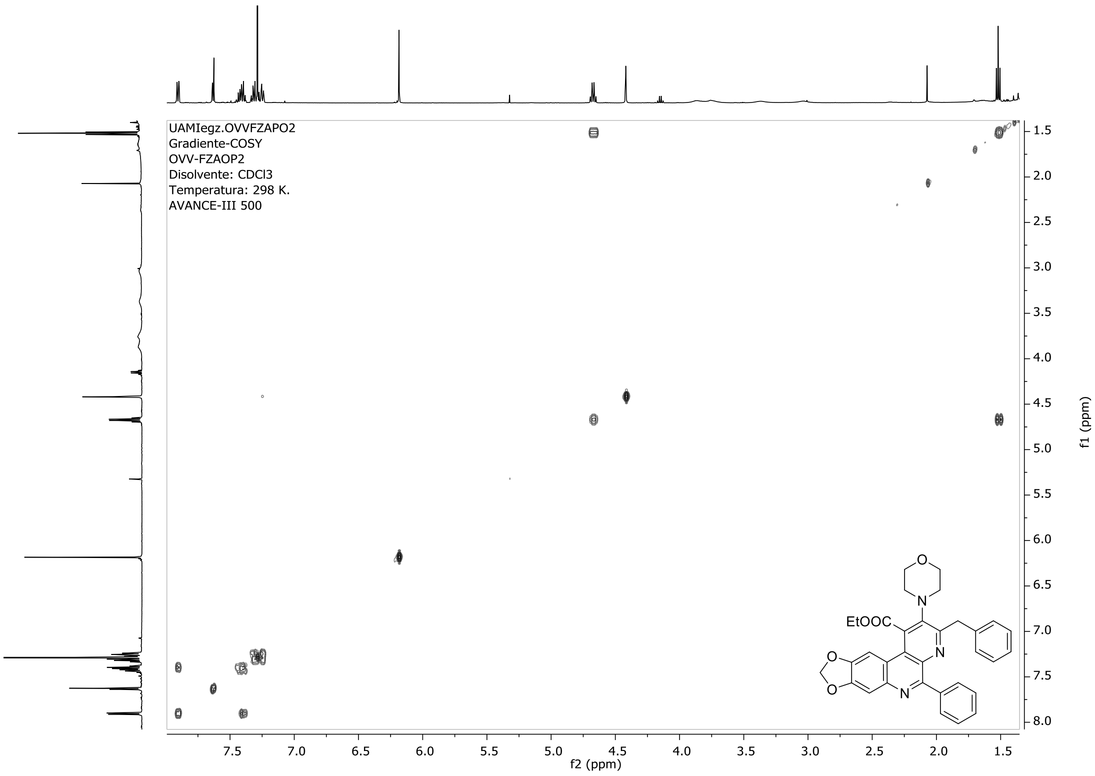

Figure S75. COSY spectrum of the product **4h**

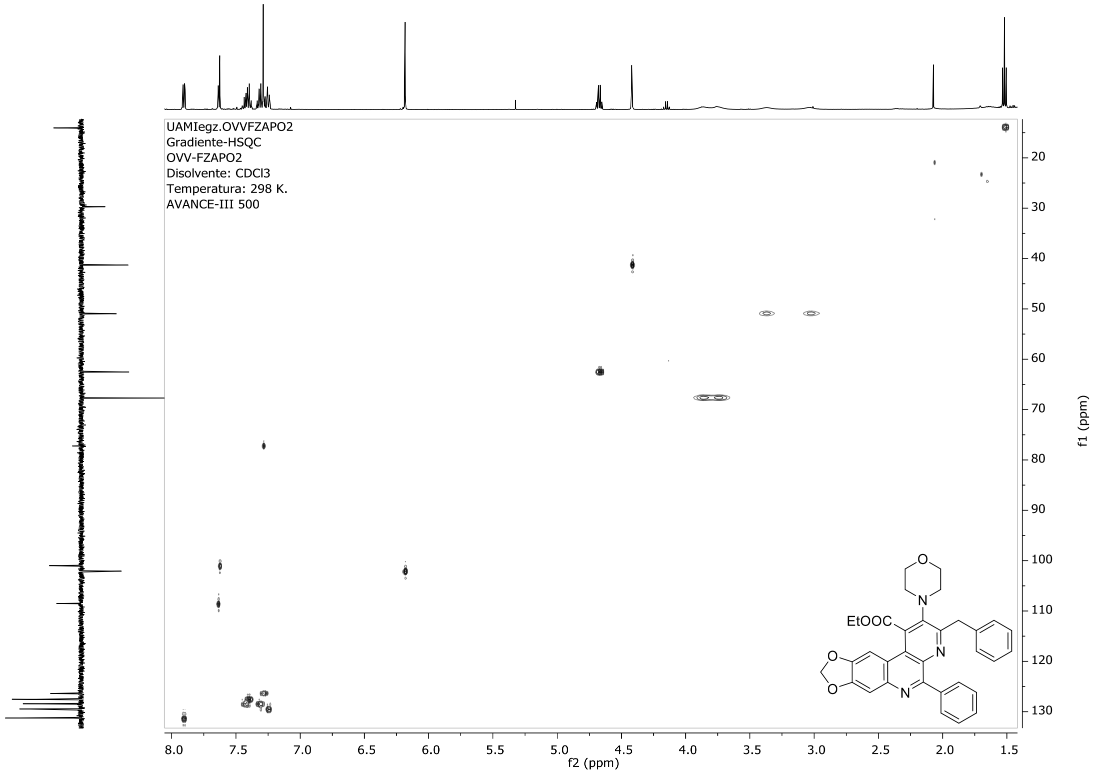

Figure S76. HSQC spectrum of the product **4h**

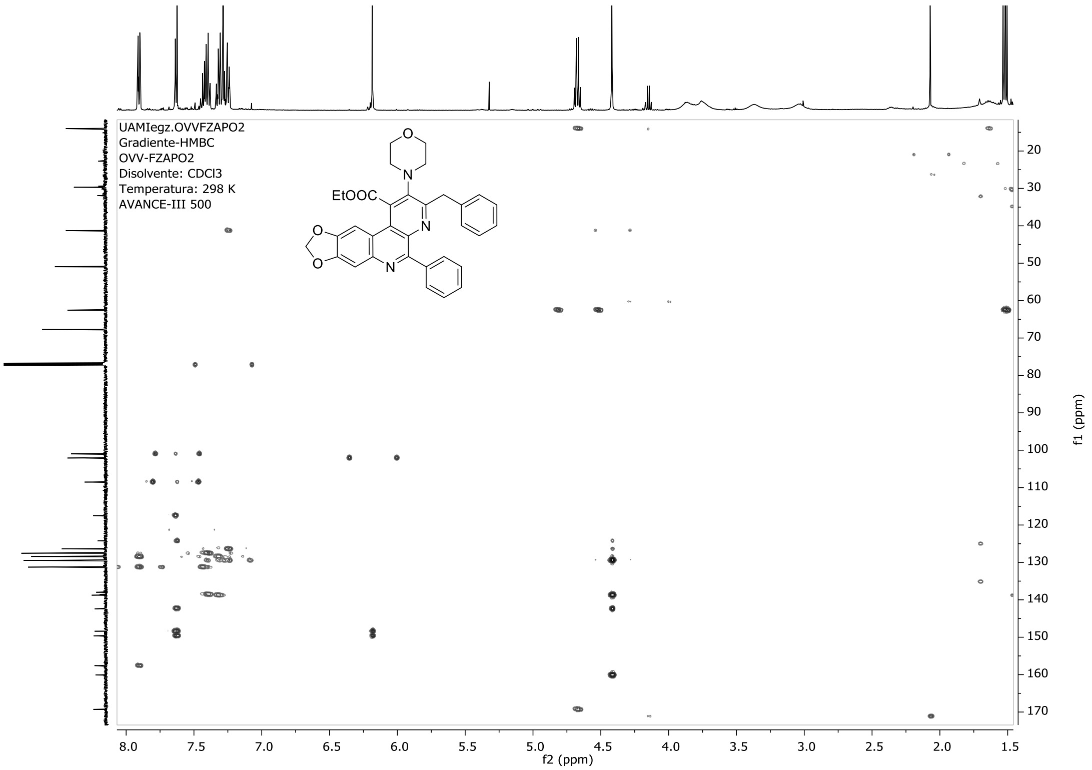

Figure S77. HMBC spectrum of the product **4h**
